# Aberrant ERK signaling in astrocytes impairs learning and memory in RASopathy-associated *BRAF* mutant mouse models

**DOI:** 10.1172/JCI176631

**Published:** 2025-02-18

**Authors:** Minkyung Kang, Jihye Choi, Jeongho Han, Toshiyuki Araki, Soo-Whee Kim, Hyun-Hee Ryu, Min-Gyun Kim, Seoyeon Kim, Hanbyul Jang, Sun Yong Kim, Kyoung-Doo Hwang, Soobin Kim, Myeongjong Yoo, Jaegeon Lee, Kitae Kim, Pojeong Park, Ja Eun Choi, Dae Hee Han, Yujin Kim, Jeongyeon Kim, Sunghoe Chang, Bong-Kiun Kaang, Jung Min Ko, Keun-Ah Cheon, Joon-Yong An, Sang Jeong Kim, Hyungju Park, Benjamin G. Neel, Chul Hoon Kim, Yong-Seok Lee

**Affiliations:** 1 Department of Physiology, and; 2 Department of Biomedical Sciences, Seoul National University College of Medicine, Seoul, Republic of Korea.; 3 Department of Pharmacology, Graduate School of Medical Science, Brain Korea 21 Project, Yonsei University College of Medicine, Seoul, Republic of Korea.; 4 Research Group of Neurovascular Unit, Korea Brain Research Institute, Daegu, Republic of Korea.; 5 Laura and Isaac Perlmutter Cancer Center, New York University Langone Medical Center, New York, New York, USA.; 6 Department of Integrated Biomedical and Life Science, and; 7 BK21FOUR R&E Center for Learning Health Systems, Korea University, Seoul, Republic of Korea.; 8 School of Biological Sciences, Seoul National University, Seoul, Republic of Korea.; 9 Emotion, Cognition and Behavior Research Group, Korea Brain Research Institute, Daegu, Republic of Korea.; 10 Department of Pediatrics, Division of Clinical Genetics, Seoul National University College of Medicine, Seoul, Republic of Korea.; 11 Rare Disease Center, Seoul National University Hospital, Seoul, Republic of Korea.; 12 Department of Child and Adolescent Psychiatry, Severance Hospital, and; 13 Institute of Behavioral Science in Medicine, Yonsei University College of Medicine, Yonsei University Health System, Seoul, Republic of Korea.; 14 Neuroscience Research Institute, Medical Research Center, Seoul National University, Seoul, Republic of Korea.

**Keywords:** Development, Neuroscience, Genetic diseases, Intellectual disability, Neurodevelopment

## Abstract

RAS/MAPK pathway mutations often induce RASopathies with overlapping features, such as craniofacial dysmorphology, cardiovascular defects, dermatologic abnormalities, and intellectual disabilities. Although B-Raf proto-oncogene (*BRAF*) mutations are associated with cardio-facio-cutaneous (CFC) syndrome and Noonan syndrome, it remains unclear how these mutations impair cognition. Here, we investigated the underlying neural mechanisms using several mouse models harboring a gain-of-function BRAF mutation (K499E) discovered in RASopathy patients. We found expressing BRAF K499E (KE) in neural stem cells under the control of a Nestin-Cre promoter (Nestin;BRAF^KE/+^) induced hippocampal memory deficits, but expressing it in excitatory or inhibitory neurons did not. BRAF KE expression in neural stem cells led to aberrant reactive astrogliosis, increased astrocytic Ca^2+^ fluctuations, and reduced hippocampal long-term depression (LTD) in mice. Consistently, 3D human cortical spheroids expressing BRAF KE also showed reactive astrogliosis. Astrocyte-specific adeno-associated virus–BRAF KE (AAV-BRAF KE) delivery induced memory deficits and reactive astrogliosis and increased astrocytic Ca^2+^ fluctuations. Notably, reducing extracellular signal-regulated kinase (ERK) activity in astrocytes rescued the memory deficits and altered astrocytic Ca^2+^ activity of Nestin;BRAF^KE/+^ mice. Furthermore, reducing astrocyte Ca^2+^ activity rescued the spatial memory impairments of BRAF KE–expressing mice. Our results demonstrate that ERK hyperactivity contributes to astrocyte dysfunction associated with Ca^2+^ dysregulation, leading to the memory deficits of *BRAF*-associated RASopathies.

## Abstract 2

A RASopathy-associated gain-of-function BRAF mutation induces hippocampal memory deficits through elevated ERK activity and aberrant calcium signals in reactive astrocytes.

## Graphical abstract

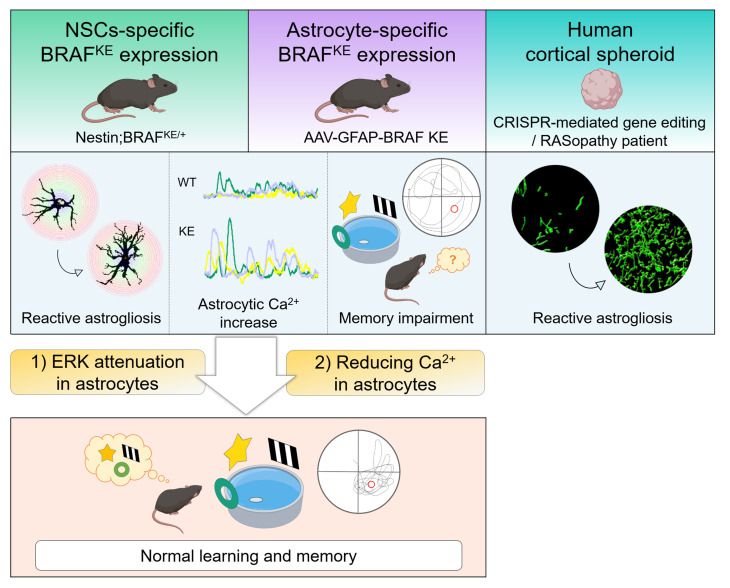

## Introduction

The RAS–extracellular signal-regulated kinase (RAS/ERK) pathway is a highly conserved signaling cascade that transduces extracellular signals from membrane receptors to the nucleus via a series of protein-protein interactions and phosphorylation steps (1–3). Since the RAS/ERK signaling pathway regulates a host of biological processes, including neural development and synaptic function, mutations in RAS/ERK components can cause a range of developmental disorders, referred to as RASopathies (3–6). RASopathies include neurofibromatosis, Noonan syndrome, Costello syndrome, cardio-facio-cutaneous (CFC) syndrome, LEOPARD syndrome, etc. These share some common clinical symptoms, including growth delay, craniofacial dysmorphism, cardiac defects, and neurological impairments that are often accompanied by cognitive deficits (7). But each RASopathy is also characterized by its own distinct symptoms, depending on the causative mutation (8). Indeed, previous studies using animal models have linked specific RASopathy-associated mutations to gene-specific phenotypes associated with the disruption of distinct mechanisms in the central nervous system (9). For example, each disease-specific mutation can affect distinct neuronal cell types (10–14). Therefore, understanding the molecular and cellular mechanisms affected by specific mutations is essential for the development of therapeutic interventions for RASopathies.

The B-Raf proto-oncogene (*BRAF*) encodes a serine/threonine protein kinase that is a direct downstream effector of RAS and an upstream regulator of ERK. Mutations in the *BRAF* gene are frequently associated with RASopathies including CFC syndrome and Noonan syndrome (15–17). CFC syndrome is a particularly severe RASopathy with a high prevalence of intellectual disability (16). Mouse models harboring *Braf* mutations recapitulate several developmental problems associated with CFC syndrome, including craniofacial dysmorphism, cardiac abnormalities, and growth delay (18–21). In addition, studies of *Braf-*deficient mice have implicated *Braf* in neuronal development and cognitive functions, such as learning and memory (22, 23). The mechanisms underlying these cognitive deficits observed in *BRAF*-associated RASopathies, however, remain largely unknown.

Previous studies in model organisms showed that RASopathy-linked mutations are associated with abnormal astrogliogenesis (24–27). For example, mice expressing mutant RAS/MAPK pathway proteins, such as MEK1 Y130C or RAF1 L613V, showed an increased number of glial cells in the cortex and hippocampus (24, 27). The *Nf1* gene encodes neurofibromin 1, which is a negative regulator of the RAS/MAPK pathway. Deletion of *Nfl1* in neural stem/progenitor cells increased astrogliogenesis in both the neonatal period and adulthood (28, 29). Interestingly, neuron-specific ablation of *Nf1* also increased astrogliogenesis, demonstrating that *Nf1*-deficient neurons induce astroglial hypertrophy through a paracrine effect (30). Further, human-induced pluripotent stem cells (iPSCs) derived from Costello syndrome patients that express HRAS G12S differentiated into astroglia more rapidly than control cells (26). This was consistent with mice expressing Costello syndrome–associated *Hras* mutations who showed premature gliogenesis and reduced neurogenesis (26, 31). Astrocytes are required in several key physiological processes in the central nervous system, and there is evidence suggesting the involvement of astrocytic dysfunction, such as aberrant glial activation, in neurological disorders (32–34). Although several studies have reported abnormal astrogliosis in RASopathy models (24, 26–28), it remains unclear how astrocytic dysregulation is related to the cognitive deficits associated with RASopathies.

To address this knowledge gap, we asked whether BRAF K499E (KE), a constitutively active mutation associated with multiple RASopathies, affects astrocytes and contributes to learning and memory deficits in mice. The KE mutation in the BRAF gene, like A246P, Q257R, G469E, L485F, E501K, E501G, N581D and L597V, is a missense variant identified in individuals with CFC syndrome, but it was also reported in association with Noonan syndrome (15, 35, 36). In previous genetic studies of CFC patients, KE mutations seemed to appear at a frequency of 4% across reports. This frequency estimate is unreliable, however, as all the KE mutations detected to date have been de novo mutations discovered in studies with small sample sizes (16, 37, 38). It is noteworthy, however, that patients harboring BRAF KE mutations exhibit severe intellectual disability (16). Moreover, the KE mutation leads to a greater increase in ERK activity than other BRAF mutations (36), especially in neurons (37). Since the BRAF KE mutation induces more marked increases in ERK activity and more severe cognitive disabilities in RASopathy patients, we deemed it worthy of investigation in hopes that the identification of the neurobiological disease mechanisms triggered by this mutation will contribute to our understanding of the cognitive impairments associated with RASopathies. In this study, we used conditional knockin mice, adeno-associated virus (AAV) vectors, and human cortical spheroids expressing BRAF KE to implicate astrocyte dysfunction due to ERK hyperactivity in the learning and memory deficits of *BRAF* mutation-associated RASopathies.

## Results

### BRAF KE expression in either excitatory neurons or inhibitory neurons does not impair learning and memory.

**Figure 1:**
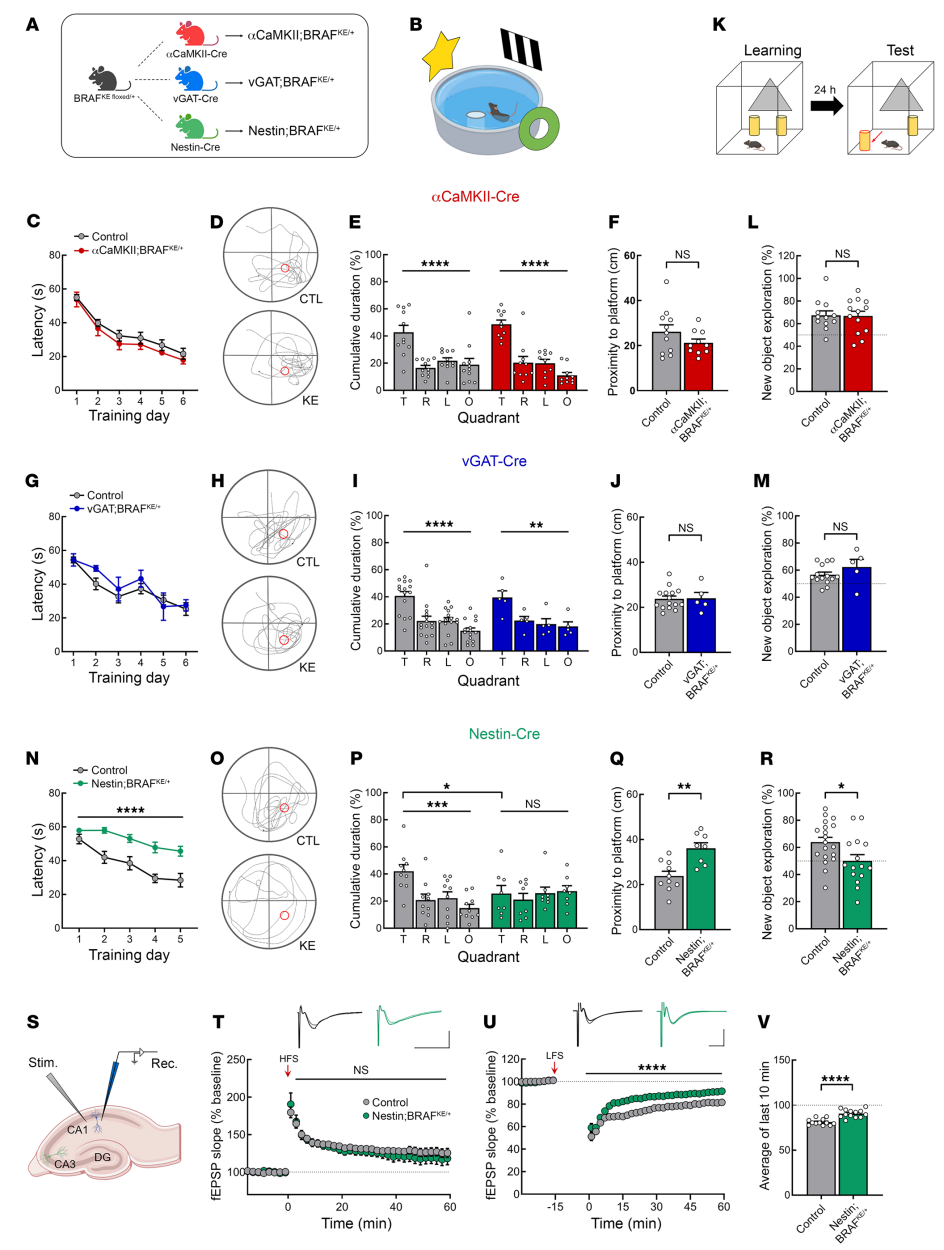
BRAF KE in neural stem cells causes learning deficits and impairs hippocampal LTD. (**A**) Breeding strategy. (**B**) Illustration of the hidden-platform version of the MWM. (**C**) Latency to find the platform during the MWM training trials. (**D**) Representative trajectories of mice in **C** in the MWM probe trial. The platform position during the training trials is indicated by a red circle. (**E**) Quadrant occupancy or (**F**) proximity to the platform of the mice in **D**. T, target; R, right; L, left; O, opposite. (**G**) Latency to find the platform during the MWM training trials. (**H**) Representative trajectories of mice in **G** in the probe trial. (**I**) Quadrant occupancy or (**J**) proximity to the platform of the mice in **H**. (**K**) Schematic of the OPR test. (**L** and **M**) Percentage of time exploring the relocated object in the OPR test for control or (**L**) αCaMKII;BRAF^KE/+^, or (**M**) vGAT;BRAF^KE/+^ mice. (**N**) Latency to find the platform during the MWM training trials. (**O**) Representative trajectories of mice in **N** in the MWM probe trial. (**P**) Quadrant occupancy or (**Q**) proximity to the platform of the mice in **O**. (**R**) Percentage of time exploring the relocated object in the OPR test for control or Nestin;BRAF^KE/+^ mice. (**S**) Schematic showing the slice stimulation/recording configuration. (**T**) LTP induced by HFS. Traces represent the average fEPSP at baseline (–15 to 0 minutes) and after LTP induction (51 to 60 minutes). Vertical bar, 0.5 mV; horizontal bar, 10 ms. (**U**) LTD induced by LFS. Traces represent the average fEPSP at baseline (–15 to 0 minutes) and after LTD induction (51 to 60 minutes). Vertical bar, 1 mV; horizontal bar, 5 ms. (**V**) The average fEPSP slope from 51 to 60 minutes after LTD induction. Data are expressed as means ± SEM. *P < 0.05; **P < 0.01; ***P < 0.001; ****P < 0.0001.

A previous study found that deletion of *Braf* in mouse forebrain excitatory neurons impaired hippocampal learning and memory, suggesting BRAF plays a critical role in excitatory neurons (22). A more recent study, however, showed that the expression of BRAF in the mouse hippocampus is higher in inhibitory neurons than excitatory neurons (10). To determine whether the BRAF KE allele impairs learning and memory in mice and to identify the specific cell type responsible for any learning deficits associated with the *Braf* mutation, we generated inducible BRAF KE knockin mice in which the expression of the KE variant is under the control of Cre-dependent recombination (Supplemental Figure 1A; supplemental material available online with this article; https://doi.org/10.1172/JCI176631DS1). These floxed BRAF KE mice were then crossed with 3 different Cre transgenic mouse lines: αCaMKII-Cre (for expression in excitatory neurons, αCaMKII;BRAF^KE/+^), vGAT-Cre (for expression in inhibitory neurons, vGAT;BRAF^KE/+^), and Nestin-Cre (for expression in neural stem cells, Nestin;BRAF^KE/+^) (Figure 1A). To validate the cell type specificity of the αCaMKII-Cre and vGAT-Cre lines, we injected hSyn-DIO-mCherry virus into the hippocampal CA1 region of each mouse line. We observed the mCherry signal in the pyramidal cell layer of the hippocampal CA1 region in αCaMKII-Cre mice, but not vGAT-Cre mice. In vGAT-Cre mice but not αCaMKII-Cre mice, we observed strong colocalization of the mCherry signal with γ-aminobutyric acid (GABA). This indicates that each Cre promoter drives the expected cell type–specific expression of mCherry (Supplemental Figure 2). We confirmed the heterozygous A-to-G mutation in each knockin line using Sanger sequencing (Supplemental Figure 1, B–D). In addition, because the A-to-G mutation generates a novel BstBI restriction site (TTCGAA), we digested mutant BRAF cDNAs with BstBI to confirm the Cre-dependent recombination and resulting replacement of WT exon 11 with one containing the mutation (Supplemental Figure 1, E–G). We also found a significant increase in p-ERK1/2 expression in αCaMKII;BRAF^KE/+^ hippocampi and a small but insignificant increase in vGAT;BRAF^KE/+^ hippocampi, likely due to the relatively small proportion of hippocampal inhibitory neurons (Supplemental Figure 3, A–D). In the striatum of vGAT;BRAF^KE/+^ mice, however, which has more inhibitory neurons than the hippocampus, we found a significant increase in p-ERK1/2 expression (Supplemental Figure 3, C and E). These results demonstrate the cell type–selective functional expression of the BRAF KE mutation in αCaMKII;BRAF^KE/+^ and vGAT;BRAF^KE/+^ mice.

To evaluate spatial learning and memory in mice expressing BRAF KE either in excitatory or inhibitory neurons, we first subjected αCaMKII;BRAF^KE/+^ and vGAT;BRAF^KE/+^ mice to the hidden-platform version of the Morris water maze (MWM) (Figure 1B). During the training sessions, αCaMKII;BRAF^KE/+^ and vGAT;BRAF^KE/+^ mice showed no significant differences in latency to find the hidden platform compared with littermate controls (BRAF^+/+^, BRAF^KE^ ^floxed/+^, αCaMKII-Cre^+^ or vGAT-Cre^+^) (Figure 1, C and G). We next performed probe trials, in which mice are subjected to the MWM in the absence of the platform. Contrary to our expectation, αCaMKII;BRAF^KE/+^ and vGAT;BRAF^KE/+^ mice showed performance similar to that of control mice at remembering the platform location (Figure 1, D–F and H–J, and Supplemental Figure 4, A, B, G, and H) with comparable swimming speed (Supplemental Figure 4, C and I). Next, the mice were subjected to the object-place recognition (OPR) test, which is another hippocampal-dependent learning and memory task (Figure 1K). Twenty-four hours after training, both αCaMKII;BRAF^KE/+^ and vGAT;BRAF^KE/+^ mice showed a preference toward the relocated object, which was not significantly different from that of control littermates, confirming that spatial memory is intact in both sets of mutant mice (Figure 1, L and M). These data suggest that BRAF KE expression either in αCaMKII^+^ excitatory or in vGAT^+^ inhibitory hippocampal neurons does not induce learning and memory deficits associated with RASopathy.

In addition to intellectual disability, individuals with RASopathy that express *BRAF* mutations often display a broad range of behavioral disabilities such as locomotor impairments, increased anxiety, and decreased sociability (39, 40). Neither αCaMKII;BRAF^KE/+^ nor vGAT;BRAF^KE/+^ mutant mice displayed a significant difference compared with littermate controls in total distance moved or time spent in the center zone of a novel open field, indicating normal voluntary locomotive activity and anxiety-like behavior (Supplemental Figure 4, D, E, J, and K). Both αCaMKII;BRAF^KE/+^ and vGAT;BRAF^KE/+^ mice also showed normal sociability by presenting a strong preference for a social target in the 3-chamber social test (Supplemental Figure 4, F and L).

### BRAF KE expression in neural stem cells impairs learning and memory.

Because BRAF KE expression in αCaMKII^+^ excitatory neurons or vGAT^+^ inhibitory neurons did not impair hippocampal-dependent learning and memory, we hypothesized that other cell types (i.e., nonneuronal cells) might be responsible for the mutant *BRAF*-associated deficits in learning and memory observed in RASopathy patients. Therefore, we generated mice expressing the BRAF KE mutation in neural stem cells by crossing BRAF KE–floxed mice with Nestin-Cre mice. In contrast to the αCaMKII or vGAT promoters, the Nestin promoter starts to control genes in neural stem cells during the embryonic period; these cells subsequently differentiate into both neurons and glia (41). Therefore, Nestin;BRAF^KE/+^ mice are an ideal model for developmental diseases such as RASopathy (42, 43). When brain volume and width were measured by sequential MRI, 10-week-old male Nestin;BRAF^KE/+^ mice showed increased brain volume and width, whereas they showed decreased body weight compared with littermate BRAF^+/+^ mice (Supplemental Figure 5, A–D). This finding has parallels with the phenotype of macrocephaly found in CFC syndrome (44, 45). However, the volume of the hippocampus was comparable to that of littermate controls (Supplemental Figure 5, E and F). Of note, while we found a significant increase in p-ERK1/2 in Nestin;BRAF^KE/+^ mice compared with BRAF^+/+^ mice, we did not observe any significant change in the levels of BRAF or phosphorylated S6 (p-S6) in the hippocampus of Nestin;BRAF^KE/+^ mice (Supplemental Figure 3, F and G, and Supplemental Figure 5, G–J).

We next determined whether Nestin;BRAF^KE/+^ mice showed impairment in learning and memory. In contrast to mice expressing BRAF KE in excitatory or inhibitory neurons, Nestin;BRAF^KE/+^ mice showed severe deficits in the MWM task. Nestin;BRAF^KE/+^ mice showed longer escape latencies during training sessions and poor probe trial performance compared with littermate controls (BRAF^+/+^), demonstrating that the mutant mice failed to learn and recall the location of the platform position (Figure 1, N–Q, and Supplemental Figure 4, M and N). In addition, Nestin;BRAF^KE/+^ mice also showed a memory deficit in the OPR task (Figure 1R). Nestin;BRAF^KE/+^ mice showed comparable basal locomotive activity, anxiety-like behavior, and sociability (Supplemental Figure 4, O–R). A previous study reported that Nestin-Cre mice showed deficits in the acquisition of conditioned fear (46). To determine whether Nestin-Cre mice show deficits in spatial learning and memory, we examined their performance in the MWM task. We found that Nestin-Cre mice showed performance similar to that of their WT littermates in the MWM task (Supplemental Figure 6). Taken together, these results demonstrate that Nestin;BRAF^KE/+^ mice recapitulate structural and intellectual deficits associated with RASopathies. Thus, we continued to use this mutant mouse model to investigate the mechanism of learning and memory deficits in RASopathies.

### BRAF KE expression in neural stem cells impairs hippocampal LTD.

Long-term synaptic plasticity is a key cellular mechanism underlying learning and memory (47), and impairments in long-term potentiation (LTP) have been observed in RASopathy mouse models with learning deficits (10, 47, 48). To determine whether Nestin;BRAF^KE/+^ mice show any changes in synaptic plasticity, we recorded field excitatory postsynaptic potentials (fEPSPs) in the hippocampal Schaffer collateral pathway (Figure 1S). Although we did not find any changes in LTP induced via high-frequency stimulation (HFS; one burst consisting of a hundred stimuli at 100 Hz) (Figure 1T), we found that Nestin;BRAF^KE/+^ mice show reduced NMDA receptor–dependent long-term depression (NMDAR-LTD) in response to low-frequency stimulation (LFS; one burst consisting of 900 stimuli at 1 Hz) (Figure 1, U and V). To explore the biochemical mechanisms underlying this LTD impairment, we examined the expression of pre- and postsynaptic proteins in the hippocampi of Nestin;BRAF^KE/+^ via Western blot. Apart from a small increase in GRIN1, we did not detect any dramatic changes in synaptic protein expression in whole cell lysates or membrane fractions derived from the hippocampus of Nestin;BRAF^KE/+^ mice (Supplemental Figure 7).

### BRAF KE drives increased reactive astrogliosis in the hippocampus.

**Figure 2:**
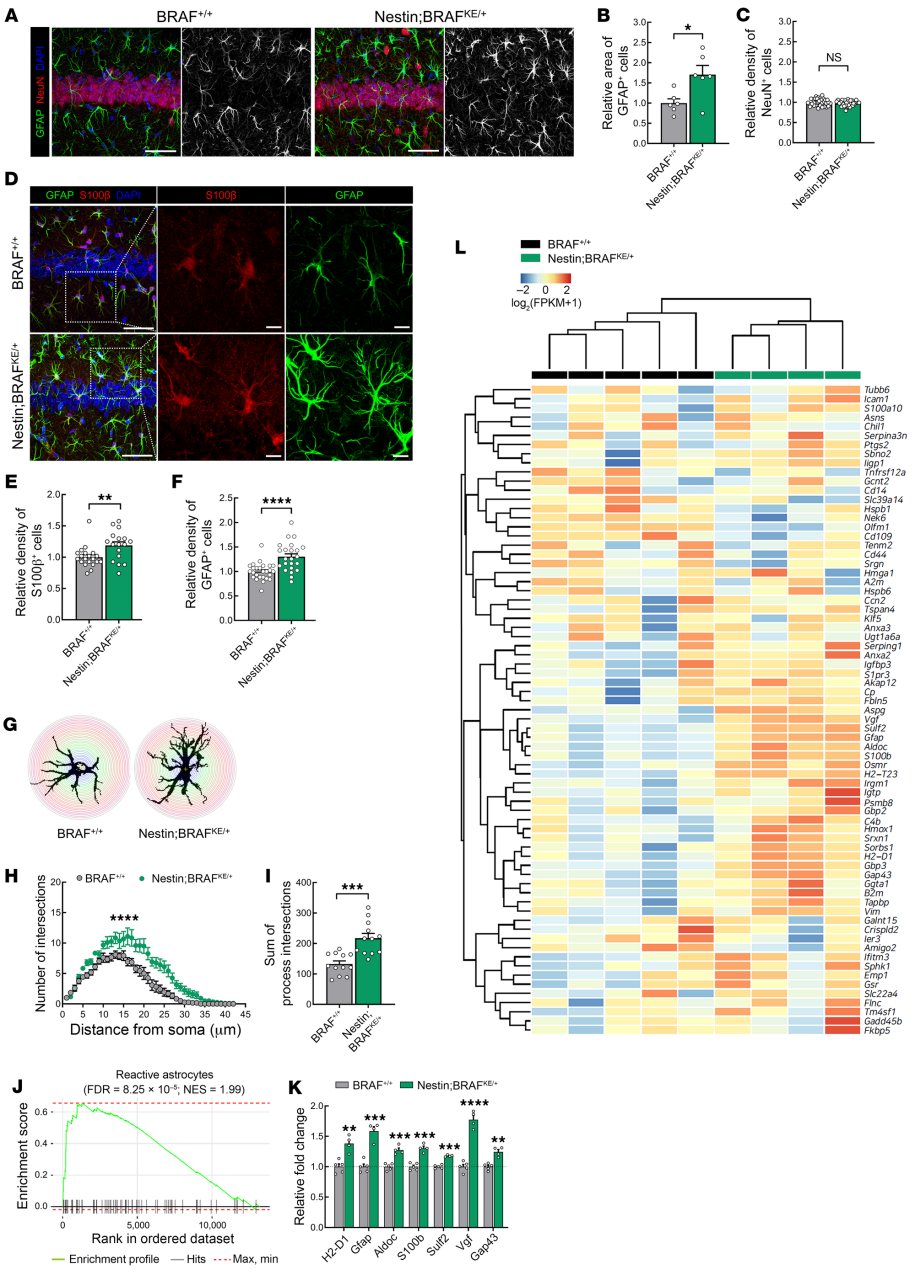
Nestin;BRAF^KE/+^ mice exhibit reactive astrogliosis in the hippocampus. (**A**) Representative images of GFAP (green) and NeuN (red) immunolabeling in the hippocampal CA1 region of adult BRAF*^+/+^* and Nestin;BRAF^KE/+^ mice. Scale bars: 50 μm. (**B**) Relative area of GFAP-expressing cells from mice in **A**. (**C**) Number of NeuN-positive cells from mice in **A**. (**D**) Representative confocal images of S100β (red) and GFAP (green) immunolabeling in the hippocampal CA1 region of adult BRAF^+/+^ and Nestin;BRAF^KE/+^ mice. Scale bars: 50 μm; 10 μm (zoomed in image). (**E** and **F**) Relative density of (**E**) S100β-expressing cells or (**F**) GFAP-expressing cells in the hippocampi from BRAF^+/+^ or Nestin;BRAF^KE/+^ mice. (**G**) Representative image for a Sholl analysis of an astrocyte in the stratum radiatum of the GFAP-stained image in **D**. (**H**) The number of process intersections and (**I**) the sum of the process intersections of GFAP-positive astrocytes in the stratum radiatum of BRAF^+/+^ or Nestin;BRAF^KE/+^ mice. (**J**) Plot showing enrichment of reactive astrocyte gene sets. (**K**) Relative fold change of reactive astrocyte genes in the comparison of BRAF^+/+^ and Nestin;BRAF^KE/+^ transcriptomes. (**L**) Hierarchical clustering heatmap with the expression levels of reactive astrocyte genes represented in colors mapped to log_2_-transformed FPKM plus 1. FPKM, fragments per kilobase of transcript per million mapped reads. Data are expressed as means ± SEM. *P < 0.05; **P < 0.01; ***P < 0.001; ****P < 0.0001.

Individuals with RASopathies, including CFC syndrome, display developmental abnormalities in the central nervous system such as structural malformation, ectopia, and atrophy that could contribute to neurobehavioral phenotypes (8, 49–53). Since several studies also have reported an imbalance in the neuron-glial population in RASopathy-related animal models (24, 27, 28, 54), we investigated whether neuronal or glial cells were affected in Nestin;BRAF^KE/+^ animals. GFAP, a type III intermediate filament, is a marker of astrocytic activation, and the relative area of GFAP^+^ astrocytes was significantly increased in adult (8–15 weeks old) Nestin;BRAF^KE/+^ mice compared with littermate controls (Figure 2, A and B). However, the NeuN^+^ neuronal populations were unaltered in the hippocampal CA1 region (Figure 2, A and C) and the spine density of hippocampal neurons was comparable between genotypes (Supplemental Figure 5, K and L). Recently, several studies have reported that mice with hyperactive or null mutations of genes in the RAS/ERK signaling pathway result in a selective loss of parvalbumin^+^ interneurons or reduction of somatostatin expression (55, 56). Nestin;BRAF^KE/+^ mice, however, showed no difference in the numbers of parvalbumin^+^ or somatostatin^+^ interneurons in the hippocampal CA1 region or the cortex compared with littermate controls. This suggests the BRAF KE mutation did not affect the generation or maintenance of inhibitory neurons, at least in the hippocampus and cortex (Supplemental Figure 8). Due to the lack of distinguishable abnormalities in neuronal populations of Nestin;BRAF^KE/+^ mice, we therefore turned to investigate the effects of the BRAF KE allele on astrocytes.

Reactive astrocytes undergo morphological, molecular, and functional remodeling in response to external stimulus (57) and are commonly detected by expression of GFAP and S100β (58, 59). Notably, the numbers of GFAP^+^ and S100β^+^ cells were both significantly increased in the hippocampus of the mutants, suggesting reactive astrogliosis in the hippocampus of adult Nestin;BRAF^KE/+^ mice (Figure 2, D–F). The number and sum of intersects of GFAP^+^ astrocytes measured by a Sholl analysis were also significantly increased in Nestin;BRAF^KE/+^ mice (Figure 2, G–I). Neuroinflammation is one possibility for the abnormal increase in astrogliosis, which is frequently accompanied by microglial activation (60). However, we found that the number and morphology of Iba1^+^ microglia in the CA1 region of the hippocampus in Nestin;BRAF^KE/+^ mice were not significantly different from those of littermate controls (Supplemental Figure 9, A and B). A similar increase in the relative density and area of GFAP^+^ astrocytes was also found in the cortex, and no change was observed in neuronal density (Supplemental Figure 10, A–C). There was also no significant change in the relative density of Iba1^+^ microglia in the cortex (Supplemental Figure 10, D and E). Similar to hippocampal lysates, p-ERK1/2 expression was also increased in cortical lysates, indicating BRAF KE allele–mediated ERK signaling overactivation also in the cortex (Supplemental Figure 10, F and G). Of note, neither αCaMKII;BRAF^KE/+^ nor vGAT;BRAF^KE/+^ mutant mice exhibited changes in the relative area covered by GFAP^+^ cells in the hippocampus, suggesting the expression of BRAF KE in αCaMKII- or vGAT-positive neurons does not affect astrocyte populations (Supplemental Figure 11).

To investigate the molecular basis of BRAF KE–induced learning and memory deficits as well as astrogliosis in Nestin;BRAF^KE/+^ mice, we performed transcriptomic analyses of hippocampus isolated between 11 and 15 weeks of age. Compared with littermate controls, Nestin;BRAF^KE/+^ mice exhibited a total of 864 differentially expressed genes (DEGs) (false discovery rate [FDR] < 0.05), including 422 upregulated and 442 downregulated genes in the hippocampus (Supplemental Table 1A and Supplemental Figure 12A). To characterize their functional annotations and biological processes, we conducted a gene ontology enrichment analysis on the DEGs (Supplemental Table 1B and Supplemental Figure 12B). We found that DEGs upregulated in Nestin;BRAF^KE/+^ mice were significantly enriched for the MAPK cascade (GO:0000165; FDR = 9.43 × 10^–6^), ERK1 and ERK2 cascade (GO:0070371; FDR = 2.55 × 10^–3^), and cell-cell junction (GO:0005911; FDR = 3.35 × 10^–3^) gene sets. In contrast, DEGs downregulated in Nestin;BRAF^KE/+^ mice were enriched for the response to growth factor (GO0070848; FDR = 7.35 × 10^–6^), tissue morphogenesis (GO:0048729; FDR = 3.66 × 10^–3^), and extracellular matrix (GO:0031012; FDR = 2.27 × 10^–5^) gene sets. Notably, the gene set indicative of reactive astrocytes was significantly enriched in Nestin;BRAF^KE/+^ mice compared with littermate controls (normalized enrichment score [NES] = 1.99, FDR = 8.25 × 10^–5^) (Supplemental Table 1C and Figure 2J). In contrast, the microglial activation gene set was not significantly altered in Nestin;BRAF^KE/+^ mice compared with littermate controls, suggesting local inflammatory responses are unlikely to be the cause of the reactive changes we observed in astrocytes (NES = 0.86, FDR = 7.07 × 10^–1^) (Supplemental Figure 9, C and D). In addition, hierarchical clustering analysis of DEGs between Nestin;BRAF^KE/+^ mice and littermate controls demonstrated that a cluster of upregulated genes in hippocampi of Nestin;BRAF^KE/+^ mice are associated with reactive astrocytes (Figure 2L). In particular, we observed an increase in expression of *H2-D1*, *Gfap*, *Aldoc*, *S100b*, *Sulf2*, *Vgf*, and *Gap43* (Figure 2, K and L). Taken together, these findings suggest that BRAF KE–induced RAF/ERK overactivation stimulates the expression of genes linked to reactive astrocytes.

Severe astrogliosis is characterized, in part, by the proliferating potential of astrocytes, whereas astrocytes with a mild or moderate phenotype show hypertrophy without proliferation (61). The increased number of GFAP^+^ astrocytes observed in Nestin;BRAF^KE/+^ mice could be due to either increased proliferation or altered astrocyte reactivity. To determine whether the proliferation contributed to increased GFAP^+^ astrocytes, we stained brain slices for the mitotic marker Ki67. This showed that adult Nestin;BRAF^KE/+^ mice had comparable numbers of Ki67^+^ cells in the hippocampus and the cortex to those of littermate controls, suggesting that the mutant BRAF does not stimulate astrocyte proliferation (Supplemental Figure 13). We also examined the population of proliferating neural progenitor cells in the subgranular zone (SGZ) of the dentate gyrus by immunolabeling Ki67^+^ and Sox2^+^ cells. We found that the ratio of Ki67^+^-Sox2^+^/Sox2^+^ cells (i.e., proliferating Sox2^+^ cells) in the SGZ was comparable between Nestin;BRAF^KE/+^ and littermate control mice (Supplemental Figure 14). Since previous studies reported an association between hyperactive ERK signaling and changes in oligodendrocyte development (24, 62), we compared the numbers of Olig2^+^ oligodendrocytes in the hippocampus and cortex of Nestin;BRAF^KE/+^ mice to those of littermate controls and found them unchanged (Supplemental Figure 15). These results suggest that the expression of BRAF KE in neural stem cells has a minimal effect on other cell types apart from astrocytes.

We next asked whether the reactive GFAP^+^ astrocytes are formed during early postnatal development or progressively increased during adulthood. We assessed GFAP^+^ astrocytes from mice at postnatal day 20 and found that the relative areas of GFAP^+^ cells in the mutant cortex and hippocampus are comparable to those in littermate controls (Supplemental Figure 16, A–D). Notably, at postnatal day 40, we found that there was a significant increase in the relative area of GFAP^+^ astrocytes in the mutant cortex and hippocampus (Supplemental Figure 16, E–H). These results show that BRAF KE induces reactive changes in astrocytes in a progressive manner.

### BRAF KE induces reactive astrogliosis by activating ERK signaling.

Since p-ERK1/2 expression was found to increase in the hippocampus of Nestin;BRAF^KE/+^ mice (Supplemental Figure 3, F and G), we further explored whether the increased astrogliosis in Nestin;BRAF^KE/+^ mice is due to the ERK activation in astrocytes themselves, not by a cell nonautonomous mechanism. We cultured mouse cortical astrocytes and transfected them with a plasmid expressing CMV-FLAG-BRAF WT or CMV–FLAG–BRAF KE together with a GFP-N1 plasmid. After 48 hours, cells were fixed and immunostained with anti-FLAG and anti-p-ERK1/2 antibodies (Supplemental Figure 17A). We found that BRAF KE–transfected astrocytes showed not only increased cell size (astrocytic hypertrophy), which is measured by the area of GFP, but also increased p-ERK1/2 expression (Supplemental Figure 17, B–D).

Next, we performed a transcriptomic analysis of primary astrocyte cultures to examine the effect of BRAF KE expression on various signaling pathways. Compared with nontransfected astrocytes, BRAF KE–transfected astrocytes exhibited a total of 6,479 DEGs — 3,093 were upregulated and 3,386 were downregulated (Supplemental Table 2A and Supplemental Figure 18A). In an enrichment analysis of the upregulated DEGs, we found significant enrichment in the MAPK cascade (GO:0043410; FDR = 7.01 × 10^–4^), cell-cell adhesion (GO:0098609; FDR = 1.54 × 10^–3^), and neuroinflammatory response (GO:0150076; FDR = 3.94 × 10^–3^) gene sets. In a similar analysis of downregulated DEGs, we found significant enrichment in the inflammatory response (GO:0006954, FDR = 9.89 × 10^–60^) and cytokine production (GO:0001816; FDR = 8.84 × 10^–58^) gene sets (Supplemental Table 2B and Supplemental Figure 18B). Notably, when we performed a hierarchical clustering analysis of the various DEGs, we identified a cluster of upregulated genes in BRAF KE–transfected astrocytes associated with reactive astrocytes (Supplemental Figure 18C). There was also significant enrichment for the reactive astrocyte gene set in BRAF KE–transfected astrocytes compared with nontransfected astrocytes (NES = 2.74, FDR = 2.30 × 10^–4^) (Supplemental Table 2C and Supplemental Figure 18D). Together, these results suggest that BRAF KE expression in mouse primary astrocytes increases the expression of genes associated with reactive astrocytes and with the MAPK pathway by a cell-autonomous mechanism.

### BRAF KE activates RAS/ERK signaling in astrocytes and induces reactive astrogliosis in human cortical spheroids.

**Figure 3:**
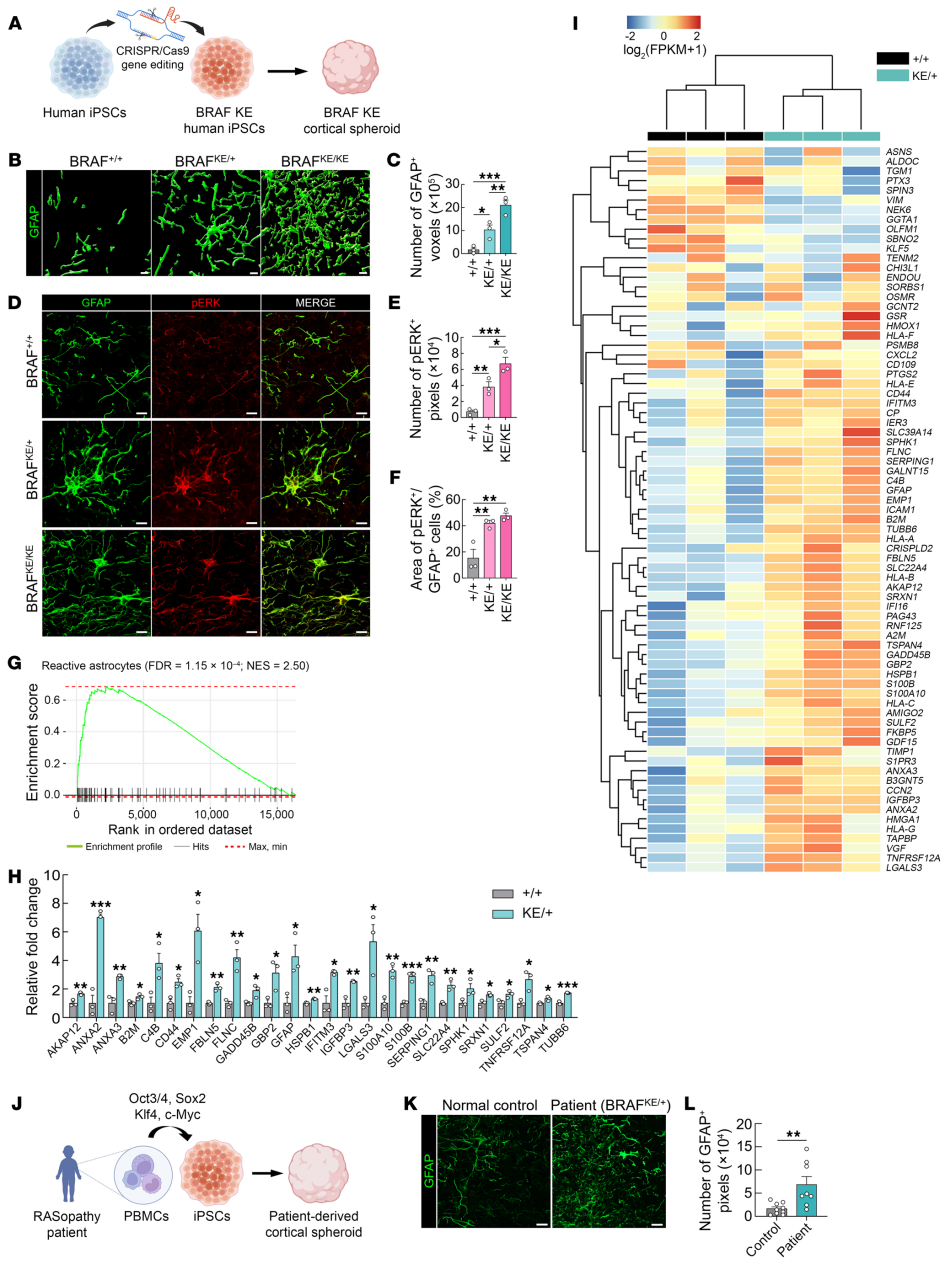
BRAF KE activates RAS/ERK signaling in astrocytes and induces reactive astrogliosis in human cortical spheroids. (**A**) Schematic diagram of the experimental design. (**B**) Representative 3D surface renderings of GFAP-positive cells in BRAF*^+/+^*, BRAF^KE/+^, and BRAF^KE/KE^ human cortical spheroids at day 372. Scale bars: 10 μm. (**C**) Quantification of the number of GFAP-positive voxels in human cortical spheroids in **B**. (**D**) Representative images of p-ERK1/2 (red) and GFAP (green) immunostaining in BRAF^+/+^, BRAF^KE/+^, and BRAF^KE/KE^ human cortical spheroids at day 292. Scale bars: 10 μm. (**E** and **F**) Quantification of (**E**) the number of p-ERK1/2-positive pixels (area) and (**F**) percentage of the p-ERK1/2-positive area out of the GFAP-positive area in human cortical spheroids in **D**. (**G**) Plot showing enrichment of reactive astrocyte gene sets. (**H**) Relative fold changes in the expression of reactive astrocyte genes in the comparison between the BRAF^+/+^ and BRAF^KE/+^ transcriptomes. (**I**) Hierarchical clustering heatmap showing the expression of reactive astrocyte gene colors mapped to log_2_-transformed FPKM plus 1. (**J**) Schematic diagram of the experimental design. (**K**) Representative images of GFAP immunolabeling of human cortical spheroids at day 372. iPSC lines were generated from a RASopathy patient with the BRAF^KE/+^ mutation and from an age/sex-matched normal subject. These lines were then differentiated into 3D cortical spheroids. Scale bars: 20 μm. (**L**) Quantification of GFAP-positive pixels in cortical spheroids from normal control and RASopathy patient. Data are expressed as means ± SEM. *P < 0.05; **P < 0.01; ***P < 0.001.

To model cellular abnormalities associated with the *BRAF* mutation and determine whether BRAF KE–related reactive astrogliosis observed in BRAF KE mice is also present in human brain cells, we differentiated iPSCs into 3D human cortical spheroids (63), which generate neuronal and glial cells. To express the human BRAF KE variant, we generated mutant human iPSC lines using the CRISPR approach (64). We established independent but isogenic clones (i.e., WT, BRAF KE heterozygous, or BRAF KE homozygous) derived from the same iPSC line (Figure 3A and Supplemental Figure 19A). First, we measured GFAP expression in cortical spheroids cultured for 372 days using 3D surface renderings. Consistent with the data from Nestin;BRAF^KE/+^ mice, GFAP expression was significantly increased in BRAF KE heterozygous and homozygous spheroids (Figure 3, B and C). We also independently differentiated the same iPSC lines, which reproduced similar results (Supplemental Figure 19, B and C), suggesting that the generation of reactive astrocytes is highly reproducible across differentiations of the same iPSC line. BRAF KE spheroids also showed increased immunoreactivity to S100β compared with WT spheroids, corroborating the finding that the BRAF KE mutation induces astrogliosis (Supplemental Figure 19, D and E). In addition, GFAP immunostaining in a different set of isogenic cell lines also showed similar results (Supplemental Figure 19, F and G). We also found that there are few Ki67^+^ cells in BRAF KE spheroids, suggesting that BRAF KE does not stimulate astrocyte proliferation also in human spheroid models (Supplemental Figure 19H). Furthermore, in BRAF KE cortical spheroids, we found that most p-ERK1/2^+^ cells overlapped with GFAP^+^ astrocytes, indicating BRAF KE stimulates ERK signaling (Figure 3, D–F). Intriguingly, we found that glial scar–like structures, which are characterized by a dense wall of reactive astrocytes with fibrotic materials, occur only in BRAF KE homozygous spheroids, suggesting that some astrocytes with the BRAF KE mutation in the cortical spheroid indeed undergo very intense reactive astrogliosis (Supplemental Figure 19, I and J).

We further performed transcriptomic analyses of WT and BRAF KE heterozygous spheroids, which revealed differential expression of 5,727 genes (FDR < 0.05), including 2,948 upregulated and 2,779 downregulated DEGs (Supplemental Table 3A and Supplemental Figure 20A). In an enrichment analysis of DEGs upregulated in BRAF KE heterozygous spheroids, we found significant enrichment in the gene sets associated with the MAPK cascade (GO:0000165; FDR = 3.77 × 10^–2^), extracellular matrix organization (GO:0030198; FDR = 1.07 × 10^–10^), and cell-cell adhesion (GO:0098609; FDR = 1.11 × 10^–4^) (Supplemental Table 3B and Supplemental Figure 20B). DEGs downregulated in BRAF KE heterozygous spheroids were enriched in gene sets associated with chromosome segregation (GO:0007059; FDR = 9.29 × 10^–27^), mitotic cell cycle (GO:0000278; FDR = 2.82 × 10^–21^), and microtubule binding (GO:0008017; FDR = 3.76 × 10^–8^) (Supplemental Table 3B and Supplemental Figure 20B). Upregulated DEGs identified in BRAF KE spheroids showed a significant overlap with genes that are altered in reactive astrocytes (NES = 2.50, FDR = 1.15 × 10^–4^) (Supplemental Table 3C and Figure 3G). In addition, hierarchical clustering analysis revealed that upregulated DEGs with similar expression pattern among BRAF KE spheroids are associated with reactive astrocytes. In particular, 26 of these genes were significantly overexpressed in BRAF KE spheroids relative to WT spheroids (Figure 3, H and I). Last, we also examined iPSC-derived cortical spheroids from a RASopathy patient with a BRAF^KE/+^ mutation (Figure 3J and Supplemental Figure 19K). Consistently, we found that GFAP expression was significantly higher in cortical spheroids from the RASopathy patient compared with those from a normal control (Figure 3, K and L). Collectively, these results indicate that the reactive astrogliosis induced by overactivation of astrocytic ERK signaling could be a neuropathological mechanism of *BRAF*-linked genetic disorders.

### Expressing BRAF KE in adult astrocytes impairs learning and memory.

**Figure 4:**
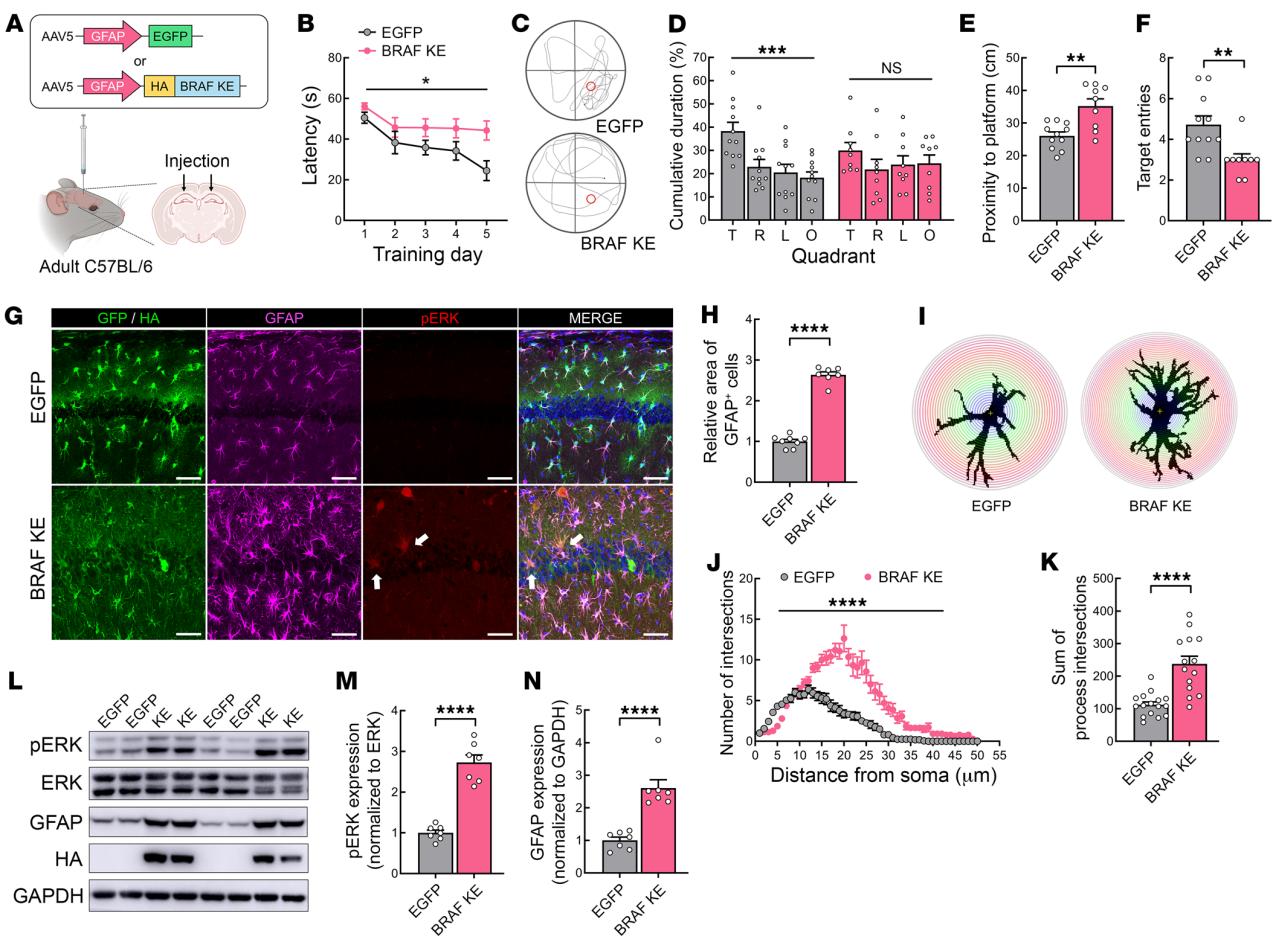
Astrocyte-specific BRAF KE induces reactive astrogliosis and hippocampal memory deficits. (**A**) Schematic illustrating the experimental approach. AAV5-GFAP-GFP (GFP) or AAV5–GFAP–HA–BRAF KE (BRAF KE) was injected into the hippocampal CA1 region of adult C57BL/6 mice. (**B**) Latency for mice injected with GFP or BRAF KE to find the hidden-platform during MWM training trials. (**C**) Representative trajectories of GFP- or BRAF KE–injected mice during the MWM probe trial. (**D**) Quadrant occupancy, (**E**) proximity to the platform, or (**F**) number of target zone entries of the mice in **C**. (**G**) Representative immunohistochemical images from GFP- or BRAF KE–injected mice. Slices were immunostained for HA (green), GFAP (magenta), and p-ERK1/2 (red). Arrows indicate double labeling of GFAP and p-ERK1/2. Scale bars: 50 μm. (**H**) GFAP-expressing cells from the mice in **G**. (**I**) Representative Sholl analyses of an astrocyte in the stratum radiatum of the GFAP-stained image in **G**. (**J**) The number of process intersections and (**K**) the sum of process intersections for GFAP-positive astrocytes in the stratum radiatum of GFP- or BRAF KE–injected mice. (**L**) Representative immunoblot image of hippocampal lysates from mice injected with GFP or BRAF KE. (**M**) p-ERK1/2 expression normalized to ERK1/2 expression in hippocampal lysates or (**N**) GFAP expression normalized to GAPDH expression from the mice in **L**. Data are expressed as means ± SEM. *P < 0.05; **P < 0.01; ***P < 0.001; ****P < 0.0001.

Since we found that reactive GFAP^+^ astrocytes are progressively increased during postnatal days, we examined whether BRAF KE expression in adult astrocytes is sufficient to induce memory deficits in mice. We generated an AAV5 vector encoding BRAF KE fused to HA tag which is expressed under the control of the astrocyte-specific GFAP promoter AAV5–GFAP–HA–BRAF KE (GFAP-BRAF KE). Adult C57BL/6 mice received injections of GFAP-BRAF KE virus or a control virus, AAV5-GFAP-EGFP (GFAP-EGFP), into the dorsal hippocampus (Figure 4A). We confirmed that the transduction of the AAV-delivered constructs was highly selective to astrocytes in the hippocampus, finding that over 93% of the infected cells (EGFP^+^ or HA–BRAF KE^+^ cells) were GFAP^+^ astrocytes (Supplemental Figure 21). First, we compared the performance of GFAP–EGFP– or GFAP–BRAF KE–injected mice in MWM to investigate the effects of astrocytic BRAF KE expression on hippocampal-dependent spatial learning and memory. Compared with mice injected with GFAP-EGFP virus, mice injected with GFAP–BRAF KE virus took longer to reach the hidden-platform during the training sessions (Figure 4B). When spatial memory was assessed in probe trials, GFAP-EGFP virus–injected mice preferred the target quadrant, whereas GFAP–BRAF KE virus–injected mice did not show a selective preference toward the target quadrant over the other 3 quadrants (Figure 4, C and D). Moreover, GFAP–BRAF KE virus–injected mice searched farther from the target and less frequently visited the target quadrant than GFAP-EGFP virus–injected mice, indicating that mice injected with GFAP–BRAF KE virus in the hippocampus exhibit spatial learning impairment (Figure 4, E and F).

We also noted that the area of GFAP^+^ astrocytes was significantly increased in the hippocampus of GFAP–BRAF KE virus–injected mice relative to GFAP-EGFP virus–injected mice (Figure 4, G and H), and the probability of detecting p-ERK1/2 immunosignals was also higher in BRAF KE–expressing astrocytes (Figure 4G). Although we observed increased p-ERK1/2 signals in cell types other than GFAP^+^ astrocytes, we showed that GFAP–BRAF KE expression is highly restricted to astrocytes. This suggests that the p-ERK1/2 signals we detected in other cell types are unlikely to be a result of leaky AAV expression. Instead, we suspect these signals either represent basal activity or indicate the influence of neighboring reactive astrocytes. Sholl analysis showed that GFAP–BRAF KE virus–injected mice had a significant increase in the number of astrocyte intersections, suggesting increased cellular complexity (Figure 4, I–K). Consistent with the results of histological immunostaining, Western blotting showed that p-ERK1/2 protein levels were significantly higher in the hippocampus of GFAP–BRAF KE virus–injected mice compared with controls, confirming the expression of GFAP–BRAF KE virus overactivates the ERK signaling pathway in vivo (Figure 4, L and M). GFAP protein expression analyzed in Western blotting was also increased in GFAP–BRAF KE virus–injected hippocampi (Figure 4, L and N). Collectively, these results show that expressing the BRAF KE mutation in adult astrocytes can recapitulate the cellular and behavioral deficits observed in Nestin;BRAF^KE/+^ mice, suggesting that BRAF KE mutation alters the function of astrocytes by inducing reactive astrogliosis, which leads to impairment of hippocampal learning.

### BRAF KE–expressing astrocytes show elevated calcium fluctuations that are normalized by attenuating RAS/ERK signaling.

Astrocytic Ca^2+^ signaling mediates gliotransmitter release, synaptic plasticity, and cell integrity, thus directly modulating neural circuits (65, 66). However, it remains largely unknown whether the Ca^2+^ activity in astrocytes is implicated in the pathophysiology of RASopathy. Therefore, we set out to test the presumption that Ca^2+^ activity might be dysregulated in BRAF KE–expressing astrocytes. We used an astrocyte-selective virus expressing a fast and sensitive Ca^2+^ indicator, AAV5-gfaABC1D-GCaMP6f (gfaABC1D-GCaMP6f). We injected the virus into the dorsal CA1 region of adult hippocampus where GFAP–BRAF KE virus was injected (Supplemental Figure 22A). GCaMP6f imaging revealed that the peak frequency of Ca^2+^ fluctuations in each region of interest (ROI) was significantly increased in BRAF KE–expressing astrocytes, indicating that BRAF KE expression leads to aberrantly hyperactive Ca^2+^ activity in hippocampal astrocytes (Supplemental Figure 22, B–D).

**Figure 5:**
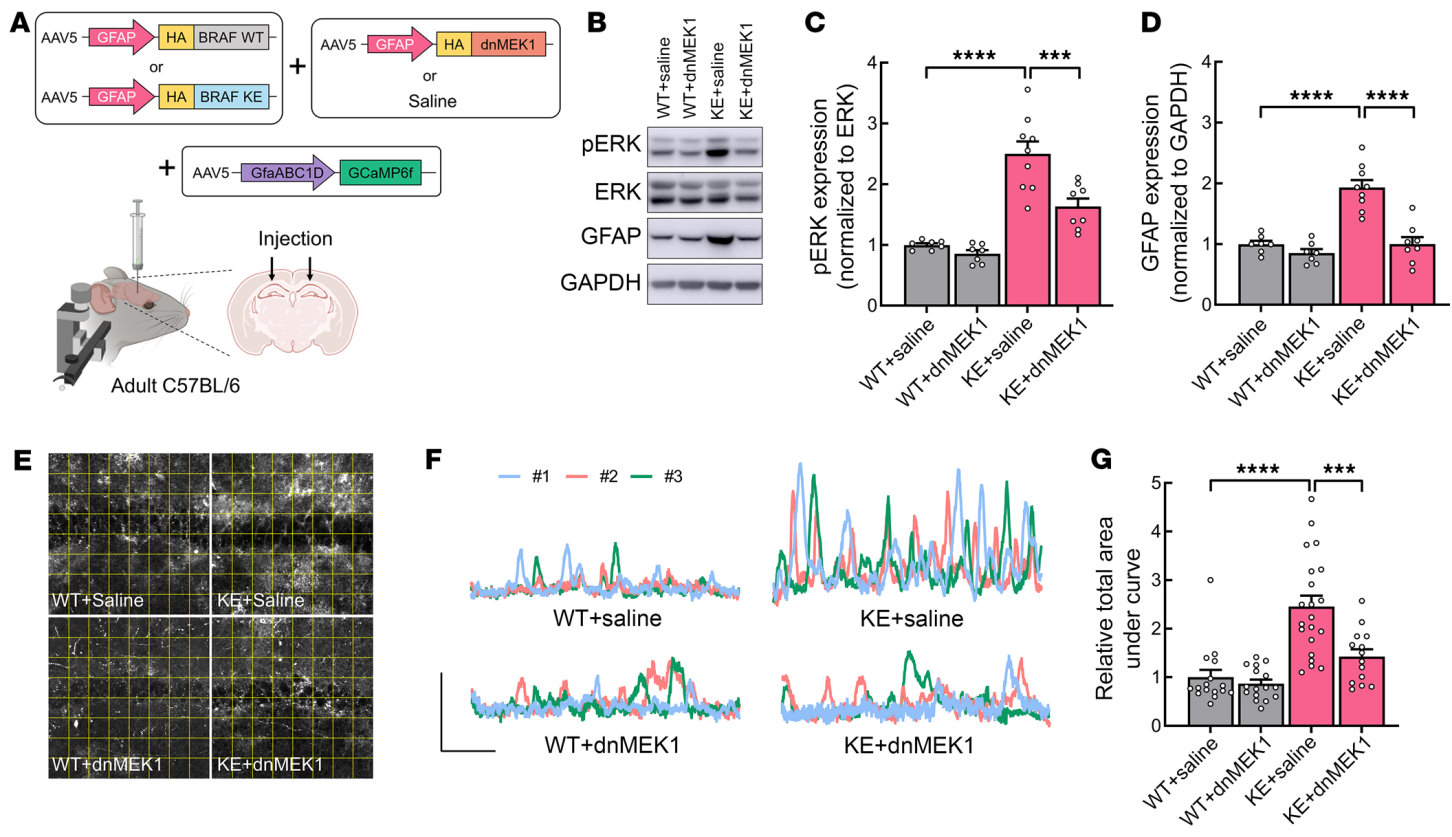
BRAF KE–induced hyperactive calcium fluctuations in hippocampal astrocytes are dependent on RAS/ERK signaling. (**A**) Schematic illustrating the experimental approach. The hippocampal CA1 regions of adult C57BL/6 WT mice were injected with AAV-GFAP-HA-BRAF WT (WT) or AAV–GFAP–HA–BRAF KE (KE), and GFAP-HA-dnMEK1 (dnMEK1) or saline, and AAV-gfaABC1D-GCaMP6f. (**B**) Representative Western blot of p-ERK1/2, ERK1/2, GFAP, and GAPDH expression in hippocampal lysates from mice injected as in **A**. (**C**) p-ERK1/2 expression normalized to ERK1/2 expression or (**D**) GFAP expression normalized to GAPDH expression in mice treated as in **A**. (**E**) Representative images of Ca^2+^ fluctuations measured in hippocampal astrocytes from BRAF WT or BRAF KE mice injected with saline or dnMEK1. The yellow squares indicate each ROI. (**F**) Representative GCaMP fluorescent traces from the astrocytes in **E**. Scale bars: 20 arbitrary units and 1 minute. (**G**) Relative total area under the curve per ROI during a 5-minute recording from the mice in **E**. Data are expressed as means ± SEM. ***P < 0.001; ****P < 0.0001.

Next, we investigated whether the increased Ca^2+^ activity in BRAF KE–expressing astrocytes is dependent on signaling through the ERK pathway. To this end, we employed a dominant-negative mutation, K97M, in the mitogen-activated protein kinase kinase 1 protein (dnMEK1) (67), the downstream effector of RAF and the upstream activator of ERK. We first confirmed that dnMEK1 successfully blocks BRAF KE–induced overactivation of RAS/ERK signaling in human embryonic kidney (HEK) 293T cells (Supplemental Figure 23). To determine whether dnMEK1 can prevent BRAF KE–induced overactivation of RAS/ERK signaling and the subsequent aberrantly hyperactive Ca^2+^ fluctuations in hippocampal astrocytes, we injected GFAP–BRAF KE or GFAP-BRAF WT virus together with AAV5-GFAP-HA-dnMEK1 (GFAP-dnMEK1) virus or saline into the dorsal CA1 region of the hippocampus. We also injected gfaABC1D-GCaMP6f virus into the same region of the hippocampus of all groups (Figure 5A). Immunoblot analyses demonstrated significantly increased p-ERK1/2 and GFAP protein levels in BRAF KE–expressing hippocampi compared with BRAF WT–expressing hippocampi (Figure 5, B–D). Notably, coexpression of dnMEK1 with BRAF KE significantly inhibited the increase in both p-ERK1/2 and GFAP expression in the hippocampus (Figure 5, B–D). Coexpressing dnMEK1 with BRAF WT in astrocytes did not have noticeable effects on p-ERK1/2 or GFAP protein levels (Figure 5, B–D). Next, we used 2-photon (2P) confocal microscopy to perform Ca^2+^ imaging in hippocampal slices dissected from 4 groups of mice: WT+saline, WT+dnMEK1, KE+saline, and KE+dnMEK1 (Figure 5E and Supplemental Video 1). This revealed that coexpression of dnMEK1 with BRAF KE in astrocytes suppresses the BRAF KE–induced elevation of Ca^2+^ fluctuation within each ROI whereas coexpression of dnMEK1 and BRAF WT showed no significant changes in Ca^2+^ fluctuation (Figure 5, F and G). These results indicate that the overactivation of the astrocytic RAS/ERK signaling pathway due to BRAF KE expression induces augmented astrocytic Ca^2+^ fluctuations.

### Hyperactive calcium fluctuations in Nestin;BRAF^KE/+^ astrocytes are attenuated by GFAP^+^ astrocyte-specific expression of dnMEK1.

**Figure 6:**
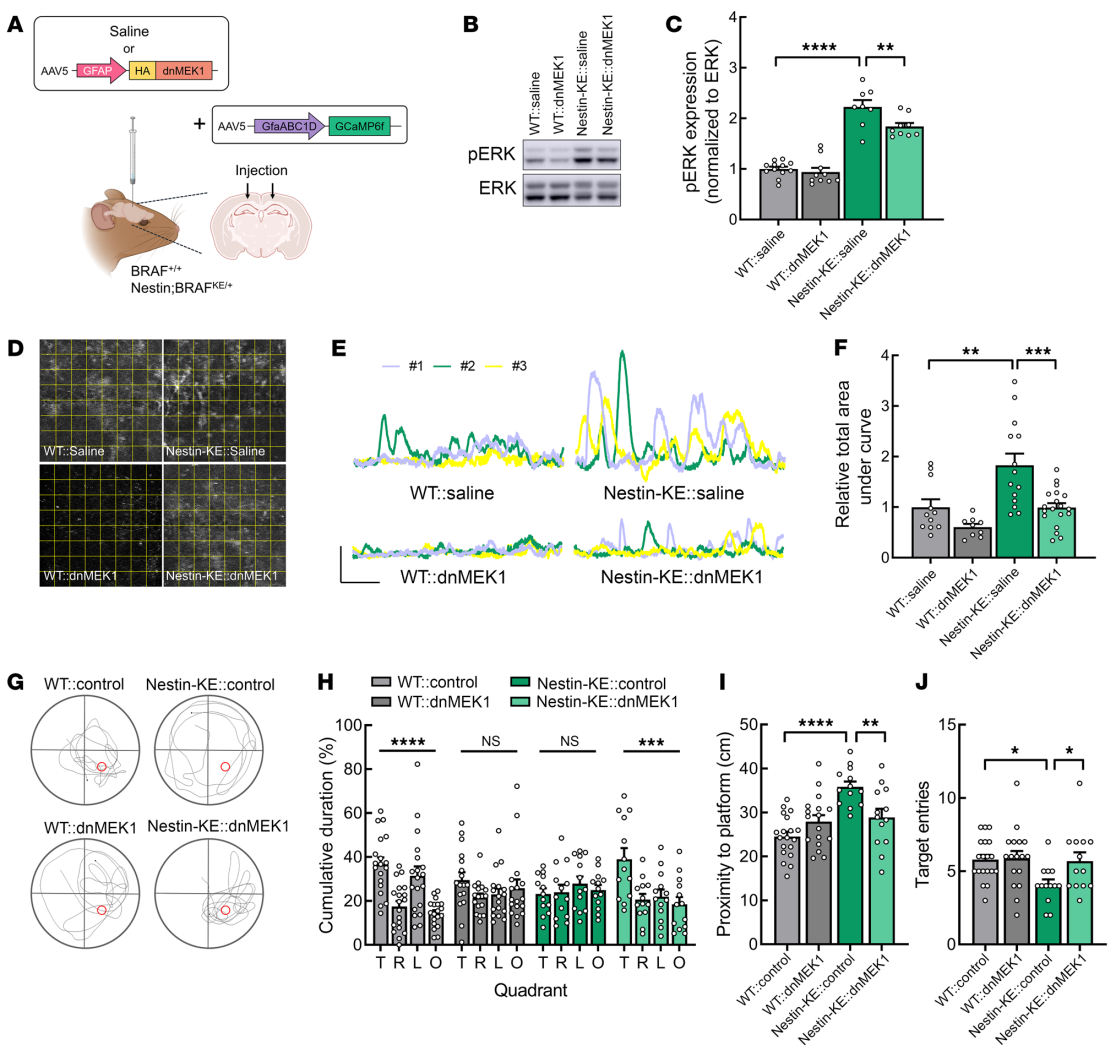
Astrocyte-specific expression of a dominant-negative MEK1 mutant rescues hyperactive astroglial Ca^2+^ fluctuations and hippocampal memory deficits in Nestin;BRAF^KE/+^ mice. (**A**) Schematic illustrating the experimental approach. GFAP-HA-dnMEK1 (dnMEK1) and gfaABC1D-GCaMP6f (GCaMP6f) virus were injected into the hippocampal CA1 region of adult BRAF^+/+^ (WT) and Nestin;BRAF^KE/+^ (Nestin-KE) mice. (**B**) Representative Western blot showing p-ERK1/2 and ERK1/2 expression in hippocampal lysates from BRAF^+/+^ or Nestin;BRAF^KE/+^ mice treated as in **A**. (**C**) Quantification of p-ERK1/2 expression normalized to ERK1/2 expression in samples from **B**. (**D**) Representative images of Ca^2+^ fluctuations measured in hippocampal astrocytes from BRAF^+/+^ and Nestin;BRAF^KE/+^ mice treated as in **A**. The yellow squares indicate each ROI. (**E**) Representative traces from astrocytes in **D**. Scale bars: 20 arbitrary units and 1 minute. (**F**) Relative total area under the curve per ROI during a 5-minute recording from the mice in **E**. (**G**–**J**) For MWM, GFAP-control (mCherry or GFP; control) or GFAP-HA-dnMEK1 (dnMEK1) virus was injected into the hippocampal CA1 region of BRAF^+/+^ (WT) and Nestin;BRAF^KE/+^ (Nestin-KE) mice. (**G**) Representative trajectories of BRAF^+/+^ and Nestin;BRAF^KE/+^ mice injected with GFAP-control or GFAP-HA-dnMEK1 during the MWM probe trial. (**H**) Quadrant occupancy, (**I**) proximity to the platform, or (**J**) number of target zone entries of the mice in **G**. Data are expressed as means ± SEM. *P < 0.05; **P < 0.01; ***P < 0.001; ****P < 0.0001.

The aberrant Ca^2+^ signaling observed in mice overexpressing BRAF KE specifically in astrocytes prompted us to investigate whether Nestin;BRAF^KE/+^ mice, which circumvent overexpression-associated artifacts, also display hyperactive Ca^2+^ fluctuations in astrocytes and whether this could be also attenuated by disrupting ERK signaling. Therefore, we injected GFAP-dnMEK1 virus or saline together with gfaABC1D-GCaMP6f virus into the CA1 region of hippocampus of Nestin;BRAF^KE/+^ mice (Figure 6A). Western blot analyses showed significantly increased p-ERK1/2 protein levels in Nestin;BRAF^KE/+^ hippocampi compared with BRAF^+/+^ hippocampi (Figure 6, B and C). Notably, expression of GFAP-dnMEK1 attenuated p-ERK1/2 levels in hippocampal lysates. Since dnMEK1 is expressed only in astrocytic populations, the effect can be underestimated in Western blot analysis of whole hippocampal tissue (Figure 6, B and C). We then compared astrocyte-specific Ca^2+^ imaging between BRAF^+/+^ and Nestin;BRAF^KE/+^ mice. Nestin;BRAF^KE/+^ mice exhibited significantly increased under the curve area of GCaMP6f fluorescence in each ROI relative to BRAF^+/+^ littermate controls (Figure 6, D–F). This indicates that, similarly to GFAP–BRAF KE virus–injected animals, Nestin;BRAF^KE/+^ knockin mice also exhibit aberrantly hyperactive Ca^2+^ signaling in hippocampal astrocytes. We next examined whether dnMEK1 expression can alleviate hyperactive Ca^2+^ fluctuations in the mutant mice and found that the expression of GFAP-dnMEK1 suppressed astrocyte Ca^2+^ fluctuations augmented in Nestin;BRAF^KE/+^ mice (Figure 6, D–F, and Supplemental Video 2). These data suggest that BRAF KE expression using an endogenous knockin strategy also induces the overactivation of the RAS/ERK pathway in astrocytes, leading to augmented astrocytic Ca^2+^ fluctuations.

### Spatial memory deficits can be rescued by normalizing astrocytic ERK signaling in Nestin;BRAF^KE/+^ mice.

Next, we tested whether attenuating overactivated ERK signaling selectively in astrocytes could also reverse the impaired spatial learning and memory in Nestin;BRAF^KE/+^ mice. We injected AAV5-GFAP-control (mCherry or EGFP; control) or GFAP-dnMEK1 virus into the CA1 region of the hippocampus of BRAF*^+/+^* and Nestin;BRAF^KE/+^ mice. We confirmed that over 98% of the cells labeled by HA-dnMEK1^+^ or mCherry^+^ also expressed GFAP, indicating that the expression of dnMEK1 and its control were highly selective to astrocytes (Supplemental Figure 24). In the MWM training sessions, Nestin;BRAF^KE/+^ mice injected with GFAP-dnMEK1 virus showed a similar latency in reaching the platform as Nestin;BRAF^KE/+^ mice injected with control virus (Supplemental Figure 25). However, in the probe trial, GFAP-dnMEK1–injected Nestin;BRAF^KE/+^ mice spent significantly longer times in the target quadrant than in the other quadrants, exhibiting performance comparable to the BRAF^+/+^ mice injected with control virus (Figure 6, G and H). In contrast, Nestin;BRAF^KE/+^ mice injected with the control virus spent almost equal time in all quadrants (Figure 6, G and H). Moreover, Nestin;BRAF^KE/+^ mice expressing GFAP-dnMEK1 swam closer to the position where the platform had been located during the training sessions and visited the target quadrant more frequently compared with the control group (Figure 6, I and J). Since performance during MWM probe trials is more sensitive to hippocampal function than performance during training trials, performance in MWM probe trials is the more rigorous test of memory performance (68). Taken together, these results demonstrate that learning deficits in Nestin;BRAF^KE/+^ mice can be rescued even in 3- to 4-month-old adult mice by attenuating ERK signaling selectively in astrocytes in the hippocampus.

### Normalizing aberrant Ca^2+^ signaling in BRAF KE–expressing astrocytes restores hippocampal memory.

**Figure 7:**
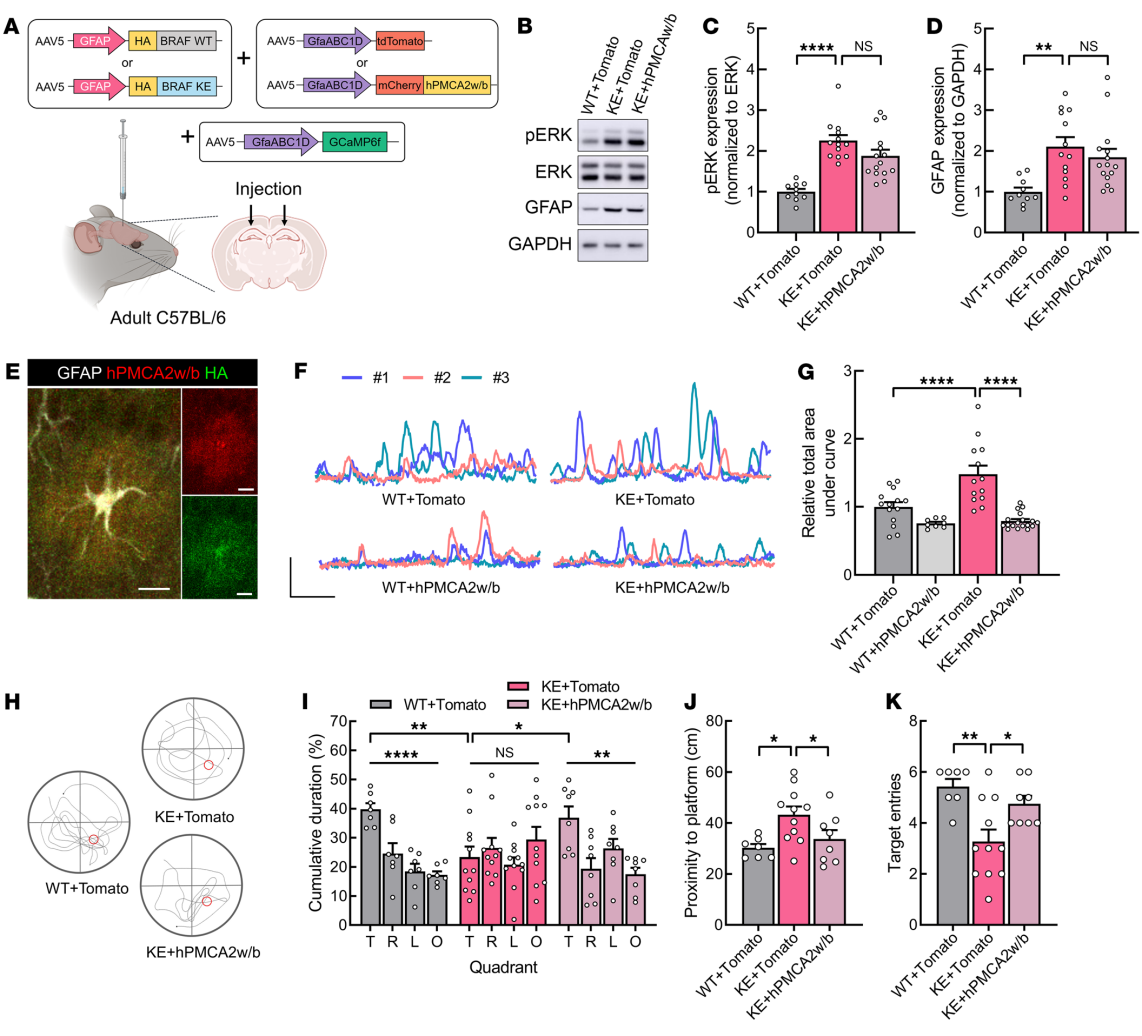
Astrocyte-specific expression of hPMCA2w/b attenuates the hyperactive astroglial Ca^2+^ fluctuations and hippocampal memory deficits of BRAF KE–injected mice. (**A**) Schematic illustrating the experimental approach. AAV-GFAP-HA-BRAF WT (WT) or AAV-GFAP–HA–BRAF KE (KE), gfaABC1D-hPMCA2w/b (hPMCA2w/b) or gfaABC1D-Tomato (Tomato), and AAV-gfaABC1D-GCaMP6f were injected into the hippocampal CA1 region of adult C57BL/6 WT mice. (**B**) Representative Western blot showing p-ERK1/2, ERK1/2, GFAP, and GAPDH expression in hippocampal lysates from the mice injected in **A**. (**C**) p-ERK1/2 expression normalized to ERK1/2 expression or (**D**) GFAP expression normalized to GAPDH expression in the mice injected in **A**. (**E**) Representative images showing colocalization of hPMCA2w/b (red) and HA (green) in GFAP-expressing astrocytes. Scale bars: 10 μm. (**F** and **G**) Ca^2+^ fluctuations in astrocytes of hippocampal slices from BRAF WT or BRAF KE mice injected with Tomato or hPMCA2w/b. (**F**) Representative GCaMP fluorescence traces recorded in astrocytes. Scale bars: 40 arbitrary units and 1 minute. (**G**) Relative total area under the curve per ROI during a 5-minute recording. (**H**–**K**) For the MWM experiments, Tomato or hPMCA2w/b virus was injected into the hippocampal CA1 region of BRAF WT or BRAF KE mice. (**H**) Representative trajectories of WT or KE mice injected with Tomato or hPMCA2w/b in the MWM probe trial. (**I**) Quadrant occupancy, (**J**) proximity to the platform, or (**K**) number of target zone entries of the mice in **H**. Data are expressed as means ± SEM. *P < 0.05; **P < 0.01; ****P < 0.0001.

Based on our finding that the attenuation of hyperactive RAS/ERK signaling in astrocytes alleviated hyperactive astrocytic Ca^2+^ signaling and rescued the spatial learning and memory deficits of Nestin;BRAF^KE/+^ mice, we hypothesized that the BRAF KE mutation induces memory deficits via the sequential dysregulation of astrocyte RAS/ERK signaling and Ca^2+^ signaling. To test this hypothesis, we employed isoforms of the plasma membrane Ca^2+^ pump PMCA (hPMCA2w/b), which constitutively export cytosolic Ca^2+^ (69, 70). We injected GFAP–BRAF KE or WT virus, gfaABC1D-hPMCA2w/b or gfaABC1D-tdTomato virus, and gfaABC1D-GCaMP6f virus into the hippocampal CA1 region of adult mice (Figure 7, A and E). In a Western blot analysis of the resulting hippocampal extracts, we found that astrocyte-specific hPMCA2w/b expression did not reduce the elevated expression of p-ERK1/2 or GFAP in GFAP–BRAF KE–expressing mice. This suggests that hyperactive Ca^2+^ signaling may not be the upstream effector driving the increase in GFAP or p-ERK in BRAF KE astrocytes (Figure 7, B–D). Next, we recorded GCaMP6f fluorescence signals in hippocampal slices and found that mice coexpressing both hPMCA2w/b and BRAF KE showed significantly reduced astrocyte Ca^2+^ fluctuations than mice expressing BRAF KE alone, confirming the functional expression of hPMCA2w/b in vivo (Figure 7, F and G, and Supplemental Video 3). In MWM training trials, mice expressing BRAF KE and hPMCA2w/b (KE+hPMCA2w/b) exhibited a similar latency to reach the platform as mice expressing BRAF KE and a tdTomato control (KE+Tomato) (Supplemental Figure 26). In probe trials, although KE+hPMCA2w/b mice performed similarly to the control group (WT+Tomato), spending significantly more time in the target quadrant, KE+Tomato mice showed no preference for the target quadrant (Figure 7, H and I). KE+hPMCA2w/b mice also swam nearer where the platform had been located in the training trials and entered the target quadrant more frequently than KE+Tomato mice, suggesting hPMCA2w/b rescued the spatial memory deficits induced by BRAF KE (Figure 7, J and K). Together, these results demonstrate that increased astrocyte Ca^2+^ signaling underlies the spatial memory deficits of mice injected with GFAP–BRAF KE virus.

## Discussion

In this study, we demonstrate that the BRAF KE mutation associated with several RASopathies induces hyperactive RAS/ERK signaling that disrupts normal astrocytic cell function, impairing hippocampal learning and memory. Specifically, expression of the BRAF KE mutation in Nestin^+^ neural stem cells, but not αCaMKII^+^ excitatory or vGAT^+^ inhibitory neurons, resulted in hippocampal memory deficits in mice. Nestin;BRAF^KE/+^ mice also showed impaired hippocampal LTD. Further, BRAF KE expression in genetically engineered mouse and human spheroid models induced reactive astrogliosis. Expressing BRAF KE in adult astrocytes led to hypertrophy and hyperactive Ca^2+^ fluctuations that were sufficient to induce hippocampal learning and memory deficits. Notably, reduction of astrocyte-specific ERK signaling and Ca^2+^ activity rescued this hippocampal memory impairment. Collectively, these data indicate that BRAF KE–associated cognitive impairment arises due to dysregulation of astrocytic ERK signaling and a subsequent disruption of Ca^2+^ regulation.

Several mouse models carrying RASopathy-associated BRAF mutations have been characterized. Mice with a C57BL/6 background that are heterozygous for the BRAF Q241R mutation display embryonic lethality due to multiple developmental defects, including cardiac abnormalities and liver necrosis (20). In contrast, BRAF Q241R mice with an ICR/CD-1 background survive to adulthood, yet show growth retardation, craniofacial dysmorphism, and memory impairment in contextual fear conditioning (19). Mice that express BRAF L597V are short in stature and exhibit facial dysmorphia and cardiac defects (21). Consistent with our findings in Nestin;BRAF^KE/+^ mice, mouse models carrying the BRAF V600E mutation showed multiple RASopathy-like phenotypes and also exhibited increased GFAP^+^ cells in the hippocampus and cortex, regardless of their genetic background (18). In contrast to the mature (cultured more than 30 weeks) 3D spheroids expressing BRAF KE in our study, a CFC patient iPSC-derived neurodevelopmental model expressing the BRAF^Q257R/+^ mutation (Q241R in mice) showed significantly reduced astroglial markers, including GFAP and S100β, after 5 weeks of differentiation (25). This may have been a result of the use of 2D culture conditions, rather than the 3D culture conditions we used in this study, or it could have been due to different levels of differentiation. In addition, neural progenitor cells expressing BRAF^Q257R/+^ and mouse embryos expressing BRAF^Q241R/+^ showed reduced AKT phosphorylation, suggesting engagement with multiple pathways, including PI3K/AKT signaling (19, 20, 25). In our experiments, however, we did not observe any significant change in PI3K/AKT signaling, as assessed by the phosphorylation of S6 in the hippocampus of Nestin;BRAF^KE/+^ mice. This, along with the phenotypic differences of mouse models with different genetic backgrounds, suggests RASopathy-associated *BRAF* mutations exert heterogenous effects on downstream signaling pathways depending on the way each mutation interacts with each genetic background throughout development.

Interestingly, we observed a significant increase in reactive GFAP^+^ astrocytes in Nestin;BRAF^KE/+^ mice around postnatal day 40 (Supplemental Figure 16). In Nestin;BRAF^KE/+^ mice, the time course of mutant BRAF expression depends on 2 factors. First, it depends on Cre expression because the loxP sites must be removed by Cre-dependent recombination. Second, it also follows the time course of endogenous *Braf* gene expression because the knocked in gene is still under the control of the endogenous *Braf* promoter (Supplemental Figure 1). Since the Nestin promoter begins to drive expression of Cre recombinase before embryonic day 12 (71), we can assume that the knockin process begins at the same time. Interestingly, BRAF expression rises dramatically in cortical and hippocampal astrocytes between postnatal day 32 and 10 weeks of age (72, 73). Thus, it is reasonable to think that the increased expression of GFAP in Nestin;BRAF^KE/+^ mice at day 40 is faithfully following the process of development rather than being affected by the genetic knockin process. We would like to note that the mutant BRAF expression driven by the αCaMKII-Cre line occurs later in postnatal development than that driven by the Nestin-Cre or vGAT-Cre lines (74, 75). We cannot, therefore, rule out the possibility that we missed a potential impact of BRAF KE on excitatory neurons during critical early stages of circuit formation. Future studies may be able to overcome this caveat by incorporating the use of other excitatory neuron-specific Cre lines (e.g., vGlut-Cre) expressed earlier in development.

We found that expressing BRAF KE in CaMKII^+^ excitatory or vGAT^+^ inhibitory neurons did not induce learning deficits. This is unsurprising because the effect of enhanced neuronal ERK activity on learning in mouse RASopathy models is inconsistent across the literature. RAS/ERK hyperactivity in either excitatory or inhibitory neurons can impair hippocampal learning in mouse RASopathy models (3, 10, 11, 14), but this is inconsistent. For example, transgenic mice expressing the gain-of-function HRAS G12V mutation under the control of the αCaMKII promoter showed enhanced hippocampal learning and memory (76). Heterozygous mice expressing the gain-of-function RAF1 L613V mutation also demonstrated enhanced learning in multiple behavioral tasks, despite displaying increased brain glial cells (24). While the underlying mechanisms remain unexplored, it is possible that these diverse behavioral outcomes are caused by differences in the temporal and spatial specificity with which each mutation activates RAS/ERK signaling (3).

Nevertheless, our results in diverse models, including several mutant mouse lines, human cortical spheroids, and primary astrocyte cultures, provide strong evidence that BRAF KE induces ERK hyperactivity in astrocytes, subsequently impairing hippocampal memory in mice. This is consistent with a crucial role for astrocytes in cognition and in the pathophysiology of various neurodegenerative and neurodevelopmental diseases (32, 34, 61, 77, 78). Several studies reported a role for RAS/ERK signaling in modulating astrocyte differentiation. For example, gain-of-function mutations in *Mek1* or *Raf1* or deletion of *Nf1* can increase glial cells in the mouse cortex and hippocampus (24, 27–29). But how RAS/ERK signaling regulates astrocyte physiology in the adult brain remains poorly understood. We found that astrocyte-specific expression of BRAF KE increases spontaneous Ca^2+^ fluctuations in astrocytes that can be normalized via mitogen-activated protein kinase kinase (MEK) suppression. Consistent with this, a previous study found that increased ERK activity in astrocytes in the presence of growth factors enlarged intracellular Ca^2+^ stores, increased oscillatory astrocytic Ca^2+^ responses, promoted proliferation, and induced a hypertrophic morphology in astrocytes stimulated with glutamate or adenosine triphosphate (ATP) (79). Our transcriptomic analyses indicated marked changes in genes regulating Ca^2+^ homeostasis, including *Cacna1g*, *Atp2a2*, and *Grik2* in the Nestin;BRAF^KE/+^ hippocampus and *SLC8A1*, *GRIA2*, *GRM3*, *EDNRB*, *RYR2*, *ITPR2*, *ORI1*, *ORAI3*, *GRIK5*, and *GRIK4* in BRAF KE spheroids. This suggests the possibility that these molecular changes underlie the altered Ca^2+^ homeostasis induced by the BRAF KE mutation. It is still unclear how BRAF/ERK signaling regulates these genes and whether the BRAF KE mutation increases spontaneous Ca^2+^ fluctuations in the cytoplasm (e.g., via target protein phosphorylation) or in the nucleus via transcriptional regulation. Nevertheless, we found that the expression of dnMEK1 reverses Ca^2+^ fluctuations in both AAV-injected mice and Nestin;BRAF^KE/+^ mice, suggesting that astrocytic Ca^2+^ fluctuations are somehow modulated by the RAS/ERK pathway. Furthermore, expression of dnMEK1 or hPMCA2w/b restored Ca^2+^ fluctuations in BRAF KE–expressing mice, suggesting that the reversal of functional features such as Ca^2+^ fluctuations in astrocytes is sufficient to rescue the behavioral deficits of adult mice. It is still unclear whether other RASopathy-associated mutations also affect behavior by modulating fluctuations in astrocytic Ca^2+^. The astrocyte-specific deletion of *Nf1*, however, did not impair spatial memory in mice, suggesting that only specific RASopathy genes are critical for astrocytic function (11).

Disruptions in astrocyte Ca^2+^ dynamics may alter gliotransmitter release and affect various aspects of neural activity, synaptic plasticity, and behavior (32, 80). For example, in a mouse model of Alzheimer’s disease (AD), cortical astrocytes exhibit a drastic reduction in Ca^2+^ signaling that accompanies memory loss at early AD stages (81). In Huntington’s disease, astrocytes display hyperactive Ca^2+^-dependent glutamate release, leading to an accumulation of extracellular glutamate that then causes overpotentiation of neuronal activity and cytotoxicity (82, 83). Notably, astrocytic Ca^2+^ activity has also been implicated in the expression of hippocampal NMDAR-LTD (84–86). Optogenetic activation of astrocytes was sufficient to induce LTD in the absence of presynaptic activity, and interfering with astrocytic Ca^2+^ signaling strongly reduced LTD in the hippocampus without affecting LTP (84). These results, revealing the involvement of astrocytes in hippocampal NMDAR-LTD, are consistent with our findings. Since astrocytic Ca^2+^ activity and the consequent vesicular release of gliotransmitters are required for LTD, it is reasonable to speculate that chronically elevated Ca^2+^ signaling in BRAF KE–expressing astrocytes may disrupt proper astrocytic Ca^2+^ dynamics and astrocyte-neuron communication, resulting in the LTD deficit. The mechanisms underlying LTD impairment, including the identity of altered gliotransmitters in the mutant mice, however, will require further study.

Together, our results demonstrate that cognitive impairments linked to the BRAF KE mutation are mediated by astrocytic dysfunction — more specifically, an aberrant increase in Ca^2+^ signaling in mature astrocytes. Moreover, we show that reducing ERK activity or Ca^2+^ signaling selectively in astrocytes is sufficient to improve memory in RASopathy model mice. Thus, astrocytes may be a promising therapeutic target for treating the cognitive symptoms of RASopathy patients.

## Methods

Additional details can be found in Supplemental Methods.

### Sex as a biological variable.

Our study examined male and female animals, and similar findings are reported for both sexes. Sex information is indicated in Supporting Data Values.

### Statistics.

All results are presented as the mean ± SEM. All statistical tests were performed using a 2-tailed *t* test, 1-way ANOVA, or 2-way ANOVA. Detailed statistical information is provided in Supplemental Table 4. All statistics had a significance cutoff of *P* value less than 0.05 and were calculated using Prism software, version 9.5 (GraphPad Software).

### Study approval.

Animal protocols were approved by the Seoul National University Institutional Animal Care and Use Committee (SNU-220107-2-1). The human study was approved by the SNU Hospital Institutional Review Board (approval number: 2204.112.1317), and Severance Hospital Institutional Review Board (approval number: 4-2013-0096). All experiments were performed in accordance with the institutional guidelines and regulations.

### Data availability.

All materials related to this study, including BRAF^K499E^ ^floxed/+^ mice produced for this study, are available upon reasonable request. RNA-Seq data were deposited to the NCBI’s Gene Expression Omnibus Series (GEO GSE234764 for mouse and human spheroid transcriptome; GSE283653 for astrocyte transcriptome. Reagents including viral vectors will be made freely available to academic laboratories. Values for all data points in graphs are reported in the Supporting Data Values file.

## Author contributions

YSL, MK, and CHK conceived the project, designed experiments, and wrote the manuscript. MK performed behavioral and histological experiments, stereotaxic surgeries, plasmid construction, biochemical experiments, and Ca^2+^ imaging experiments with the help of MY, Soobin Kim, JL, and KK. MK, HHR, HJ, Sun Yong Kim, KDW, MGK, PP, JEC, and DHH performed electrophysiological experiments. JC generated gene-edited iPSC lines, a patient-derived iPSC line, and human cortical spheroids and performed histological experiments. MK and JC contributed equally as co–first authors, with MK listed first for conducting the majority of the work, including initiating the study and leading animal experiments, while JC focused on human iPSC experiments and data analysis. JMK saw and diagnosed a RASopathy patient. KAC generated a control iPSC line. JH performed primary astrocyte culture and ICC. BGN and TA generated BRAF^K499E^ ^floxed/+^ mice. SWK, Seoyeon Kim, YK, and JYA analyzed the RNA-Seq data. JK, SC, BKK, SJK, and HP analyzed data. All authors edited the manuscript.

## Supplementary Material

Supplemental data

Unedited blot and gel images

Supplemental table 1

Supplemental table 2

Supplemental table 3

Supplemental table 4

Supplemental video 1

Supplemental video 2

Supplemental video 3

Supporting data values

## Version 1 02/18/2025

In-Press Preview

## Version 2 04/15/2025

Electronic publication

## References

[1] Kolch W (2000). Meaningful relationships: the regulation of the Ras/Raf/MEK/ERK pathway by protein interactions. Biochem J.

[2] Boguski MS, McCormick F (1993). Proteins regulating Ras and its relatives. Nature.

[3] Ryu HH, Lee YS (2016). Cell type-specific roles of RAS-MAPK signaling in learning and memory: implications in neurodevelopmental disorders. Neurobiol Learn Mem.

[4] Shilyansky C (2010). Molecular and cellular mechanisms of learning disabilities: a focus on NF1. Annu Rev Neurosci.

[5] Kim YE, Baek ST (2019). Neurodevelopmental aspects of RASopathies. Mol Cells.

[6] Rauen KA (2013). The RASopathies. Annu Rev Genomics Hum Genet.

[7] Tidyman WE, Rauen KA (2009). The RASopathies: developmental syndromes of Ras/MAPK pathway dysregulation. Curr Opin Genet Dev.

[8] Kang M, Lee YS (2019). The impact of RASopathy-associated mutations on CNS development in mice and humans. Mol Brain.

[9] Jindal GA (2015). RASopathies: unraveling mechanisms with animal models. Dis Model Mech.

[10] Ryu HH (2019). Excitatory neuron-specific SHP2-ERK signaling network regulates synaptic plasticity and memory. Sci Signal.

[11] Cui Y (2008). Neurofibromin regulation of ERK signaling modulates GABA release and learning. Cell.

[12] Costa RM (2002). Mechanism for the learning deficits in a mouse model of neurofibromatosis type 1. Nature.

[13] Papale A (2017). Severe intellectual disability and enhanced gamma-aminobutyric acidergic synaptogenesis in a novel model of rare RASopathies. Biol Psychiatry.

[14] Ryu HH (2020). Neuron type-specific expression of a mutant KRAS impairs hippocampal-dependent learning and memory. Sci Rep.

[15] Niihori T (2006). Germline KRAS and BRAF mutations in cardio-facio-cutaneous syndrome. Nat Genet.

[16] Yoon G (2007). Neurological complications of cardio-facio-cutaneous syndrome. Dev Med Child Neurol.

[17] Lee Y (2021). Clinical and molecular spectra of BRAF-associated RASopathy. J Hum Genet.

[18] Urosevic J (2011). Constitutive activation of B-Raf in the mouse germ line provides a model for human cardio-facio-cutaneous syndrome. Proc Natl Acad Sci U S A.

[19] Moriya M (2015). Adult mice expressing a Braf Q241R mutation on an ICR/CD-1 background exhibit a cardio-facio-cutaneous syndrome phenotype. Hum Mol Genet.

[20] Inoue S (2014). New BRAF knockin mice provide a pathogenetic mechanism of developmental defects and a therapeutic approach in cardio-facio-cutaneous syndrome. Hum Mol Genet.

[21] Andreadi C (2012). The intermediate-activity (L597V)BRAF mutant acts as an epistatic modifier of oncogenic RAS by enhancing signaling through the RAF/MEK/ERK pathway. Genes Dev.

[22] Chen AP (2006). Forebrain-specific knockout of B-raf kinase leads to deficits in hippocampal long-term potentiation, learning, and memory. J Neurosci Res.

[23] Pfeiffer V (2013). Ablation of BRaf impairs neuronal differentiation in the postnatal hippocampus and cerebellum. PLoS One.

[24] Holter MC (2019). The Noonan syndrome-linked Raf1L613V mutation drives increased glial number in the mouse cortex and enhanced learning. PLoS Genet.

[25] Yeh E (2017). Patient-derived iPSCs show premature neural differentiation and neuron type-specific phenotypes relevant to neurodevelopment. Mol Psychiatry.

[26] Krencik R (2015). Dysregulation of astrocyte extracellular signaling in Costello syndrome. Sci Transl Med.

[27] Aoidi R (2018). *Mek1*^Y130C^ mice recapitulate aspects of human cardio-facio-cutaneous syndrome. Dis Model Mech.

[28] Wang Y (2012). ERK inhibition rescues defects in fate specification of Nf1-deficient neural progenitors and brain abnormalities. Cell.

[29] Zhu Y (2005). Inactivation of NF1 in CNS causes increased glial progenitor proliferation and optic glioma formation. Development.

[30] Zhu Y (2001). Ablation of NF1 function in neurons induces abnormal development of cerebral cortex and reactive gliosis in the brain. Genes Dev.

[31] Paquin A (2009). Costello syndrome H-Ras alleles regulate cortical development. Dev Biol.

[32] Jo S (2014). GABA from reactive astrocytes impairs memory in mouse models of Alzheimer’s disease. Nat Med.

[33] Wheeler MA (2020). MAFG-driven astrocytes promote CNS inflammation. Nature.

[34] Wang Q (2021). Impaired calcium signaling in astrocytes modulates autism spectrum disorder-like behaviors in mice. Nat Commun.

[35] Pierpont EI (2010). Effects of germline mutations in the Ras/MAPK signaling pathway on adaptive behavior: cardiofaciocutaneous syndrome and Noonan syndrome. Am J Med Genet A.

[36] Rodriguez-Viciana P (2006). Germline mutations in genes within the MAPK pathway cause cardio-facio-cutaneous syndrome. Science.

[37] Lim CS (2017). BRaf signaling principles unveiled by large-scale human mutation analysis with a rapid lentivirus-based gene replacement method. Genes Dev.

[38] Schulz AL (2008). Mutation and phenotypic spectrum in patients with cardio-facio-cutaneous and Costello syndrome. Clin Genet.

[39] Roberts A (2006). The cardiofaciocutaneous syndrome. J Med Genet.

[40] Adviento B (2014). Autism traits in the RASopathies. J Med Genet.

[41] Suzuki S (2010). The neural stem/progenitor cell marker nestin is expressed in proliferative endothelial cells, but not in mature vasculature. J Histochem Cytochem.

[42] Papadopoulou E (2011). CNS imaging is a key diagnostic tool in the evaluation of patients with CFC syndrome: two cases and literature review. Am J Med Genet A.

[43] Pierpont ME (2014). Cardio-facio-cutaneous syndrome: clinical features, diagnosis, and management guidelines. Pediatrics.

[44] Seth S (2016). Cardiofaciocutaneous syndrome: case report of a rare disorder. J Clin Diagn Res.

[45] Kavamura MI (2002). CFC index for the diagnosis of cardiofaciocutaneous syndrome. Am J Med Genet.

[46] Giusti SA (2014). Behavioral phenotyping of Nestin-Cre mice: implications for genetic mouse models of psychiatric disorders. J Psychiatr Res.

[47] Lee YS, Silva AJ (2009). The molecular and cellular biology of enhanced cognition. Nat Rev Neurosci.

[48] Lee YS (2014). Mechanism and treatment for learning and memory deficits in mouse models of Noonan syndrome. Nat Neurosci.

[49] Cesarini L (2009). Cognitive profile of disorders associated with dysregulation of the RAS/MAPK signaling cascade. Am J Med Genet A.

[50] Pierpont EI (2009). Genotype differences in cognitive functioning in Noonan syndrome. Genes Brain Behav.

[51] Kobayashi I (1986). Noonan’s syndrome with syringomyelia. Jpn J Psychiatry Neurol.

[52] Reynolds JF (1986). New multiple congenital anomalies/mental retardation syndrome with cardio-facio-cutaneous involvement--the CFC syndrome. Am J Med Genet.

[53] Chrzanowska K (1989). Cardio-facio-cutaneous (CFC) syndrome: report of a new patient. Am J Med Genet.

[54] Gutmann DH (1999). Haploinsufficiency for the neurofibromatosis 1 (NF1) tumor suppressor results in increased astrocyte proliferation. Oncogene.

[55] Angara K (2020). Nf1 deletion results in depletion of the Lhx6 transcription factor and a specific loss of parvalbumin^+^ cortical interneurons. Proc Natl Acad Sci U S A.

[56] Holter MC (2021). Hyperactive MEK1 signaling in cortical GABAergic neurons promotes embryonic parvalbumin neuron loss and defects in behavioral inhibition. Cereb Cortex.

[57] Escartin C (2021). Reactive astrocyte nomenclature, definitions, and future directions. Nat Neurosci.

[58] Hol EM, Pekny M (2015). Glial fibrillary acidic protein (GFAP) and the astrocyte intermediate filament system in diseases of the central nervous system. Curr Opin Cell Biol.

[59] Michetti F (2019). The S100B story: from biomarker to active factor in neural injury. J Neurochem.

[60] Liu LR (2020). Interaction of microglia and astrocytes in the neurovascular unit. Front Immunol.

[61] Chun H (2020). Severe reactive astrocytes precipitate pathological hallmarks of Alzheimer’s disease via H_2_O_2_^–^ production. Nat Neurosci.

[62] Ehrman LA (2014). The protein tyrosine phosphatase Shp2 is required for the generation of oligodendrocyte progenitor cells and myelination in the mouse telencephalon. J Neurosci.

[63] Paşca AM (2015). Functional cortical neurons and astrocytes from human pluripotent stem cells in 3D culture. Nat Methods.

[64] Ran FA (2013). Genome engineering using the CRISPR-Cas9 system. Nat Protoc.

[65] Guerra-Gomes S (2017). Functional roles of astrocyte calcium elevations: from synapses to behavior. Front Cell Neurosci.

[66] Tanaka M (2013). Astrocytic Ca2+ signals are required for the functional integrity of tripartite synapses. Mol Brain.

[67] Mansour SJ (1994). Transformation of mammalian cells by constitutively active MAP kinase kinase. Science.

[68] Maei HR (2009). What is the most sensitive measure of water maze probe test performance?. Front Integr Neurosci.

[69] Strehler EE (2015). Plasma membrane calcium ATPases: from generic Ca(2+) sump pumps to versatile systems for fine-tuning cellular Ca(2.). Biochem Biophys Res Commun.

[70] Yu X (2018). Reducing astrocyte calcium signaling in vivo alters striatal microcircuits and causes repetitive behavior. Neuron.

[71] Zimmerman L (1994). Independent regulatory elements in the nestin gene direct transgene expression to neural stem cells or muscle precursors. Neuron.

[72] Clarke LE (2018). Normal aging induces A1-like astrocyte reactivity. Proc Natl Acad Sci U S A.

[73] Hamed AA (2022). A brain precursor atlas reveals the acquisition of developmental-like states in adult cerebral tumours. Nat Commun.

[74] Tsien JZ (1996). Subregion- and cell type-restricted gene knockout in mouse brain. Cell.

[75] Saito K (2010). The physiological roles of vesicular GABA transporter during embryonic development: a study using knockout mice. Mol Brain.

[76] Kushner SA (2005). Modulation of presynaptic plasticity and learning by the H-ras/extracellular signal-regulated kinase/synapsin I signaling pathway. J Neurosci.

[77] Scuderi C (2014). Sirtuin modulators control reactive gliosis in an in vitro model of Alzheimer’s disease. Front Pharmacol.

[78] Allen M (2022). Astrocytes derived from ASD individuals alter behavior and destabilize neuronal activity through aberrant Ca^2+^ signaling. Mol Psychiatry.

[79] Morita M (2003). Dual regulation of calcium oscillation in astrocytes by growth factors and pro-inflammatory cytokines via the mitogen-activated protein kinase cascade. J Neurosci.

[80] Wu Z (2014). Tonic inhibition in dentate gyrus impairs long-term potentiation and memory in an Alzheimer’s [corrected] disease model. Nat Commun.

[81] Lia A (2023). Rescue of astrocyte activity by the calcium sensor STIM1 restores long-term synaptic plasticity in female mice modelling Alzheimer’s disease. Nat Commun.

[82] Lee W (2013). Enhanced Ca(2+)-dependent glutamate release from astrocytes of the BACHD Huntington’s disease mouse model. Neurobiol Dis.

[83] Tong X (2014). Astrocyte Kir4.1 ion channel deficits contribute to neuronal dysfunction in Huntington’s disease model mice. Nat Neurosci.

[84] Navarrete M (2019). Astrocytic p38α MAPK drives NMDA receptor-dependent long-term depression and modulates long-term memory. Nat Commun.

[85] Pérez-Rodríguez M (2019). Adenosine receptor-mediated developmental loss of spike timing-dependent depression in the hippocampus. Cereb Cortex.

[86] Falcón-Moya R (2020). Astrocyte-mediated switch in spike timing-dependent plasticity during hippocampal development. Nat Commun.

