# Supplementary material for: Aberrant ERK signaling in astrocytes impairs learning and memory in RASopathy-associated BRAF mutant mouse models
Source: J Clin Invest. 2025 Feb 18;135(8):e176631. doi: 10.1172/JCI176631 (PMC11996877; doi:10.1172/JCI176631)
Supplement: Supplemental data [file jci-135-176631-s123.pdf]

Supplemental information

Aberrant ERK signaling in astrocytes impairs learning and memory in RASopathy-associated *BRAF* mutant mouse models

Supplemental methods & reference

26 Supplemental figures & legends

Titles and captions for 4 supplemental tables (separately uploaded)

Legends for 3 supplemental videos (separately uploaded)

Supplemental methods

Generation of inducible BRAF^{K499E} knock-in mice

To construct the targeting vector for our inducible BRAF K499E knock-in mice, a short arm containing Braf exon 9 and a long arm including exons 10–12 were ligated into the vector pGK Neo-HSV-1 TK. The K499E (exon 11) mutation, marked by a unique BstBI site, was introduced by site-directed mutagenesis. A splice acceptor sequence, a Braf cDNA fragment encoding wild-type exons 10–22, and a pGK-Neo (Neo) gene were positioned after the first loxP site. The targeting vector was linearized with SacII and electroporated into G4 ES cells (129S6 and C57BL/6 F1 background). Genomic DNA, isolated from doubly G418/1-resistant and (2-deoxy-2-fluoro-β-D-arabinofuranosyl)-5 iodouracil–resistant (FIAU-resistant) ES clones (positive and negative selection, respectively), was screened by PCR using primers outside and inside the targeting vector. Homologous recombinants were confirmed by Southern blotting using Neo probes. To further confirm and validate the desired properties of the targeted locus, ES cells were transfected with a Cre-expressing plasmid (MSCV-GFP-Cre) to excise the cDNA-Neo cassette. Expression of BRAF K499E mRNA was confirmed by RT-PCR, followed by digestion with BstBI, which marks the K499E allele. Chimeras were generated by outbred morula aggregation (Toronto Centre of Phenogenomics), and germline transmission was obtained. *The heterozygous A-to-G mutation in each knock-in line was confirmed by using Sanger sequencing.* The presence of the heterozygous K499E mutation (A to G) was confirmed by Sanger sequencing. In addition, because the A-to-G mutation generates a novel BstBI restriction site (TTCGAA), mutant BRAF cDNAs were digested with BstBI to confirm the Cre-dependent recombination and resulting replacement of wild-type exon 11 with one containing the mutation. BRAF primer sequences were: Forward 5'-

CGGAGGACAGAAGTCGGATG-3' and Reverse 5'-GCCGGTCATCAGTTCGTACA-3'. The numbering of K499E is human BRAF, and K484E is the relevant site for mouse BRAF according to the alignment between human and mouse BRAF (Gene ID, 109880; XM_036165749.1 and XP_036021642.1).

*Animals***.**

BRAF$^{K499E\ floxed/+}$ mice were crossed to αCaMKII-Cre (JAX 005359), vGAT-IRES-Cre (JAX 016962), or Nestin-Cre (JAX 003771) mice. Three to six-month-old male and female mutant mice were used. For AAV experiments, three to four-month-old male C57BL/6N mice were used. All experiments used littermates as control group and were carried out and analyzed with the experimenters blinded to genotype. Animals were group housed (two to five mice per cage) on a 12-hour light-dark cycle in the vivarium at Seoul National University (SNU). All studies were approved by the Animal Research Committee at SNU.

Morris water maze (MWM) test.

All the behavior tests were done in adult mice (7 to 11 weeks old), and both male and female mice were used. The MWM test was performed as previously described (1). Briefly, mice were handled for 2 minutes at the same time of each day for 7 consecutive days before the test. The maze consisted of a gray opaque cylindrical tank (diameter, 120 cm) in a room with multiple visual cues, which are the animal’s navigational references for locating the platform. The tank was filled with water (20 °C to 22 °C) and painted white. The tank was divided into four invisible quadrants (target quadrant, opposite quadrant, right quadrant, left quadrant) and a

platform, which was submerged 1 cm under the surface of the water, was placed at the center of the target quadrant. Before the initial trial on day 1, each mouse was placed onto the platform for 30 seconds. On training days, mice were released at the edge of the tank facing the inner wall, and start position was chosen randomly for each trial. When mice failed to reach the platform within 60 seconds of trial, they were gently guided onto the platform and rescued from the maze after 10 seconds. When mice successfully reached the platform, they were rescued from the maze after 10 seconds. Mice were trained with six trials per day 4 consecutive days, and interval between trial 3 and 4 was 45 minutes to 1 hour. In probe trial, platform was absent, and mice were tracked for 60 seconds. Movement was analyzed with tracking software (EthoVision 11.5; Nodulus). The visible platform-version of MWM was performed after the hidden platform test. The platform was tagged with a visible flag, and latency to reach the platform was assessed. Experimenters were blinded to the genotype of mice or type of injected viral vectors.

Object-place recognition (OPR) test.

OPR test was performed as described previously (1). Mice were handled for 5 min for four consecutive days and habituated in a cube-shaped opaque acrylic box (32 cm by 32 cm by 32 cm) for 15 minutes for another 2 days before performing the training and test. In the training session, mice were placed in the arena containing two identical 50 ml glass bottles and were allowed to explore the objects for 10 minutes. In the test session, 24 hours after training, mice were placed in the same arena containing one object that stayed in the same location and the other object relocated to a new position. All locations for the objects were counterbalanced among groups, and objects and arena were cleaned between trials. Sessions were recorded and

later analyzed manually. Experimenters were blinded to the genotype of mice.

Open field test.

Open field test was performed as described previously (2). A cube-shaped acrylic box (32 cm by 32 cm by 32 cm) was used as an arena. After a short acclimation, each mouse was released into the box and allowed to freely explore the arena for 15 min. Movement of mice were analyzed with tracking software (EthoVision; Nodulus).

Three-chamber social test.

Mice were habituated to the three-chamber apparatus for 15 minutes for 2 consecutive days. When the habituations were completed, the mice were released to the center chamber of apparatus which containing a wired cup with stranger mouse and an empty cup. For each set of experiment, the location of two cups was counterbalanced. The movement of mice was tracked for tracking software (EthoVision; Nodulus).

Electrophysiology.

Extracellular recordings of field excitatory postsynaptic potentials (fEPSPs) were performed as previously described (3). Slices from 13-week-old mice were used for long-term potentiation (LTP) recording, and 4- to 5-week-old mice were used for long-term depression (LTD) recording. Sagittal brain slices (400 μm) were prepared with a vibratome (Campden, 7000 smz-2) in ice-cold artificial cerebrospinal fluid (ACSF; 3.5 mM KCl, 1.25 mM NaH_2PO_4, 1.3 mM

$MgSO_4$, 120 mM NaCl, 2.5 mM $CaCl_2$, 10 mM glucose, 20 mM $NaHCO_3$) for LTP recording, or in ice-cold cutting solution (2.5 mM KCl, 1.25 mM NaH_2PO_4, 7 mM $MgCl_2$, 75 mM NaCl, 25 mM glucose, 26 mM $NaHCO_3$, and 75 mM sucrose) for LTD recording. Slices were recovered at room temperature for at least 60 min before recording in ACSF saturated with 95% O_2 and 5% CO_2, and recording was performed in a submerged chamber perfused with ACSF. fEPSP were recorded with platinum-iridium electrode placed in the CA1 stratum radiatum. Bipolar platinum stimulating electrodes were placed in Schaffer-collaterals. Baseline responses were measured with stimulation at an intensity that evoked a response that was approximately 40% in LTP recording and 50-60% in LTD recording of the maximum evoked response. LTP was induced with high frequency stimulation (HFS; one burst consisting of a hundred stimuli at 100 Hz), and LTD was induced with low frequency stimulation (LFS; one burst consisting of nine hundred stimuli at 1 Hz). Data were analyzed with WinLTP software (WinLTP Ltd., Bristol, UK).

Magnetic resonance imaging (MRI).

The 10 weeks old male mice were used. Prior to data acquisition, animals were anesthetized with isoflurane (1.5% in oxygen) and placed inside the magnet in the prone position. The respiration rate and body temperature of the animals were monitored during MRI scan. All MRI data were collected on a 9.4T animal MR scanner with a single channel surface coil (20 mm in diameter) for both RF transmission and signal reception (Agilent Technologies, Santa Clara, CA, USA). The 1st- and 2nd-order auto-shimming was performed followed by manual refinement. After acquiring scout images using a gradient echo sequence, axial and coronal brain images were acquired using a two dimensional T2-weighted fast spin echo sequence with

the following sequence parameters: repetition time (TR) = 3000 ms, effective echo time (TE) = 48 ms, flip angle = 90°/180°, echo train length = 4, field-of-view (FOV) = 25 x 25 mm^2, matrix size = 256 x 256, slice thickness = 0.5 mm, number of slices (no gap) = 35 (axial) and 20 (coronal), receiver bandwidth (BW) = 391 Hz/pixel, and number of signal averages (NSA) = 3.

Tissue preparation for immunohistochemistry (mouse).

Mice were fully anesthetized and transcardially perfused with cold 4% paraformaldehyde and PBS. Then brains were dissected, post-fixed with 4% PFA overnight, dehydrated with 30% sucrose for following 2 days, and sectioned on a cryotome. Tissue sections (20 μm thick) were collected in cryoprotectant solution (50% 0.1 M sodium phosphate buffer, 25% ethylene glycol, and 25% glycerol), and stored at -20 °C.

Immunohistochemistry (IHC)

Tissue sections (20 μm thick) were incubated in a blocking solution consisting of Normal goat serum (or donkey serum) and Triton X-100 in PBS. Sections were then incubated with primary antibody in blocking solution and incubated with tissue sections at 4 °C for 24 to 48 hours followed by incubation with secondary antibodies for 2 to 4 hours at room temperature. Images were acquired on the FV-3000 confocal microscope (Olympus, Japan) with FV31S software. Primary antibodies included: anti-GFAP rabbit IgG (Abcam, ab7260); anti-GFAP mouse IgG (Cell signaling, #3670); anti-NeuN mouse IgG (Millipore, MAB377); anti-S100β rabbit IgG (Abcam, ab52642); anti-Iba1 rabbit IgG (WAKO); anti-Ki67 rabbit IgG (Invitrogen, MA5-

14520); anti-PV mouse IgG (Millipore, MAB1572); anti-SOM rat IgG (Millipore, MAB354); anti-Olig2 rabbit IgG (Millipore, AB9610); anti-Sox2 mouse IgG (Santa cruz, SC-365823); anti-GABA guinea pig IgG (Millipore, AB175); anti-HA rat IgG (Roche, 11 867423001; anti-Flag mouse IgG (Sigma, F1804); anti-pERK rabbit IgG (Cell signaling, 4370).

Sholl analysis.

Sholl analysis was performed on serially stacked confocal images as previously described (4). Images of hippocampal sections immunostained with GFAP antibody were used for Sholl analysis. The Sholl analysis plugin was applied in ImageJ (National Institutes of Health). The serial concentric circles were automatically drawn at 1 μm intervals from the center of the soma to the end of the most distant process in each single astrocyte. Number of intersections and sum of process intersections were analyzed.

Western blotting.

Cortices and hippocampi were dissected and lysed in protein lysis buffer (10 mM Tris-HCL, 1.6% SDS) containing phosphatase inhibitor cocktails and protease (Sigma). Supernatants were collected and protein concentration was measured using a BCA assay kit (Thermo). Equal amounts of proteins (5-10 μg) were loaded on a SDS-PAGE gel, and transferred to nitrocellulose membranes. After blocking with 5% skim milk in TBST solution (Tris-buffer saline containing 0.1% Tween-20) for 40 minutes at room temperature. Then membranes were hybridized with each primary antibody [anti-GRIA1 mouse IgG (Millipore, MAB2263); anti-GRIA2 mouse IgG (Millipore, MAB397); anti-GRIN1 rabbit IgG (Millipore, AB9864); anti-

GRIN2A rabbit IgG (Millipore, 04-901); anti-GRIN2B rabbit IgG (Millipore, AB1557P); anti-gephyrin mouse IgG (Synaptic Systems, 147 111); anti-vGAT mouse IgG (Synaptic Systems, 131 011); anti-synapsin rabbit IgG (Abcam, ab64581); anti-vGLUT1 rabbit IgG (Synaptic Systems, 135 302); anti-PSD95 mouse IgG (Abcam, ab2723); anti-BRAF mouse IgG (Santa cruz, SC-5284); anti-pERK rabbit IgG (Cell signaling, 9101); anti-ERK rabbit IgG (Cell signaling, 9102); anti-S6 mouse IgG (Cell signaling, 2317); anti-pS6 rabbit IgG (Cell signaling, 4858)] in 5% skim milk overnight at 4 °C. After washing with TBST, membranes were incubated with a secondary antibody in 5% skim milk for 1 hour at room temperature. Signals were visualized by ECL solution (Thermo).

Stereotaxic viral injection.

Virus was generated from the Institute for Basic science virus facility (IBS virus facility). 8 weeks old mice were anesthetized with ketamine solution and mounted on a stereotaxic frame. The hippocampal CA1 region was targeted using the following coordinates: anterior-posterior (AP): -1.8 mm, medial-lateral (ML): ±1.0 mm, dorsal-ventral (DV): -1.7 mm/AP: -2.5 mm, ML: ±2.0 mm, DV: -1.8 mm. AAV was injected into each point. All mice were allowed to recover for a minimum of 3 weeks before further use in experiments.

Slice preparation for 2P calcium imaging.

Brains of 9-15 weeks old mice were sliced into coronal sections (300 μm thickness) using a vibratome in ice-chilled slicing solution (75 mM NaCl, 2.5 mM KCl, 7 mM $MgCl_2$*6H_2O, 0.5 mM $CaCl_2$*2H_2O, 1.25 mM NaH_2PO_4, 26 mM $NaHCO_3$, 25 mM glucose, 75 mM sucrose)

continuously bubbled with 95% O_2 and 5% CO_2. After cutting, hippocampal slices were transferred to a holding chamber with oxygenated ACSF (125 mM NaCl, 2.5 mM KCl, 1 mM $MgCl_2$*$6H_2O$, 2 mM $CaCl_2$*$2H_2O$, 1.25 mM NaH_2PO_4, 26 mM $NaHCO_3$, 10 mM glucose) at 34 °C for 30 minutes for recovery and maintained at room temperature for and additional hours before experimental recordings.

Calcium imaging

A Thorlabs two-photon microscope (Thorlabs TIBERIUS) equipped with a 20X, 1.0 NA water-immersion objective was used for imaging the slices. GCaMP6f were excited at 920 nm, with a Mai Tai Ti:Sapphire laser. The imaging speed was set at 15.253 frame rate with 1024 x 1024 pixels in each frame. Time-lapse brain slice images were analyzed with the Fiji imaging processing package to acquire the fluorescence intensity for each region of interest (ROI) in each frame. Grid-based ROIs were determined by dividing the 290 µm x 290 µm imaging field into a uniform 36.25 µm x 36.25 µm grid. Maximal Ca^{2+} signal areas were demarcated using ImageJ (National Institutes of Health). Areas under the curve and peak numbers were calculated using GraphPad Prism. Peaks that were less than 10% of the distance from the minimum to maximum Y values or that were defined by fewer than 2 adjacent points were ignored.

Case summary of RASopathy patient

A Korean boy was delivered at 38+1 weeks of gestation with 2,644 gram of birth weight. An antenatal ultrasonography revealed a congenital heart anomaly, leading to admission to the

NICU just after birth. The boy was diagnosed with tetralogy of Fallot, and cyanotic spells and hypoxia progressively worsened. Emergency total correction was performed on the 18th day of life. Subsequently, left diaphragm palsy was developed, and moderate to severe pulmonic stenosis was identified via echocardiography. Pulmonic artery angioplasty with left diaphragm plication operation was performed at the age 6 months, feeding intolerance has been persisted, and global developmental delay was observed. Molecular genetic analysis identified a *de novo* heterozygous pathogenic variant in the *BRAF* gene, c.1495A > G (p.Lys499Glu). He underwent an additional operation for right ventricular outflow tract obstruction at the age of 4 years. Now, he is 4 years and 6 months and shows growth retardation with his height and weight below 3rd percentile, respectively. Also, he is experiencing severe developmental delays, unable to walk alone and to speak any meaningful words yet.

Generation of human cortical spheroids.

Using the CRISPR-Cas9 system, iPSC lines carrying a heterozygous and homozygous BRAF K499E mutation were generated, and the mutation was confirmed by sequencing. For Rasopathy patient cortical spheroids, peripheral blood mononuclear cells (PBMCs) were collected from the clinical diagnosed RASopathy patient described in the previous section (IRB approval number: 2204-112-1317). PBMCs were also collected from a sex- and age-matched normal subject (IRB approval number: 4-2013-0096). PBMCs were then used to derive iPSCs using the CytoTune Sendai Reprogramming vectors to deliver Oct3/4, Sox2, Klf45, and c-Myc. iPSCs were cultured on a Matrigel (Corning, 354230)-coated plate with mTeSR1 medium (STEMCELL Technologies, 85850) with the medium changed every day. We generated human cortical spheroids according to a published protocol (5). Briefly, iPSC colonies were detached

by incubation with dispase solution (0.7 mg/ml, Gibco, 17105041) for 35 min at 37 °C. Detached iPSC colonies were transferred into ultra-low attachment dishes (Corning, 4615) with iPSC medium [DMEM/F12 (Gibco, 11330057), 20% knockout serum replacement (Gibco, 10828028), non-essential amino acids (Gibco, 11140050), GlutaMax (Gibco, 35050061), 1% penicillin-streptomycin (Gibco, 15070063) and 0.1 mM β-mercaptoethanol (Sigma, M7522)] supplemented with 5 μM dorsomorphin (Sigma, P5499), 10 μM SB-431542 (Tocris, 1614) and 10 μM Y-27632(Tocris, 1254). From day 2, organoids were cultured in iPSC medium supplemented with 5 μM dorsomorphin and 10 μM SB-431542. From day 6 to day 24, organoids were cultured in neural medium [neurobasal-A medium (Gibco, 10888022), B27 supplement (-vitamin A) (Gibco, 12587010), Glutamax and 1% penicillin/streptomycin], which is supplemented with 20 ng/ml of EGF (Millipore, 01-107) and 20 ng/ml of FGF-2 (R&D Systems, 233-FB). From day 2 to day 14, medium was changed every day. From day 15, medium was changed every other day. From day 25 to day 42, organoids were cultured in neural medium supplemented with 20 ng/ml of BDNF (Peprotech, 450-02) and 20 ng/ml of NT3 (Peprotech, 450-03). From day 43, organoids were maintained in neural medium without additional factors, changing medium every four days.

Immunohistochemistry (human cortical spheroids).

Organoids were fixed with 4% paraformaldehyde for overnight at 4 °C and transferred into 30% (wt/vol) sucrose solution for 48 h at 4 °C. Organoids were embedded in OCT compound (Leica, 14020108926) and snap-frozen with liquid nitrogen. For immunohistochemistry, blocks were cut into 10 μm-thick sections using a cryostat. Sections were permeabilized and blocked with 10% normal goat serum (Abcam, ab7481) and 0.3% (vol/vol) Triton X-100 in PBS for 1 h at

room temperature. Primary antibodies [anti-GFAP guinea pig (Synaptic Systems, 173-004), anti-pERK rabbit IgG (Cell Signaling Technology, 4377S), anti-S100β rabbit IgG (Abcam, ab52642) and anti-Ki67 mouse IgG (Cell signaling technology, 9449S)] were diluted in 2% normal goat serum and 0.1% (vol/vol) Triton X-100 in PBS and incubated overnight at 4 °C. Secondary antibodies were incubated for 1 h at room temperature. Images were obtained with LSM-980 confocal microscopy (Zeiss).

Transcriptome analysis.

RNA samples were collected from mouse hippocampus and human spheroid. We obtained 11 to 15 weeks old five wild-type male mice and four Nestin;BRAF$^{KE/+}$ male mice, and three wild-type spheroids and three KE spheroids samples for RNA-sequencing analysis [Gene Expression Omnibus Series GSE234764]. Read alignment and processing were performed using the Nextflow RNAseq pipeline (v3.2, https://github.com/nf-core/rnaseq). Raw reads were trimmed with TrimGalore (https://github.com/FelixKrueger/TrimGalore), aligned against reference using STAR (Gencode vM28, GRCm39 for mouse; Gencode v39, GRCh38 for human) (6), and quantified with Salmon (7). Differential analyses were performed after adjusting batches using R package DESeq2 (v1.28.14) (8). Gene set enrichment analysis (GSEA) was conducted with R package gprofiler2 (v0.2.04) (9) and fgsea (v1.14.04) (10). For pathway enrichment analysis, terms were obtained from MsigDB Gene Ontology (11). Differentially expressed genes with absolute $\log_2$ fold change ($\log_2$FC) > 0.5 for BRAF KE mouse and > 1.5 for BRAF KE spheroid and astrocyte were used for testing. Pathway terms with a size smaller than 30 or larger than 1,000 were excluded from the results. For enrichment test using MsigDB cell type signature gene sets, gene sets associated with reactive astrocytes

and microglial activation was obtained from previously reported studies (12-14).

Primary astrocyte culture.

Cortical astrocytes were prepared from embryos from C57BL/6 mice on embryonic day 18. Cortices were dissected in HBSS (Thermo, 14170112) at 4 °C and then incubated in 0.25% trypsin-EDTA (Thermo, 25200056) in a 37 °C for 20 minutes with gentle inversion every 5 minutes. After trypsinization, the tissue was washed in HBSS (Thermo, 14170112) at 4 °C five times and then triturated with 1 ml of culture media [10% FBS (Thermo, 16250-078), 5000 U/ml penicillin and 5000 μg/ml streptomycin (Thermo, 15070063) in DMEM (HyClone, SH30243.01)]. Dissociated cells were filtered through a cell strainer (BD Falcon, 352360) and plated on 0.04% polyethylenimine (Sigma, P3143)-coated cell culture dishes (4×10^6 cells/60 mm dish) in culture media. Seven days after plating the cells, the dishes were shaken at 110 rpm for 6 hours. The cells were then washed with DPBS three times, treated with 0.25% trypsin, and plated on 0.04% polyethylenimine coated 18 mm coverslips in a 12-well plate (3×10^4 cells/well) in astrocyte culture media [2% B-27 supplement (Thermo, 17504044), 2mM GlutaMax (Thermo, 35050061), 5000 U/ml penicillin and 5000 μg/ml streptomycin (Thermo, 15070063), 5 ng/ml HB-EGF (Sigma, E4643) in neurobasal medium (Thermo, 21103049)].

References

1. Ryu, H.H., et al., Neuron type-specific expression of a mutant KRAS impairs hippocampal-dependent learning and memory. *Sci Rep*, 2020;10(1):17730.
2. Kang, M., H.H. Ryu, and Y.S. Lee, Comparisons of behavior and synaptic plasticity among three C57BL/6 substrains. *Animal Cells and Systems*, 2015;19(3):181-187.
3. Ryu, H.H., et al., Excitatory neuron-specific SHP2-ERK signaling network regulates synaptic plasticity and memory. *Sci Signal*, 2019;12(571).
4. Chun, H., et al., Severe reactive astrocytes precipitate pathological hallmarks of Alzheimer's disease via H2O2− production. *Nature Neuroscience*, 2020;23(12):1555-1566.
5. Paşca, A.M., et al., Functional cortical neurons and astrocytes from human pluripotent stem cells in 3D culture. *Nat Methods*, 2015;12(7):671-8.
6. Dobin, A., et al., STAR: ultrafast universal RNA-seq aligner. *Bioinformatics*, 2013;29(1):15-21.
7. Patro, R., et al., Salmon provides fast and bias-aware quantification of transcript expression. *Nat Methods*, 2017;14(4):417-419.
8. Love, M.I., W. Huber, and S. Anders, Moderated estimation of fold change and dispersion for RNA-seq data with DESeq2. *Genome Biol*, 2014;15(12):550.
9. Kolberg, L., et al., gprofiler2 -- an R package for gene list functional enrichment analysis and namespace conversion toolset g:Profiler. *F1000Res*, 2020;9.
10. Gennady, K., et al., Fast gene set enrichment analysis. *bioRxiv*, 2021:060012.
11. Harris, M.A., et al., The Gene Ontology (GO) database and informatics resource. *Nucleic Acids Res*, 2004;32(Database issue):D258-61.
12. Zamanian, J.L., et al., Genomic analysis of reactive astrogliosis. *J Neurosci*, 2012;32(18):6391-410.
13. Escartin, C., et al., Reactive astrocyte nomenclature, definitions, and future directions. *Nature Neuroscience*, 2021;24(3):312-325.
14. Yu, X., et al., Reducing Astrocyte Calcium Signaling In Vivo Alters Striatal Microcircuits and Causes Repetitive Behavior. *Neuron*, 2018;99(6):1170-1187.e9.

Supplemental figures

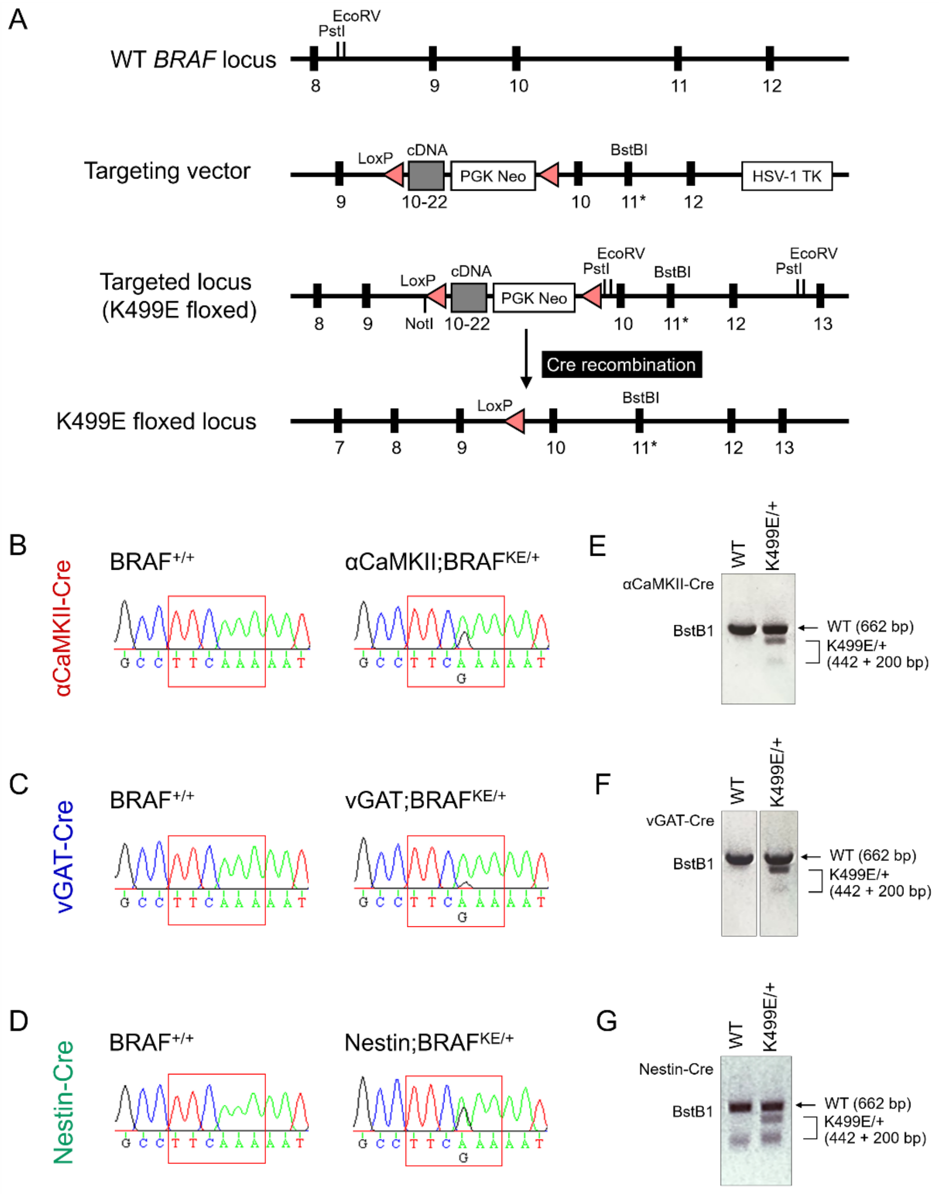

Supplemental figure 1. Generation of inducible BRAF$^{KE/+}$ knock-in mice. (A) Structures of the BRAF locus, targeting vector, mutant allele are shown. The asterisk (*) indicates the exon containing the KE mutation. Sanger sequencing confirming the presence of heterozygous BRAF KE mutations in **(B)** αCaMKII;BRAF$^{KE/+}$, **(C)** vGAT;BRAF$^{KE/+}$, and **(D)** Nestin;BRAF$^{KE/+}$ mice. **(E-G)** Total RNA was isolated from wild-type and BRAF$^{KE/+}$ hippocampi and transcribed into cDNA. PCR products from mutant cDNAs were digested with BstBI.

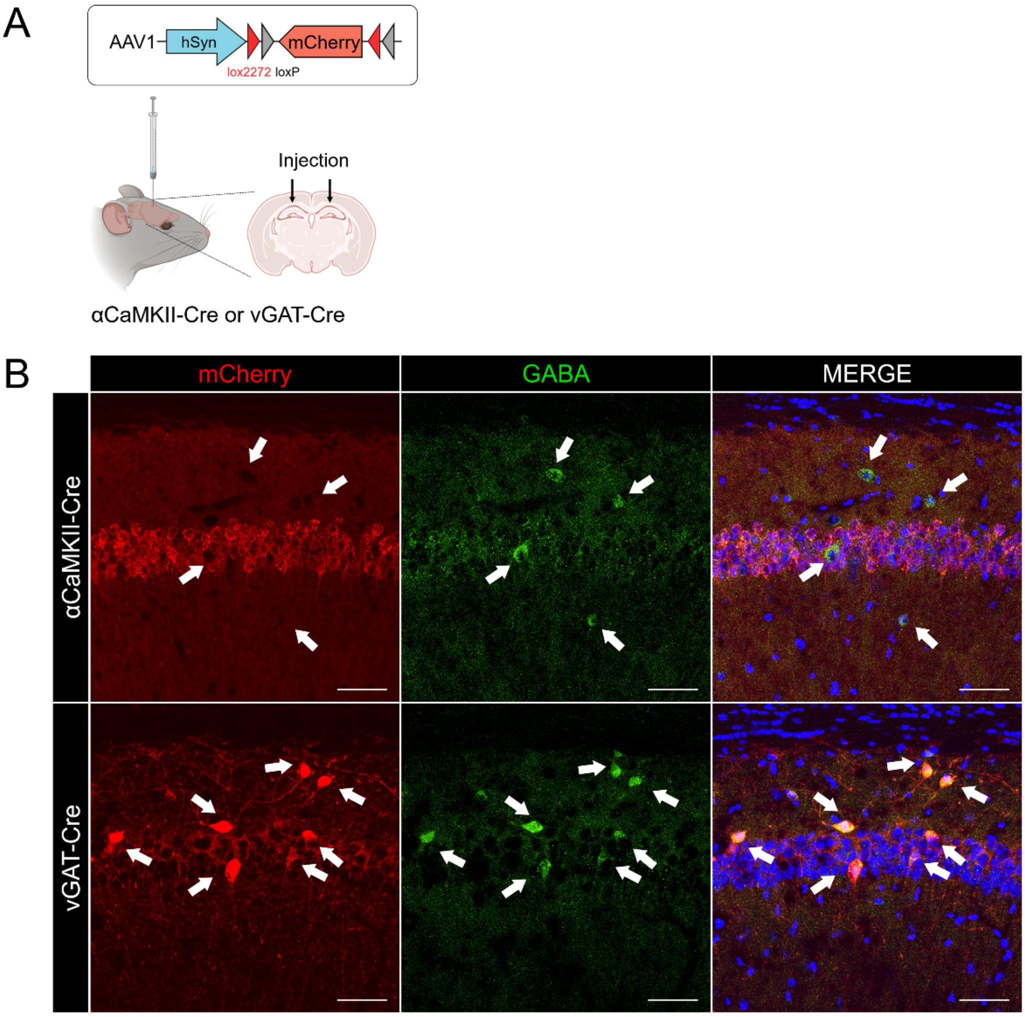

Supplemental figure 2. Validation of the cell type-specificity of αCaMKII-Cre and vGAT-Cre mice. (A) Schematic illustrating the experimental approach in which AAV carrying hSyn-DIO-mCherry were injected into the hippocampal CA1 region of αCaMKII-Cre and vGAT-Cre mice. **(B)** Representative confocal images showing mCherry (red), GABA immunolabeling (green), and DAPI (blue) in the CA1 region of the adult hippocampus of αCaMKII-Cre and vGAT-Cre mice injected with hSyn-DIO-mCherry virus. Arrows indicate $GABA^+$ cells. Scale bars, 50 µm.

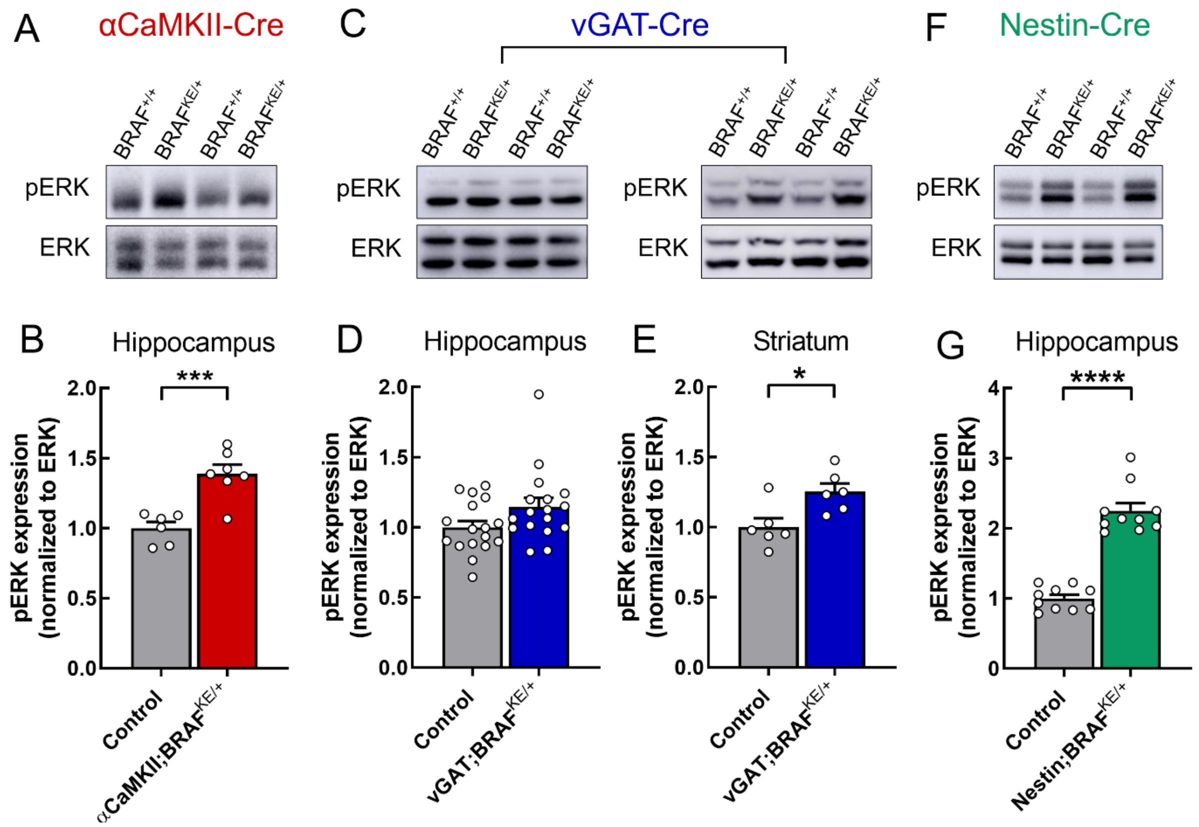

Supplemental figure 3. Effect of BRAF KE on RAS-ERK signaling activity in inducible BRAF^{KE} knock-in mice. (A) Representative p-ERK1/2 and ERK1/2 immunoblot images of hippocampal lysates from BRAF$^{+/+}$ or αCaMKII;BRAF$^{KE/+}$ mice. **(B)** Quantification of p-ERK1/2 expression in control (BRAF$^{+/+}$ or BRAF$^{KE\ floxed/+}$, n = 6) or αCaMKII;BRAF$^{KE/+}$ (n = 7) hippocampus (***p < 0.001 by unpaired t test). **(C)** Representative p-ERK1/2 and ERK1/2 immunoblot images of hippocampal or striatal lysates from BRAF$^{+/+}$ or vGAT;BRAF$^{KE/+}$ mice. **(D)** Quantification of p-ERK1/2 expression in control (n = 15) or vGAT;BRAF$^{KE/+}$ (n = 17) hippocampus (p = 0.0674 by unpaired t test). **(E)** Quantification of p-ERK1/2 expression in control (n = 6) or vGAT;BRAF$^{KE/+}$ (n = 6) striatum (*p < 0.05 by unpaired t test). **(F)** Representative p-ERK1/2 and ERK1/2 immunoblot images of hippocampal lysates from BRAF$^{+/+}$ or Nestin;BRAF$^{KE/+}$ mice. **(G)** Quantification of p-ERK1/2 expression in control (n = 10) or Nestin;BRAF$^{KE/+}$ (n = 10) hippocampus (****p < 0.0001 by unpaired t test). Data are expressed as means ± SEM.

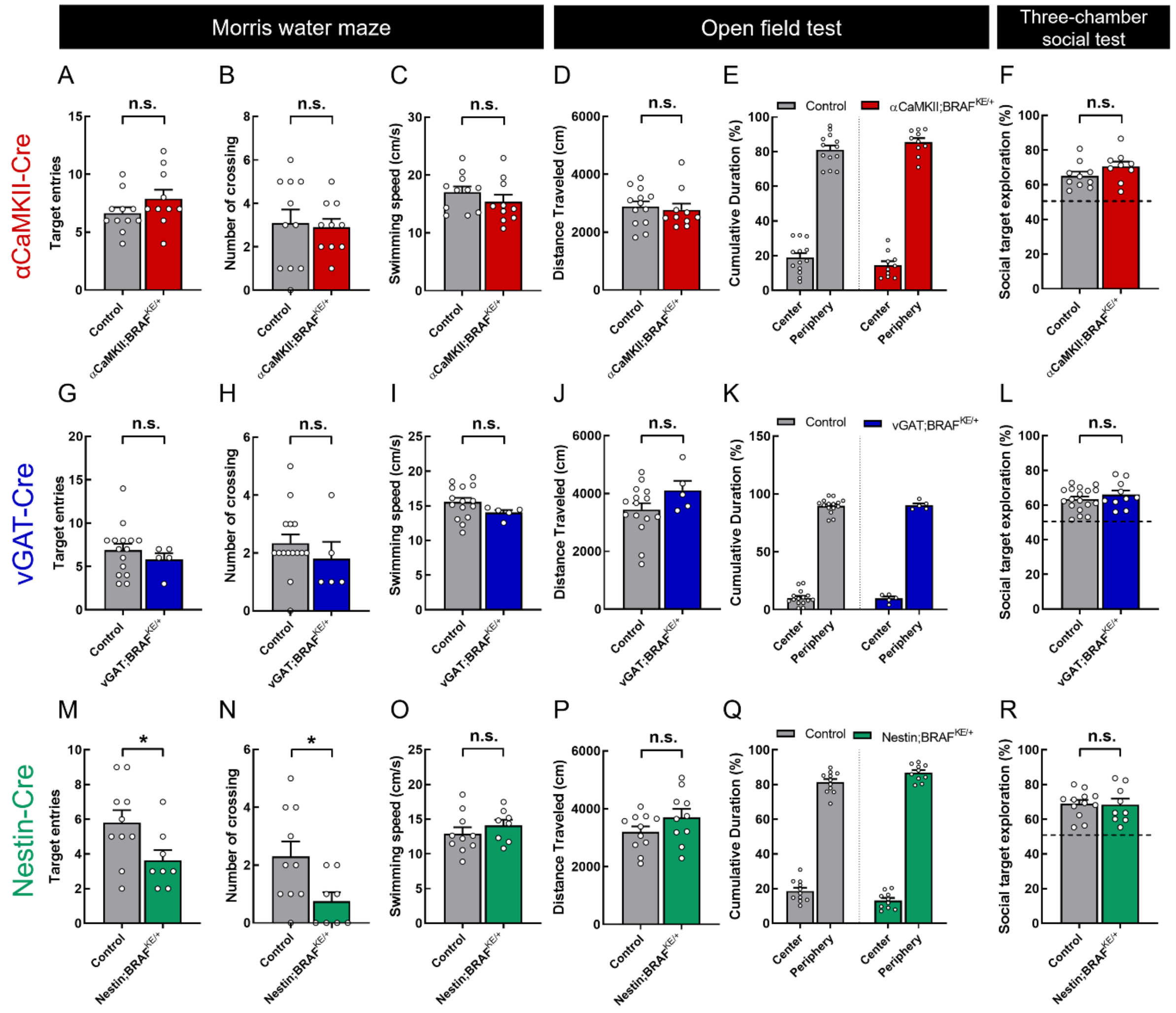

Supplemental figure 4. BRAF KE expression in excitatory neurons or inhibitory neurons does not significantly affect behavior. (A) Number of target zone entries of control and αCaMKII;BRAF$^{KE/+}$ mice during the Morris water maze (MWM) probe trial (control, n = 11; αCaMKII;BRAF$^{KE/+}$, n = 10; n.s., *p* = 0.1786 by unpaired *t* test). **(B)** Platform crossings for the mice in A (n.s., *p* = 0.8015 by unpaired *t* test). **(C)** Swimming speed of control or αCaMKII;BRAF$^{KE/+}$ mice during the MWM probe trial (n.s., *p* = 0.2981 by unpaired *t* test). **(D)** Total distance traveled during the open field test for control (n = 13) and αCaMKII;BRAF$^{KE/+}$ (n = 10) mice (n.s., *p* = 0.6807 by unpaired *t* test). **(E)** Cumulative duration in the center or periphery zones of open field arena for mice in D. **(F)** Percentage of time spent exploring the social target in a three-chamber social interaction test for control (n = 10) or αCaMKII;BRAF$^{KE/+}$ (n = 9) mice (n.s., *p* = 0.1662 by unpaired *t* test). **(G)** Number of target zone entries of control and

vGAT;BRAF$^{KE/+}$ mice during the MWM probe trial (control, n = 15; vGAT;BRAF$^{KE/+}$, n = 5; n.s., *p* = 0.4496 by unpaired *t* test). **(H)** Number of platform crossings for mice in G (n.s., *p* = 0.4026 by unpaired *t* test). **(I)** Swimming speed in the MWM probe trial for mice in G (n.s., *p* = 0.1834 by unpaired *t* test). **(J)** Total distance traveled during the open field test for control (n = 15) and vGAT;BRAF$^{KE/+}$ (n = 5) mice (n.s., *p* = 0.1445 by unpaired *t* test). **(K)** Cumulative duration in the center or periphery zones of open field arena for mice in J. **(L)** Percentage of time spent exploring the social target in a three chamber social interaction test for control (n = 18) and vGAT;BRAF$^{KE/+}$ (n = 10) mice (n.s., *p* = 0.3533 by unpaired *t* test). **(M)** Number of target zone entries of control and Nestin;BRAF$^{KE/+}$ mice (control, n = 10; Nestin;BRAF$^{KE/+}$**,** n = 8; *p < 0.05 by unpaired *t* test). **(N)** Number of platform crossings for mice in M (*p < 0.05 by unpaired *t* test). **(O)** Swimming speed in the MWM probe trial for mice in M (n.s., *p* = 0.3635 by unpaired *t* test). **(P)** Total distance traveled during the open field test for control (n = 11) and Nestin;BRAF$^{KE/+}$ (n = 10) mice (n.s., *p* = 0.1454 by unpaired *t* test). **(Q)** Cumulative duration in the center or periphery zones of open field arena for mice in P. **(R)** Percentage of time spent exploring the social target in a three chamber social interaction test for control (n = 12) and Nestin;BRAF$^{KE/+}$ (n = 9) mice (n.s., *p* = 0.8879 by unpaired *t* test). Data are expressed as means ± SEM.

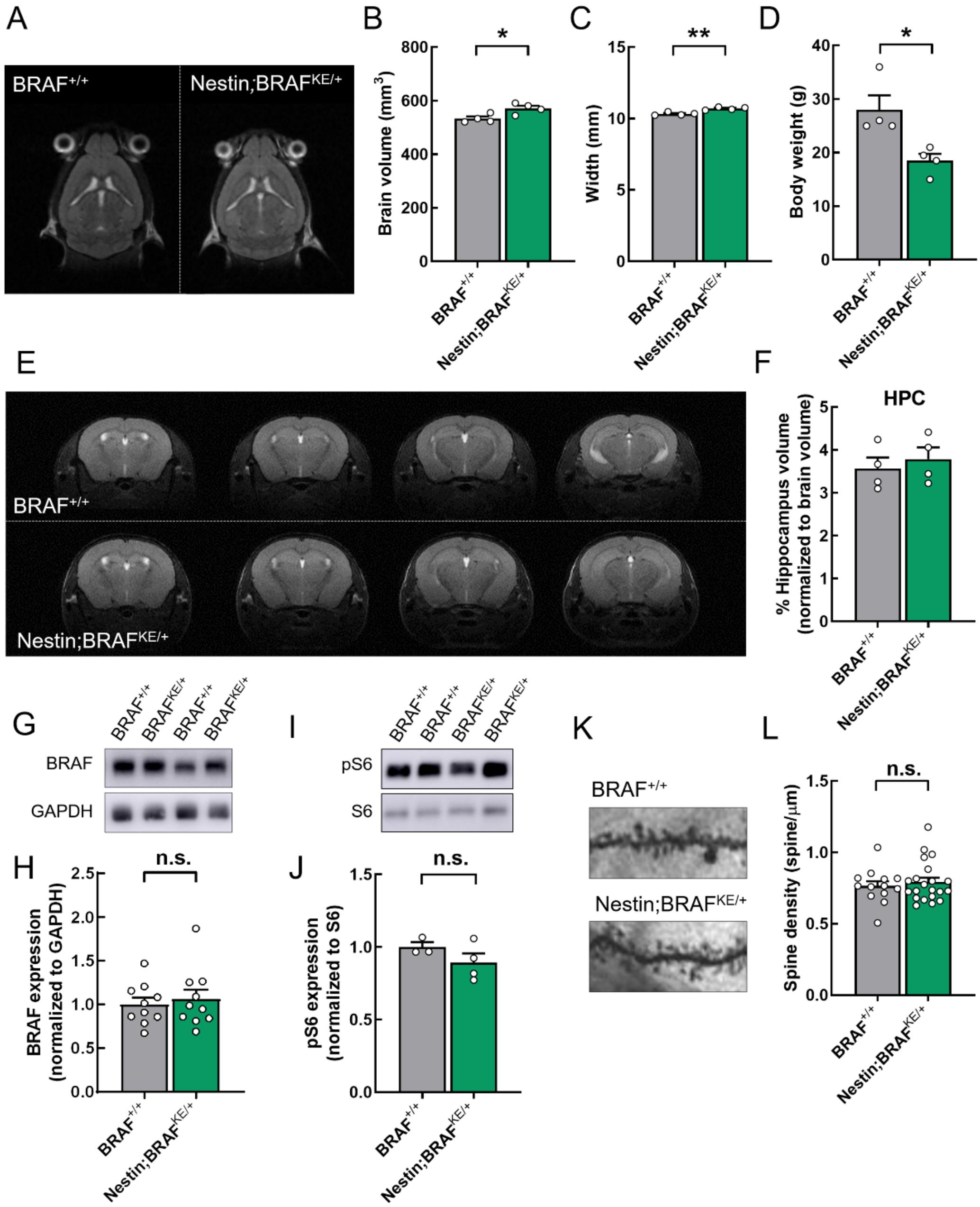

Supplemental figure 5. BRAF KE in neural stem cells does not have a significant effect on brain structure. (A) Representative horizontal image of structural magnetic resonance imaging (MRI) of brains from BRAF$^{+/+}$ or Nestin;BRAF$^{KE/+}$ mice. **(B-D)** (B) Brain volume and (C) width, or (D) total body weight for BRAF$^{+/+}$ (n = 4) or Nestin;BRAF$^{KE/+}$ (n = 4) mice (*p < 0.05, **p < 0.01 by unpaired t test). **(E)** Representative coronal MRI images of the hippocampus in BRAF$^{+/+}$ or Nestin;BRAF$^{KE/+}$ mice. **(F)** Hippocampal volume normalized to whole brain volume in

BRAF$^{+/+}$ (n = 4) or Nestin;BRAF$^{KE/+}$ (n = 4) mice (n.s., p = 0.5803 by unpaired t test). HPC: hippocampus. **(G)** Representative BRAF and GAPDH immunoblot images of hippocampal lysates from BRAF$^{+/+}$ and Nestin;BRAF$^{KE/+}$ mice. **(H)** Quantification of BRAF expression in BRAF$^{+/+}$ (n = 10) and Nestin;BRAF$^{KE/+}$ (n = 10) mice (n.s., p = 0.6516 by unpaired t test). **(I)** Representative p-S6 and S6 immunoblot images of hippocampal lysates from BRAF$^{+/+}$ or Nestin;BRAF$^{KE/+}$ mice. **(J)** Quantification of p-S6 expression in BRAF$^{+/+}$ (n = 3) and Nestin;BRAF$^{KE/+}$ (n = 4) mice (n.s., p = 0.2397 by unpaired t test). **(K)** Representative Golgi-stained images of pyramidal neuronal dendrites at hippocampal CA1. Scale bar, 10 μm. **(L)** Spine density in hippocampal CA1 in BRAF$^{+/+}$ (n = 13 neurons from 4 mice) and Nestin;BRAF$^{KE/+}$ mice (n = 21 neurons from 4 mice; n.s., p = 0.5650 by unpaired t test). Data are expressed as means ± SEM.

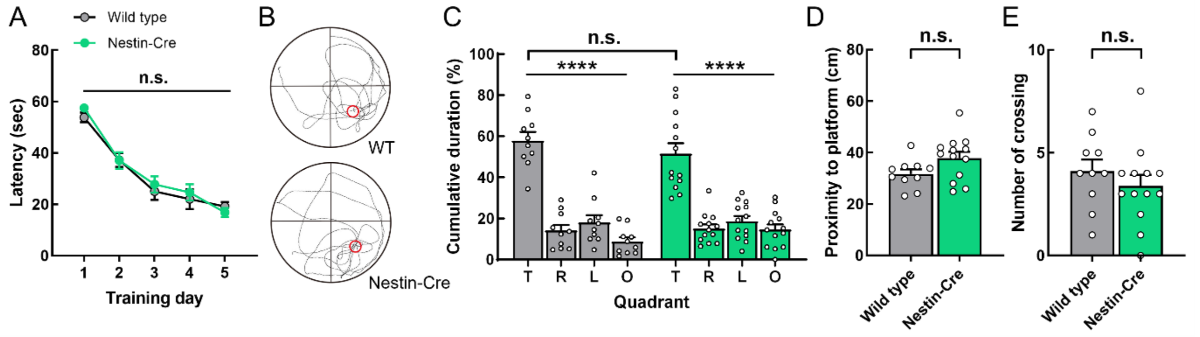

Supplemental figure 6. Nestin-Cre mice performed similarly to wild-type littermates in the Morris water maze. (A) Learning curve showing the time required (latency) for wild-type (n = 10) and Nestin-Cre (n = 13) mice to find the hidden platform in Morris water maze (MWM) training trials (effect of genotype, $F_{1,\,21} = 0.3402$, n.s., $p = 0.5659$ by two-way ANOVA). **(B)** Representative swimming trajectory of wild-type and Nestin-Cre mice in the MWM probe trial. **(C)** Cumulative duration of time spent in each quadrant during the probe trial in B ($F_{3,\,36} = 48.94$, ****$p < 0.0001$ for wild-type; $F_{3,\,48} = 29.59$, ****$p < 0.0001$ for Nestin-Cre by one-way ANOVA; n.s., $p = 0.3630$ by unpaired *t* test). T, target quadrant; R, right quadrant; L, left quadrant; O, opposite quadrant. **(D)** Proximity of mice in C to the target platform (n.s., $p = 0.0606$ by unpaired *t* test). **(E)** Number of platform crossings for the mice in C (n.s., $p = 0.3751$ by unpaired *t* test). Data are expressed as means ± SEM.

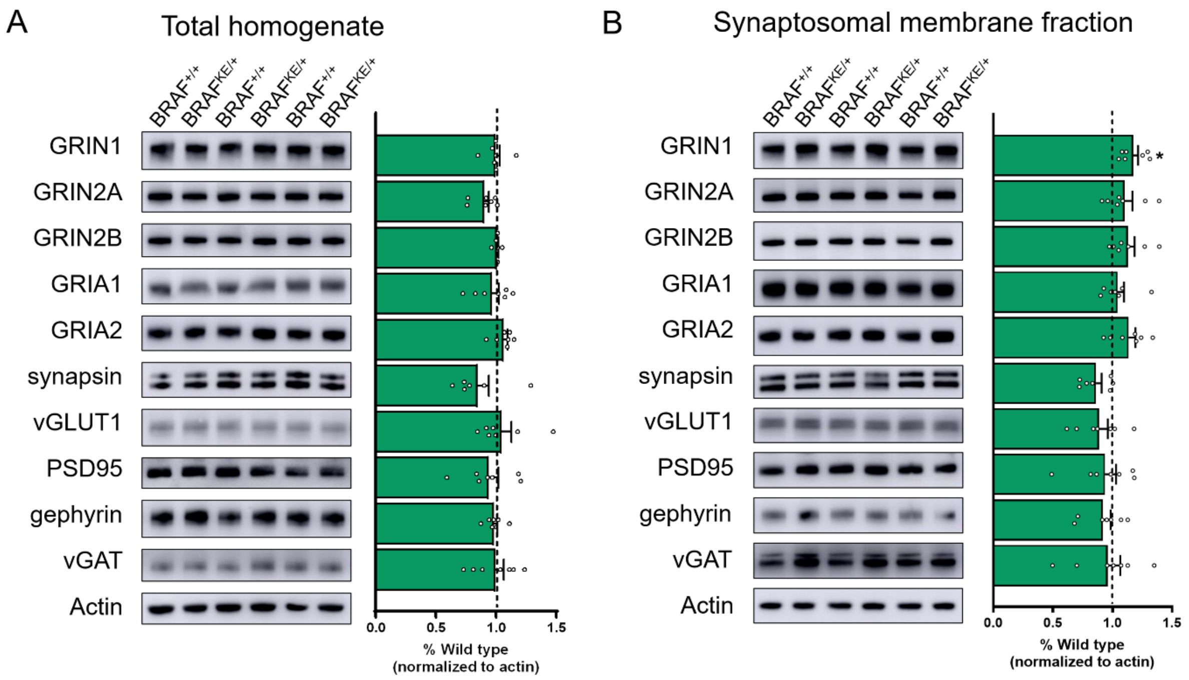

Supplemental figure 7. BRAF KE in neural stem cells does not dramatically alter the expression of hippocampal synaptic proteins. (A) Representative western blot of total homogenate and **(B)** synaptosomal membrane fractions derived from the hippocampi of $BRAF^{+/+}$ (n = 7) and Nestin;$BRAF^{KE/+}$ (n = 6-7) mice (*p < 0.05 by unpaired t test). Each histogram shows protein levels normalized to actin. Data are expressed as means ± SEM.

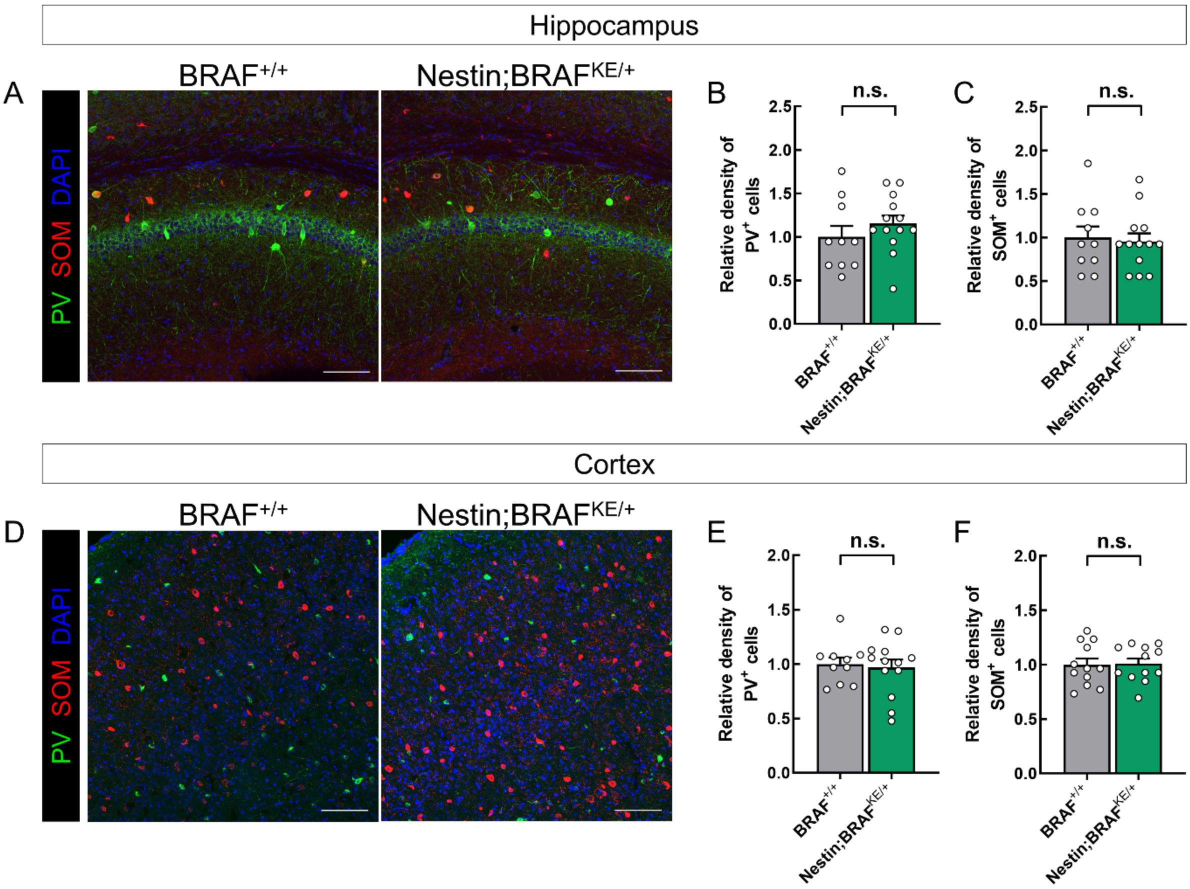

Supplemental figure 8. Parvalbumin- and somatostatin-positive inhibitory neuron populations are unaltered in the hippocampal CA1 region and cortex of Nestin;BRAF$^{KE/+}$ mice. (A) Representative confocal images of immunolabeling for parvalbumin (PV, green) and somatostatin (SOM, red) in the CA1 region of the adult hippocampus of BRAF$^{+/+}$ or Nestin;BRAF$^{KE/+}$ mice. Scale bar, 100 μm. **(B and C)** Relative density of (B) PV-positive or (C) SOM-positive cells in the hippocampal CA1 region of BRAF$^{+/+}$ (n = 10 images from 3 mice) or Nestin;BRAF$^{KE/+}$ (n = 13 images from 3 mice) mice (n.s., p = 0.3284 for B, n.s., p = 0.7701 for C by unpaired t test). **(D)** Representative confocal images of immunolabeling for PV (green) and SOM (red) in the cortex of BRAF$^{+/+}$ or Nestin;BRAF$^{KE/+}$ mice. Scale bar, 100 μm. **(E and F)** Relative density of (E) PV-positive or (F) SOM-positive cells in the cortex of BRAF$^{+/+}$ (PV, n = 10 images from 3 mice; SOM, 12 images from 3 mice) or Nestin;BRAF$^{KE/+}$ (PV, n = 13 images from 4 mice; SOM, 12 images from 4 mice) mice (n.s., p = 0.7662 for E, n.s., p = 0.8961 for F by unpaired t test). Data are expressed as means ± SEM.

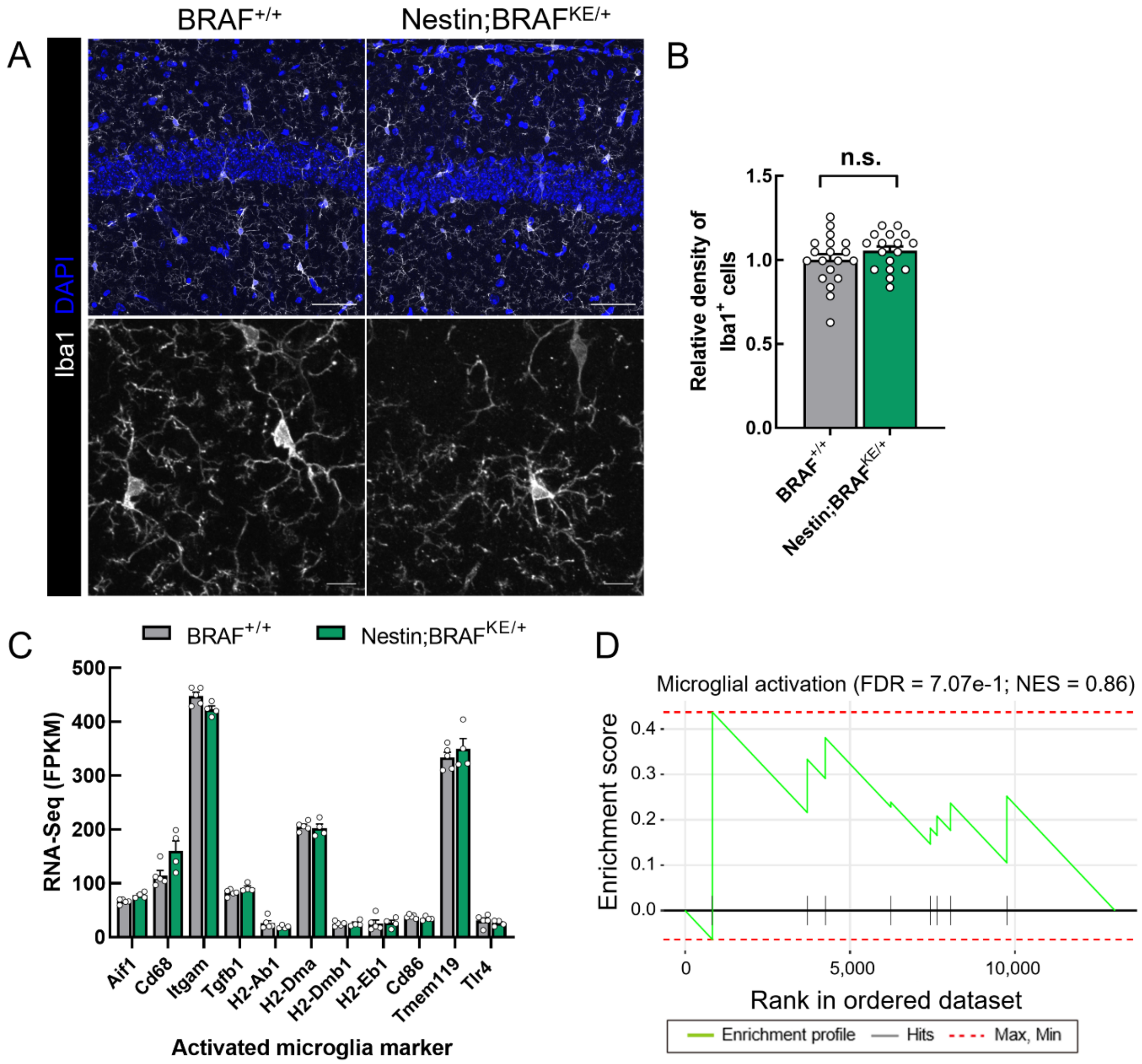

Supplemental figure 9. Nestin;BRAF$^{KE/+}$ mice do not exhibit microglial activation in the hippocampus. (A) Representative confocal images of Iba1 (white) immunolabeling in the hippocampal CA1 region of BRAF$^{+/+}$ and Nestin;BRAF$^{KE/+}$ mice. Scale bars, 50 μm, 10 μm. **(B)** Relative density of Iba1-positive cells from the experiments depicted in A (BRAF$^{+/+}$, n = 19 images from 5 mice; Nestin;BRAF$^{KE/+}$, n = 17 images from 5 mice; n.s., p = 0.2364 by unpaired t test). **(C)** FPKM values for genes associated with activated microglia in the BRAF$^{+/+}$ (n = 5) and Nestin;BRAF$^{KE/+}$ (n = 4) transcriptomes. **(D)** Enrichment plot for microglial activation gene sets. FPKM, fragments per kilobase of transcript per million mapped reads. Data are expressed as means ± SEM.

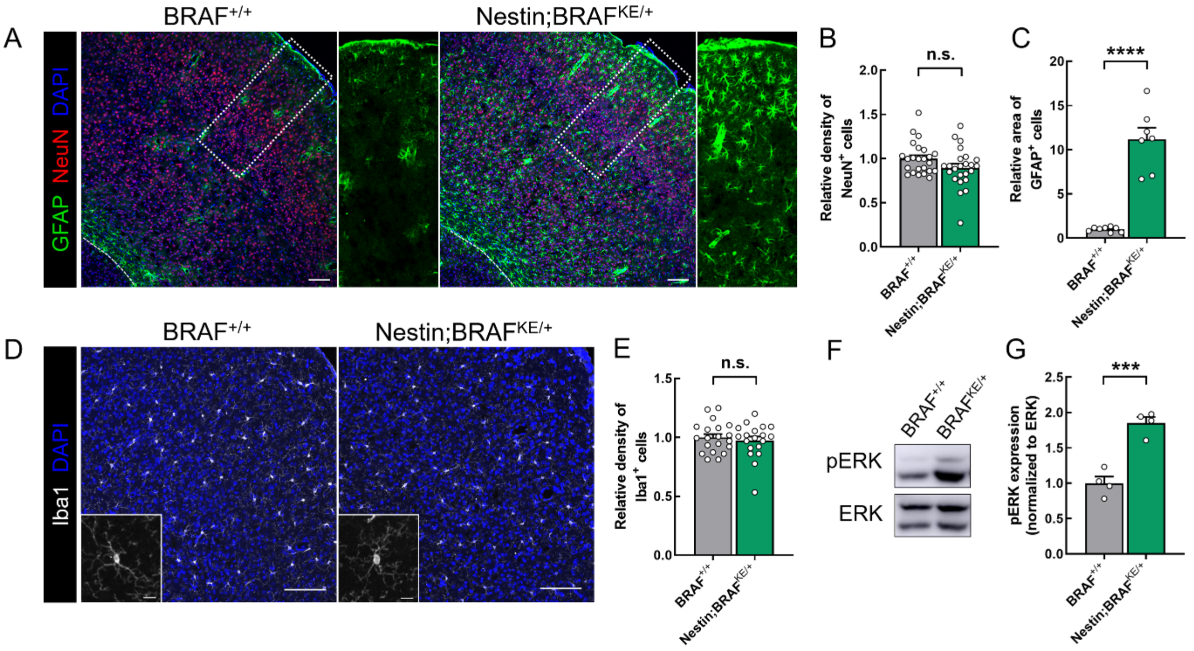

Supplemental figure 10. Nestin;BRAF$^{KE/+}$ mice exhibit significant increases in GFAP expression in the somatosensory cortex. (A) Representative confocal images of GFAP (green) and NeuN (red) immunolabeling in the adult somatosensory (S1) cortex of BRAF$^{+/+}$ or Nestin;BRAF$^{KE/+}$ mice. Scale bars, 100 μm. **(B)** Number of NeuN-positive cells in the S1 cortex of BRAF$^{+/+}$ (n = 24 images from 3 mice) or Nestin;BRAF$^{KE/+}$ (n = 24 images from 3 mice) mice (n.s., p = 0.0804 by unpaired t test). **(C)** Relative area of GFAP-expressing cells in brains from BRAF$^{+/+}$ (n = 8 images from 2 mice) or Nestin;BRAF$^{KE/+}$ (n = 7 images from 2 mice) mice (****p < 0.0001 by unpaired t test). **(D)** Representative confocal images of Iba1 immunolabeling in adult S1 cortex of BRAF$^{+/+}$ or Nestin;BRAF$^{KE/+}$ mice. Scale bars, 100 μm; 10 μm. **(E)** Number of Iba1-positive cells in the S1 cortex of BRAF$^{+/+}$ (n = 20 images from 5 mice) or Nestin;BRAF$^{KE/+}$ (n = 19 images from 5 mice) mice (n.s., p = 0.5530 by unpaired t test). **(F)** Representative p-ERK1/2 and ERK1/2 immunoblot image of cortical lysates from BRAF$^{+/+}$ or Nestin;BRAF$^{KE/+}$ mice. **(G)** Quantification of p-ERK1/2 expression from F (BRAF$^{+/+}$, n = 4; Nestin;BRAF$^{KE/+}$, n = 4; ***p < 0.001 by unpaired t test). Data are expressed as means ± SEM.

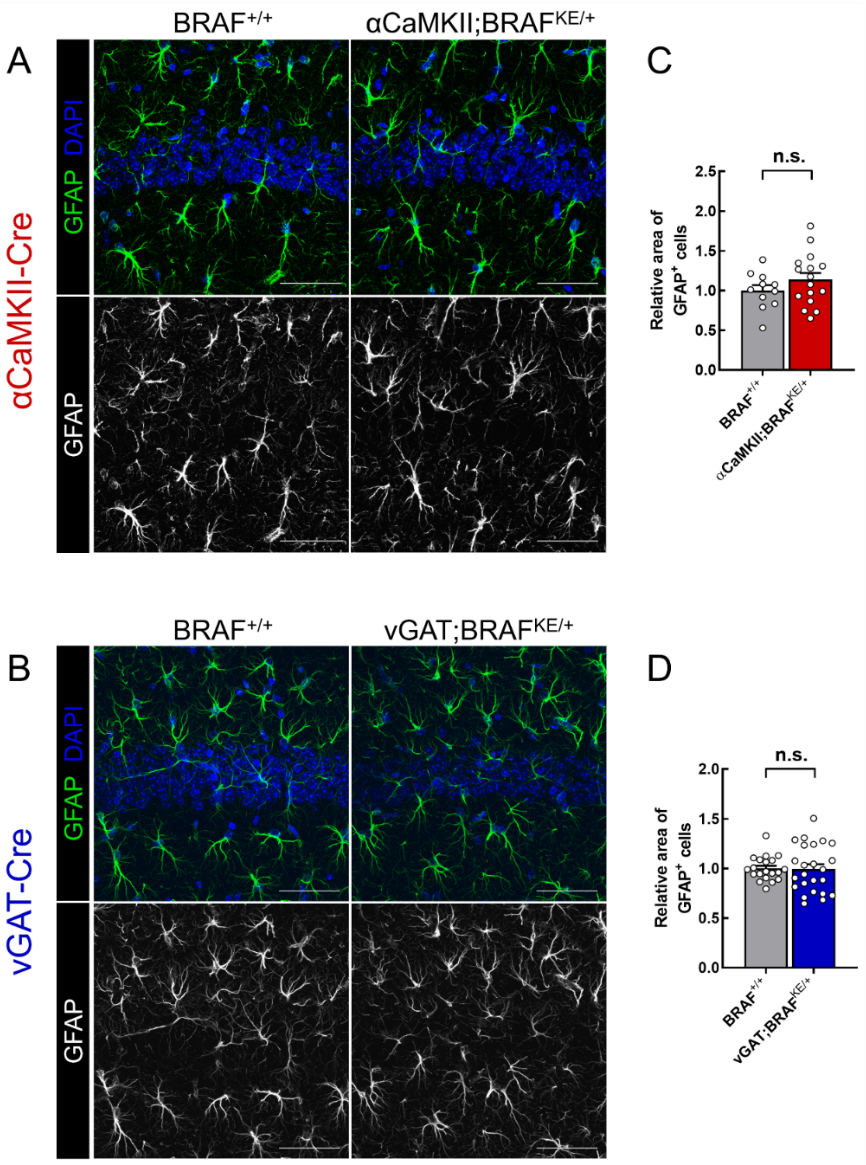

Supplemental figure 11. αCaMKII;BRAF$^{KE/+}$ and vGAT;BRAF$^{KE/+}$ mice do not exhibit increased GFAP expression in the hippocampus. (A and B) Representative confocal images of GFAP (green) immunolabeling in the hippocampal CA1 region of (A) adult BRAF$^{+/+}$ or αCaMKII;BRAF$^{KE/+}$ mice or (B) adult BRAF$^{+/+}$ or vGAT;BRAF$^{KE/+}$ mice. Scale bars, 50 μm. **(C)** Quantification of GFAP-positive cells in the mice in A (BRAF$^{+/+}$, n = 11 images from 4 mice; αCaMKII;BRAF$^{KE/+}$, n = 16 images from 6 mice; n.s., p = 0.2404 by unpaired t test). **(D)** Quantification of GFAP-positive cells in the mice in B (BRAF$^{+/+}$, n = 20 images from 4 mice; vGAT;BRAF$^{KE/+}$, n = 24 images from 5 mice; n.s., p = 0.9397 by unpaired t test). Data are expressed as means ± SEM.

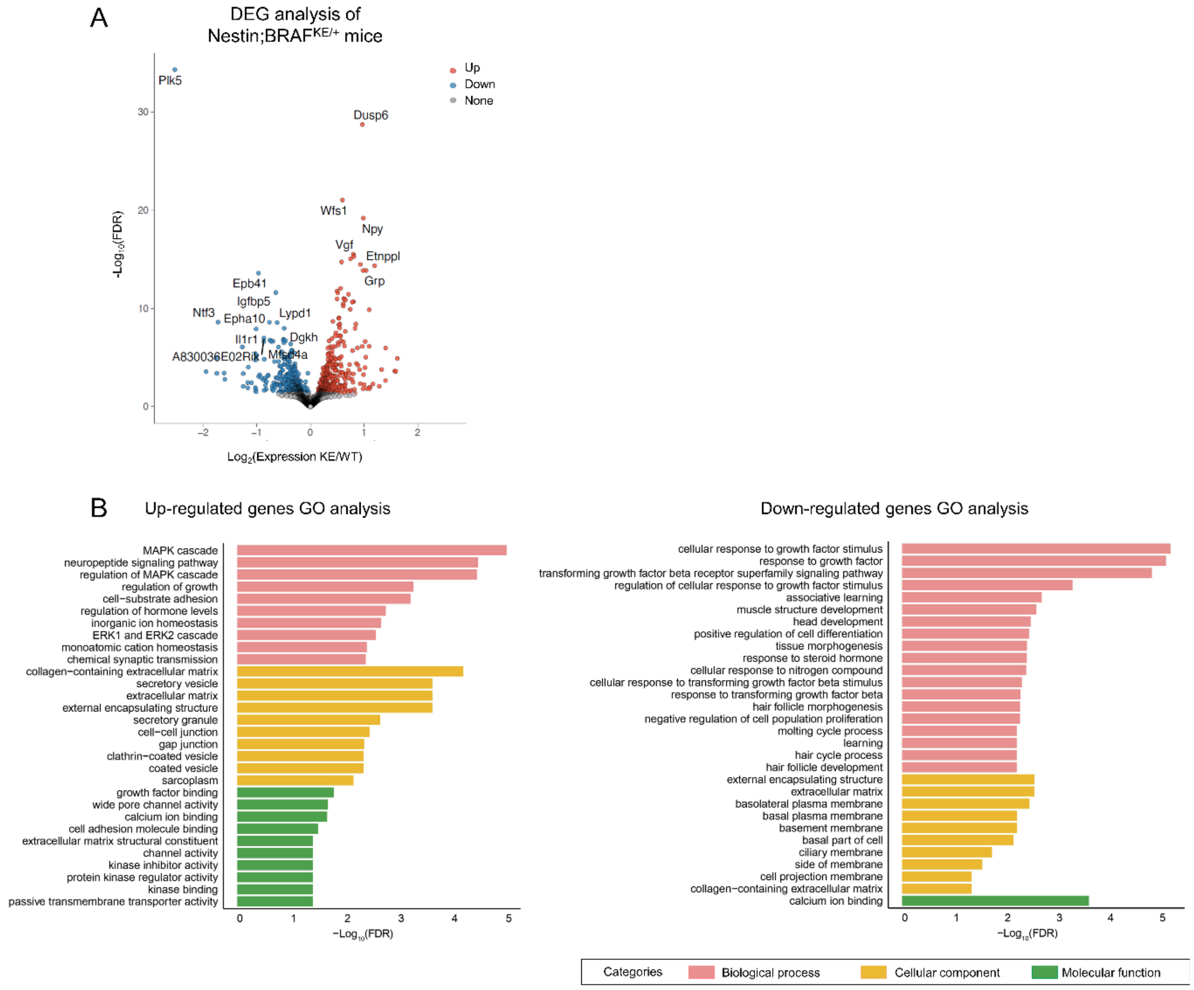

Supplemental figure 12. Gene ontology enrichment analysis of genes differentially expressed in the hippocampus of Nestin;BRAF$^{KE/+}$ mice. (A) Volcano plot depicting RNA-seq data from BRAF$^{+/+}$ and Nestin;BRAF$^{KE/+}$ mice. **(B)** Gene ontology enrichment analysis of DEGs up- or down-regulated in Nestin;BRAF$^{KE/+}$ mice, showing various biological processes, molecular functions, and cellular components.

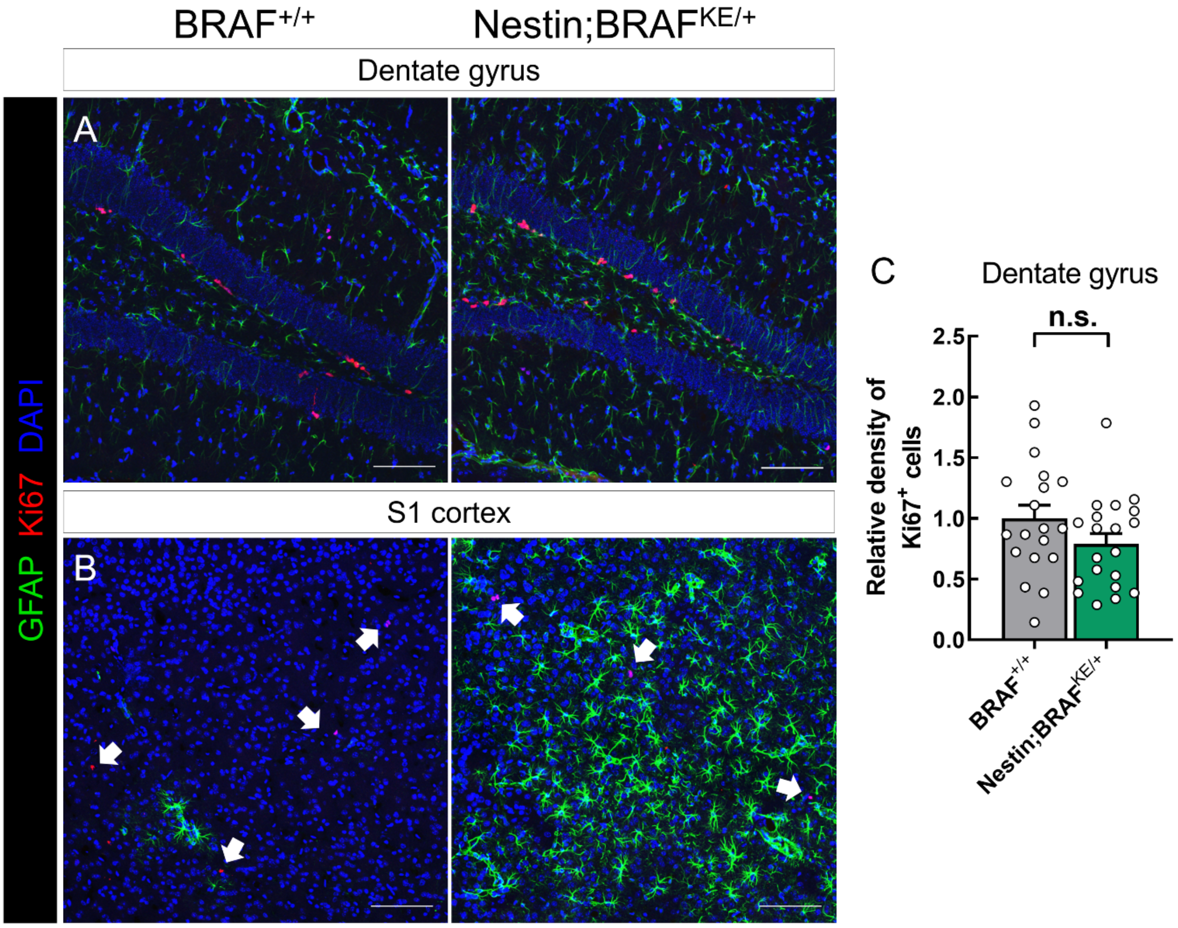

Supplemental figure 13. GFAP-positive astrocyte cells from Nestin;BRAF$^{KE/+}$ mice do not exhibit increased proliferation in the adult period. (A and B) Representative confocal images of Ki67 (red) and GFAP (green) immunolabeling in the (A) adult hippocampus dentate gyrus or (B) adult S1 cortex from BRAF$^{+/+}$ or Nestin;BRAF$^{KE/+}$ mice. Scale bars, 100 μm. Arrows indicate Ki67^{+} cells. **(C)** Relative density of Ki67-expressing cells from the dentate gyrus in A (BRAF$^{+/+}$, n = 19 images from 5 mice; Nestin;BRAF$^{KE/+}$, n = 20 images from 5 mice; n.s., p = 0.1315 by unpaired t test). Data are expressed as means ± SEM.

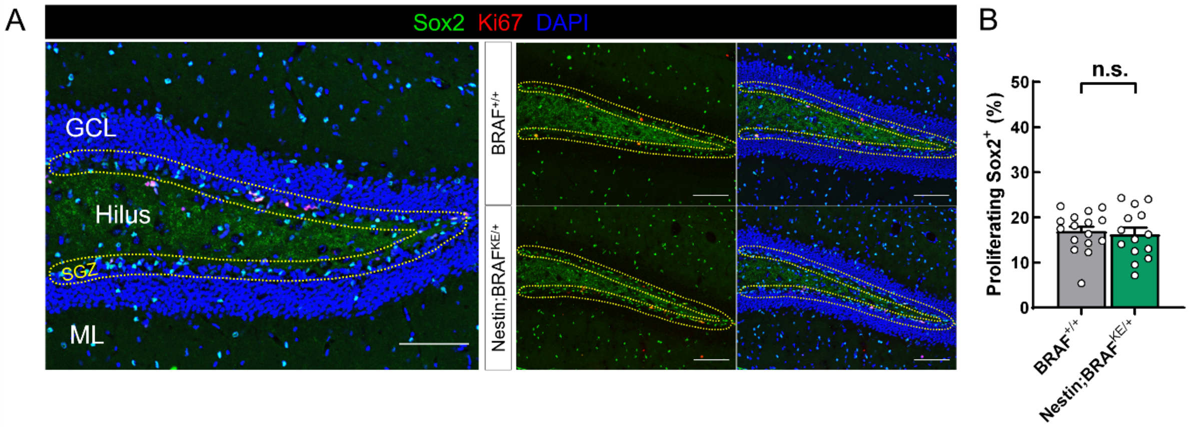

Supplemental figure 14. Nestin;BRAF$^{KE/+}$ mice do not exhibit increased proliferating Sox2-positive cells in the dentate gyrus. (A) Representative confocal images of immunolabeling for Sox2 (green) and Ki67 (red) in the dentate gyrus of BRAF$^{+/+}$ or Nestin;BRAF$^{KE/+}$ mice. Dotted yellow lines indicate the subgranular zone (SGZ). GCL, granule cell layer; ML, molecular layer. Scale bars, 100 µm. **(B)** Similar percentages of Ki67^{+} cells among the Sox2^{+} cells of the SGZ in BRAF$^{+/+}$ (n = 16 images from 4 mice) and Nestin;BRAF$^{KE/+}$ (n = 14 images from 3 mice) mice (n.s., p = 0.7255 by unpaired t test). Only cells in the SGZ were counted. Data are expressed as means ± SEM.

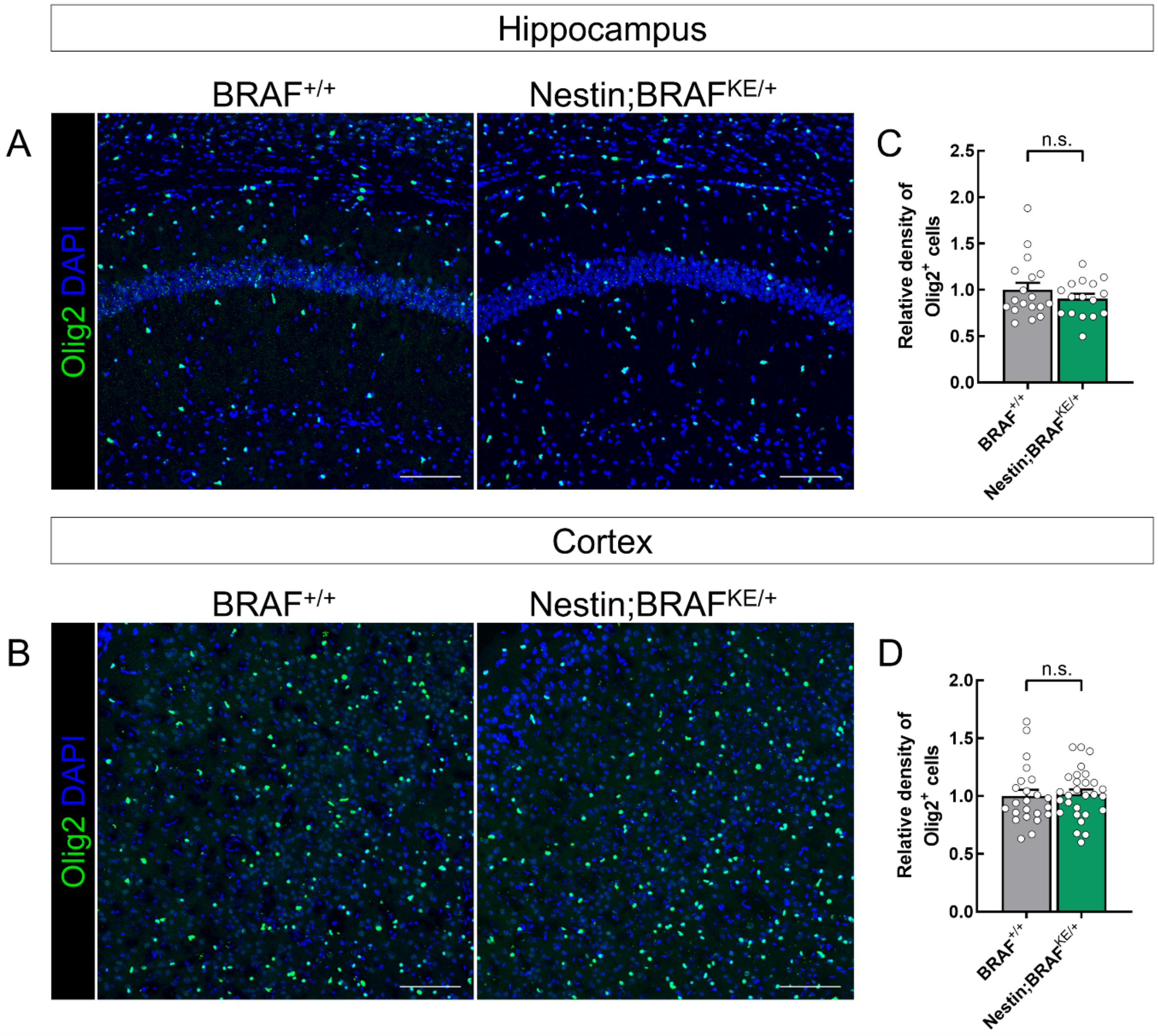

Supplemental figure 15. Nestin;BRAF$^{KE/+}$ mice do not exhibit increased Olig2-positive cells in the cortex or hippocampus. (A and B) Representative confocal images of immunolabeling for Olig2 (green) in the hippocampal CA1 and cortex of BRAF$^{+/+}$ or Nestin;BRAF$^{KE/+}$ mice. Scale bars, 100 μm. **(C)** Relative density of Olig2$^+$ cells in the hippocampal CA1 region of BRAF$^{+/+}$ (n = 18 images from 5 mice) or Nestin;BRAF$^{KE/+}$ (n = 16 images from 5 mice) mice (n.s., p = 0.3194 by unpaired t test). **(D)** Relative density of Olig2$^+$ cells in the cortex of BRAF$^{+/+}$ (n = 23 images from 5 mice) or Nestin;BRAF$^{KE/+}$ (n = 28 images from 5 mice) mice (n.s., p = 0.7973 by unpaired t test). Data are expressed as means ± SEM.

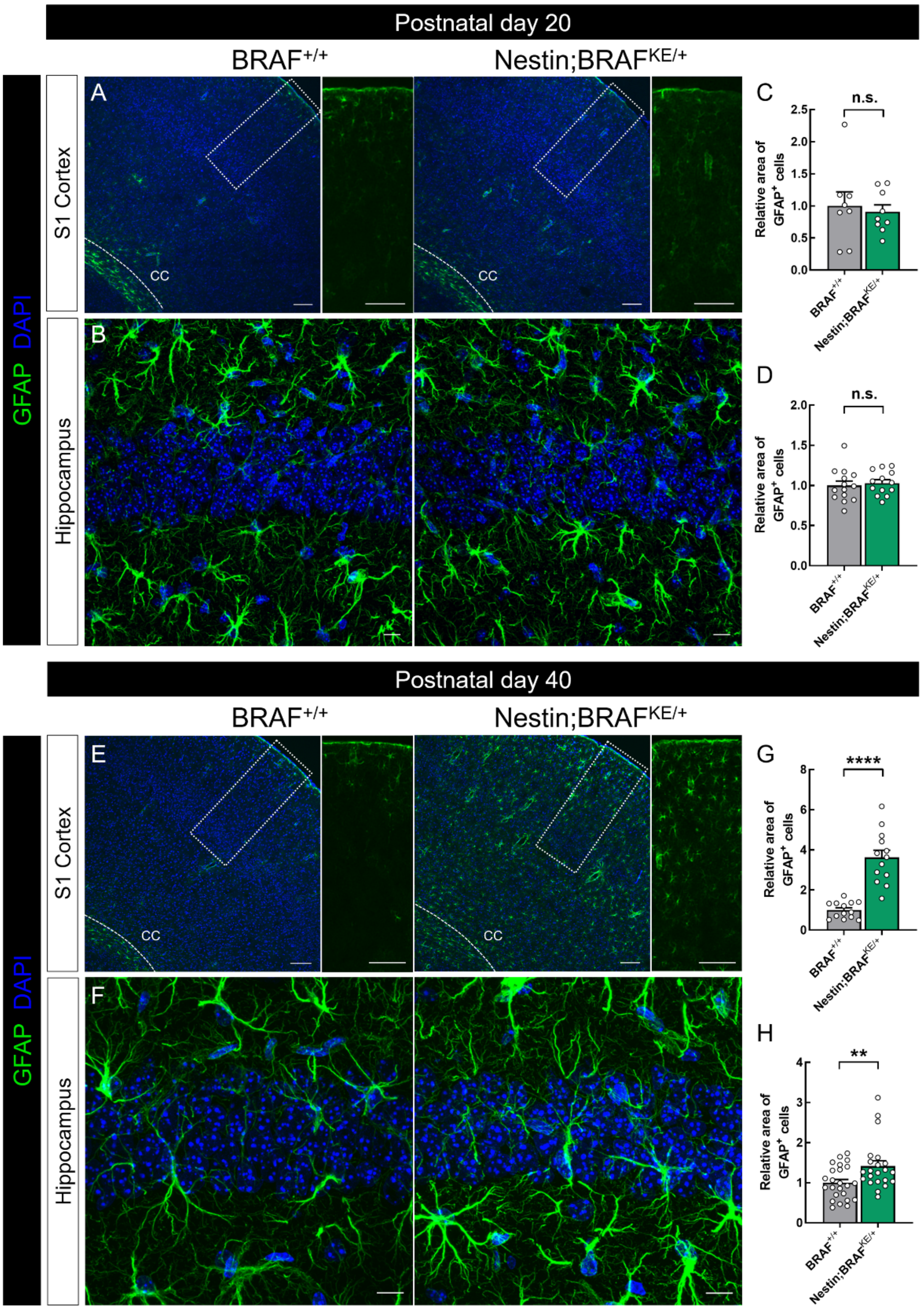

Supplemental figure 16. Nestin;BRAF$^{KE/+}$ mice exhibit increased GFAP expression at postnatal day 40 in the hippocampus. (A and B) Representative confocal images of GFAP immunolabeling in the (A) S1 cortex or (B) hippocampal CA1 region from BRAF$^{+/+}$ and Nestin;BRAF$^{KE/+}$ mice on postnatal day 20 (P20). Scale bars,

100 μm (A) or 10 μm (B). **(C and D)** Relative area of GFAP-positive cells in the (C) S1 cortex or (D) hippocampal CA1 region of mice depicted in A and B (S1 cortex: $BRAF^{+/+}$, n = 8 images from 3 mice; Nestin;$BRAF^{KE/+}$, n = 9 images from 3 mice; n.s., $p = 0.6967$ by unpaired t test. Hippocampal CA1: $BRAF^{+/+}$, n = 14 images from 3 mice; Nestin;$BRAF^{KE/+}$, n = 13 images from 3 mice; n.s., $p = 0.6648$ by unpaired t test). **(E and F)** Representative confocal images of GFAP immunolabeling in the (E) S1 cortex or (F) hippocampal CA1 region from $BRAF^{+/+}$ or Nestin;$BRAF^{KE/+}$ mice on postnatal day 40 (P40). Scale bars, 100 μm (E) or 10 μm (F). **(G and H)** Relative area of GFAP-positive cells in the (G) S1 cortex or (H) hippocampal CA1 region of mice depicted in E and F (S1 cortex: $BRAF^{+/+}$, n = 13 images from 4 mice; Nestin;$BRAF^{KE/+}$, 13 images from 4 mice; ****$p < 0.0001$ by unpaired t test. Hippocampal CA1: $BRAF^{+/+}$, n = 24 images from 4 mice; Nestin;$BRAF^{KE/+}$, n = 22 images from 4 mice; **$p < 0.01$ by unpaired t test). Data are expressed as means ± SEM.

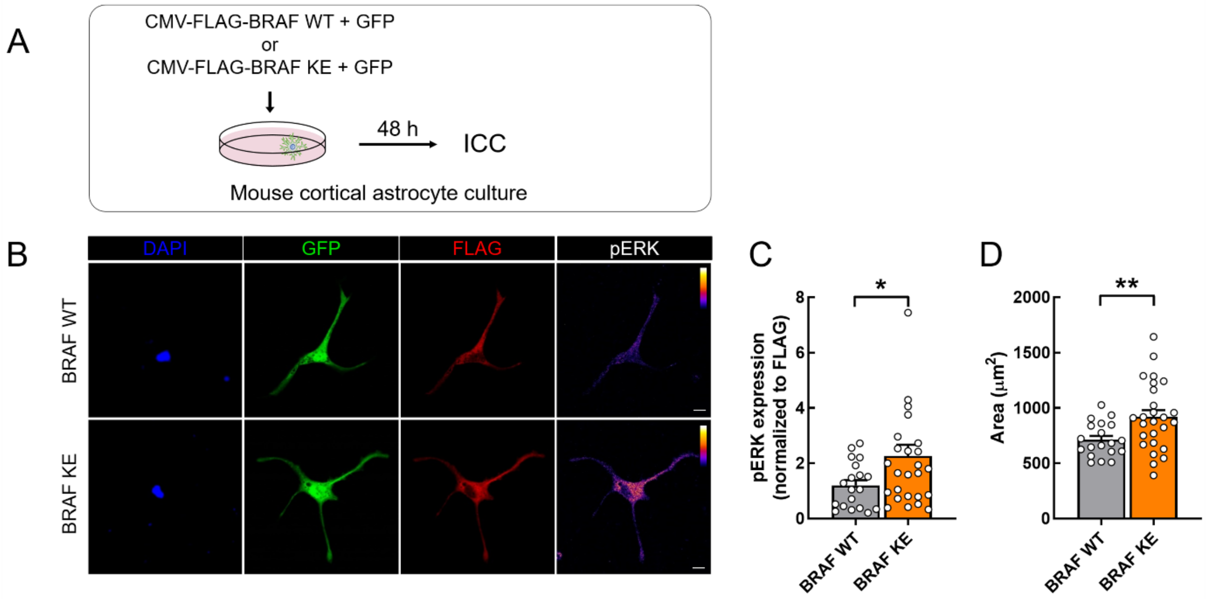

Supplemental figure 17. BRAF-mediated dysregulation of the RAS-ERK pathway induces reactive-like astrogliosis in cultured mouse cortical astrocytes. (A) Schematic diagram of the experimental design. Cultured cortical astrocytes at 10 days *in vitro* (DIV) were transfected with CMV-FLAG-BRAF WT and GFP, or CMV-FLAG-BRAF KE and GFP, cultured for 48 hours, and subsequently fixed for immunocytochemistry (ICC). **(B)** Representative ICC images for FLAG and p-ERK1/2 immunolabeling of cells expressing the CMV-FLAG-BRAF WT or CMV-FLAG-BRAF KE plasmids. **(C and D)** (C) p-ERK1/2 expression normalized to the FLAG signal or (D) astrocytic hypertrophy measured by GFP area in cultures shown in B (CMV-FLAG-BRAF WT, n = 19; CMV-FLAG-BRAF KE, n = 26; *$p < 0.05$, **$p < 0.01$ by unpaired *t* test). Data are expressed as means ± SEM.

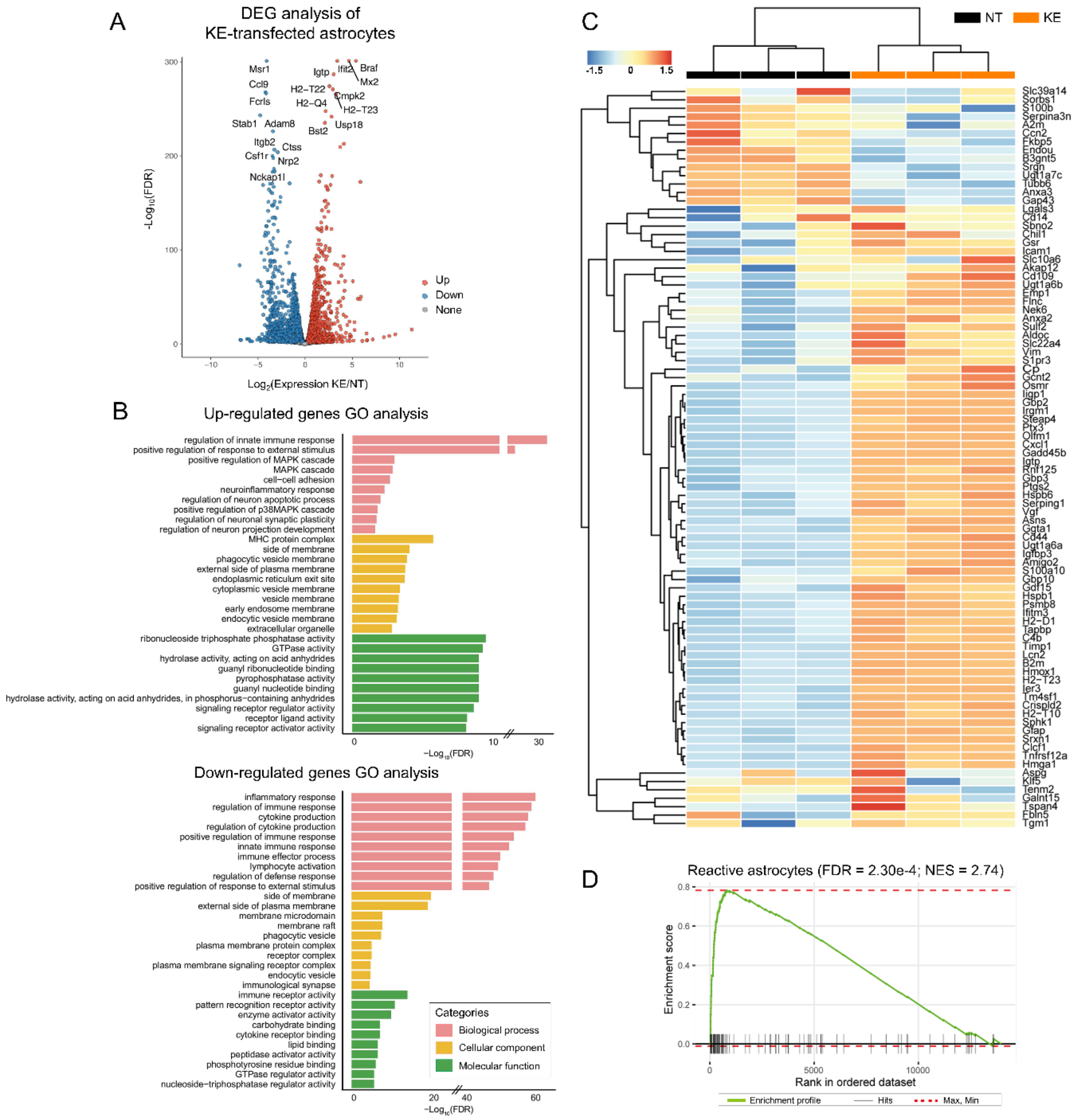

Supplemental figure 18. Transcriptomic analyses in BRAF KE-transfected primary cortical astrocytes. (A) Volcano plot depicting RNA-seq data from BRAF KE-transfected (KE) and non-transfected (NT) primary astrocytes. **(B)** Gene ontology enrichment analysis of KE and NT primary astrocytes showing the biological processes, cellular components, and molecular functions enriched in the up- and downregulated DEGs. **(C)** Hierarchical clustering plot with gene expression levels of reactive astrocyte genes represented in a heatmap using colors mapped to log_2-transformed FPKM plus 1. FPKM, fragments per kilobase of transcript per million mapped reads. **(D)** Enrichment plot of the reactive astrocyte gene set.

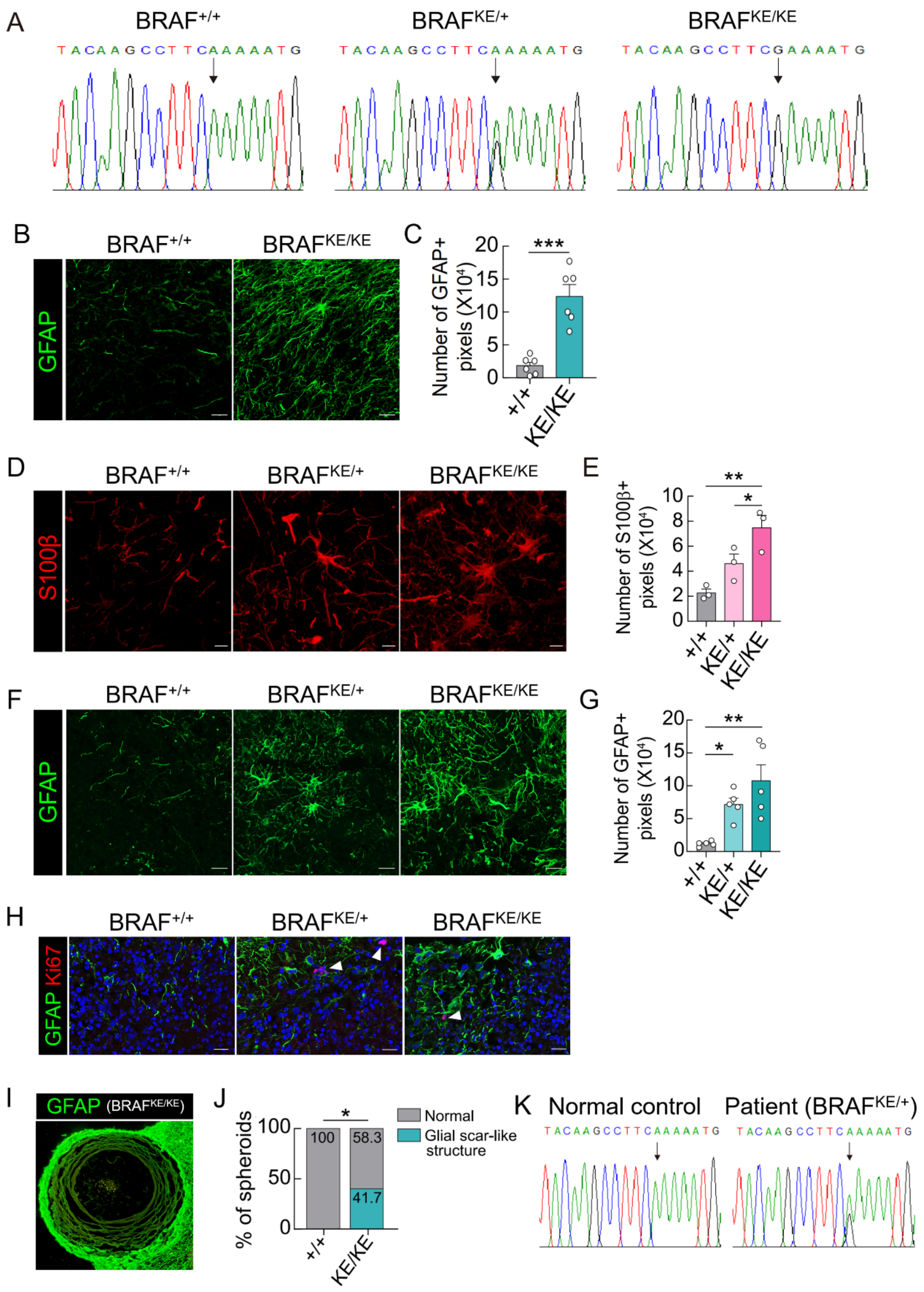

Supplemental figure 19. BRAF KE induces reactive-like astrogliosis in human cortical spheroids. (A) Sanger sequencing to confirm the presence of BRAF KE heterozygous and homozygous mutations. **(B)** Representative images of GFAP

immunolabeling in $BRAF^{+/+}$ and $BRAF^{KE/KE}$ human cortical spheroids at day 372. The same iPSC line differentiated in an independent differentiation. Scale bars, 20 μm. **(C)** Quantification of the number of GFAP-positive pixels in $BRAF^{+/+}$ and $BRAF^{KE/KE}$ human cortical spheroids (n = 6 for each genotype; ***$p < 0.001$ by unpaired Student's *t* test). **(D)** Representative images of S100β-positive cells in $BRAF^{+/+}$, $BRAF^{KE/+}$ and $BRAF^{KE/KE}$ human cortical spheroids at day 372. Scale bars, 10 μm. **(E)** Quantification of the number of S100β-positive pixels in $BRAF^{+/+}$, $BRAF^{KE/+}$ and $BRAF^{KE/KE}$ human cortical spheroids (n = 3 for each genotype; $F_{2, 6} = 12.24$, $p < 0.01$ by one-way ANOVA, Newman-Keuls multiple comparisons: **$p < 0.01$, *$p < 0.05$). **(F)** Representative images of GFAP immunolabeling in $BRAF^{+/+}$, $BRAF^{KE/+}$ and $BRAF^{KE/KE}$ human cortical spheroid differentiated from other iPSC lines at day 292. Scale bars, 20 μm. **(G)** Quantification of the number of GFAP-positive pixels in $BRAF^{+/+}$ and $BRAF^{KE/KE}$ human cortical spheroids (n = 5 for each genotype; $F_{2, 12} = 10.30$, $p < 0.01$ by one-way ANOVA, Newman-Keuls multiple comparisons: *$p < 0.05$, **$p < 0.01$). **(H)** Representative images of GFAP (green) and Ki67 (red) immunolabeling in $BRAF^{+/+}$ and $BRAF^{KE/KE}$ human cortical spheroids at day 292. Arrows indicate labeling of Ki67. Scale bars, 20 μm. **(I)** Representative image of a glial scar-like structure in $BRAF^{KE/KE}$ human cortical spheroid. **(J)** Quantification of the percentage of $BRAF^{+/+}$ and $BRAF^{KE/KE}$ human cortical spheroids with glial scar-like structures (*$p < 0.05$ by Chi-square test). **(K)** Confirmation of $BRAF^{KE/+}$ mutation in cortical spheroids from a healthy control and a RASopathy patient by Sanger sequencing. Data are expressed as means ± SEM.

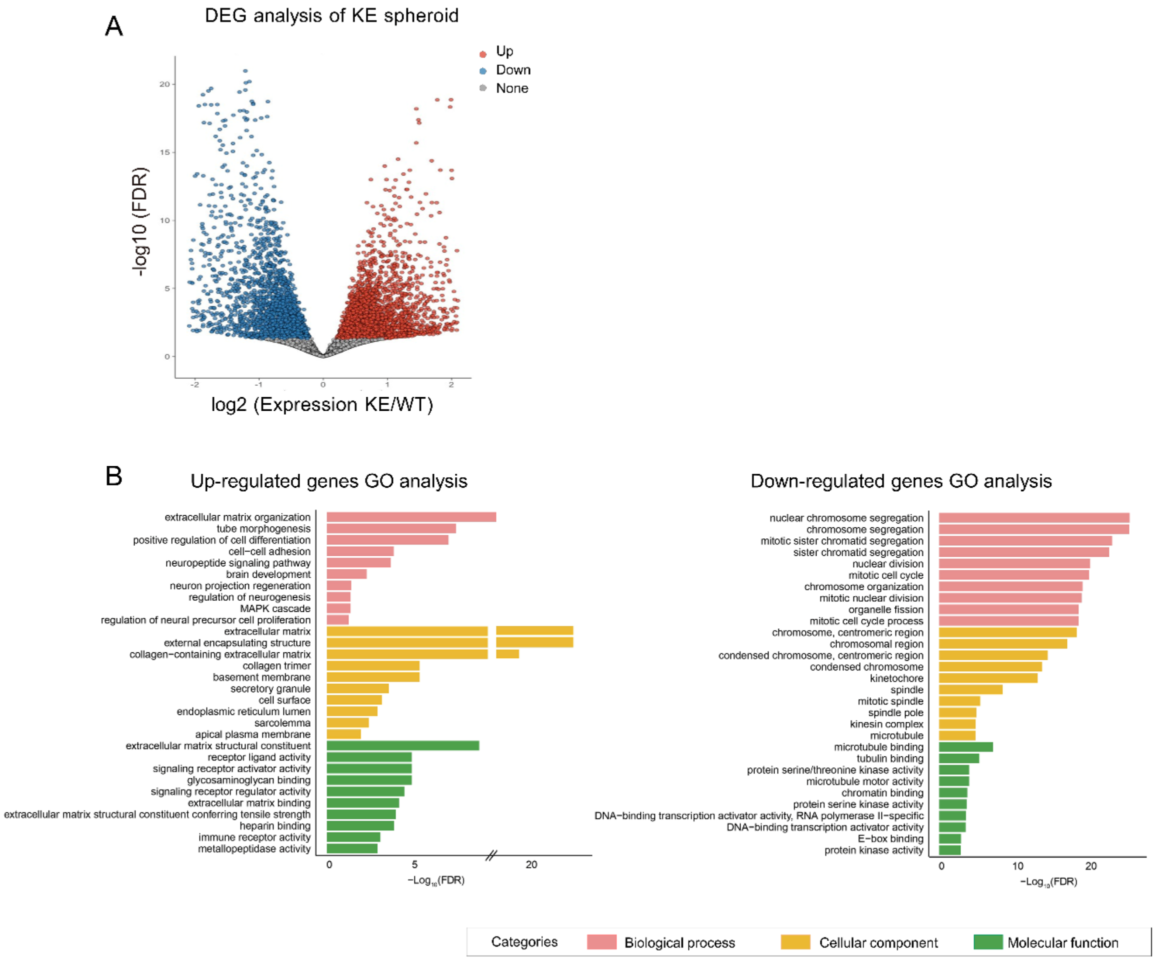

Supplemental figure 20. Transcriptomic analyses of BRAF KE human cortical spheroids. (A) Volcano plot depicting RNA-seq data from $BRAF^{+/+}$ and $BRAF^{KE/+}$ human cortical spheroids. **(B)** Gene ontology enrichment analysis of genes differentially expressed (DEGs) in $BRAF^{+/+}$ vs $BRAF^{KE/+}$ human cortical spheroids, showing various biological processes, molecular functions, and cellular components.

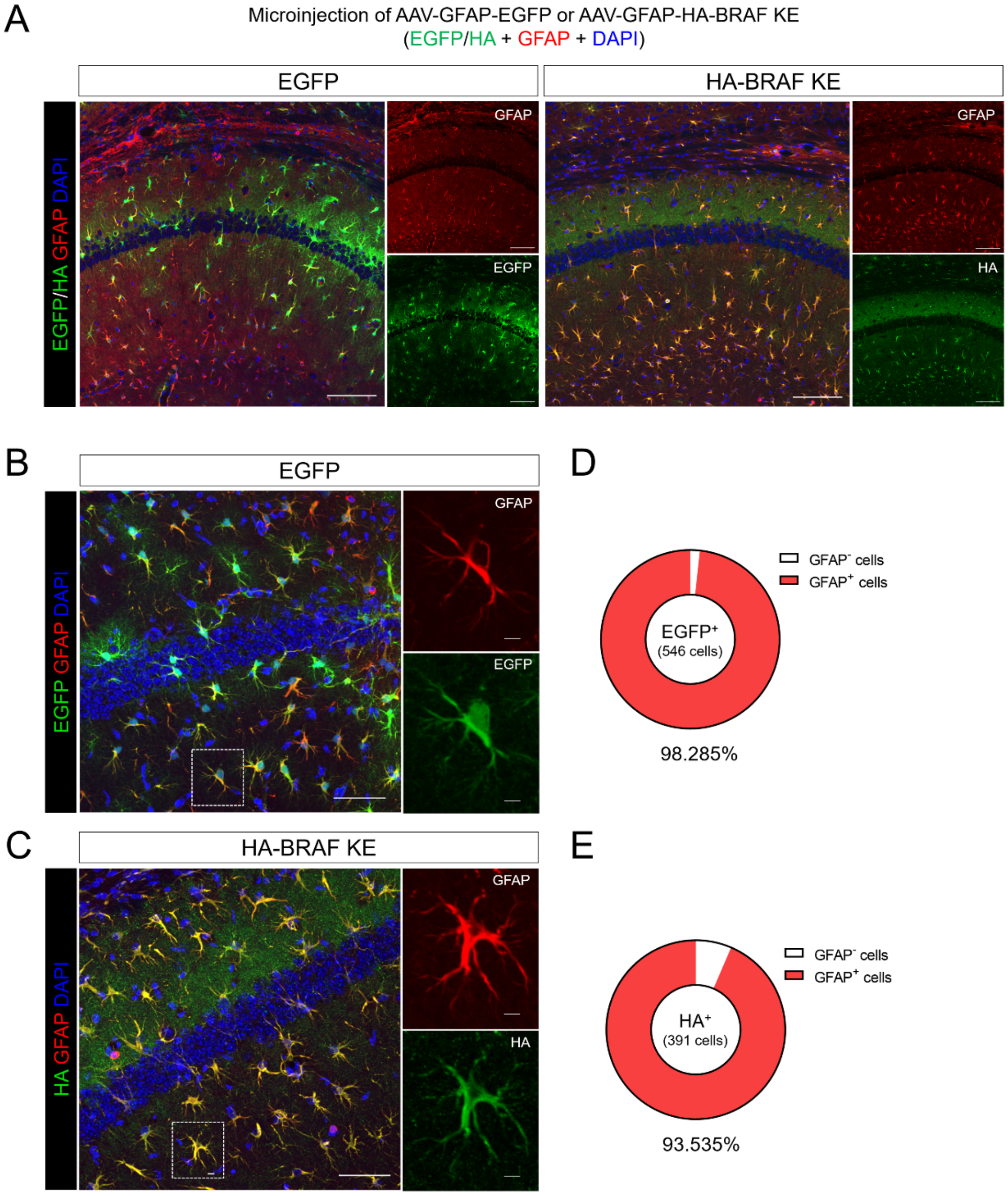

Supplemental Figure 21. Specific targeting of astrocytes by intracranial AAV microinjection. (A) Representative low magnification confocal images of GFAP (red) and HA (green) immnolabeling or EGFP (green) fluorescence in the adult hippocampus. Scale bars, 100 μm. **(B and C)** Representative high magnification confocal images of GFAP (red) and HA (green) immunolabeling and EGFP (green) fluorescence showing colocalization of GFAP and HA or EGFP. Scale bars, 50 μm, 5 μm. **(D and E)** Percentage of EGFP- or HA-positive cells that also expressed GFAP.

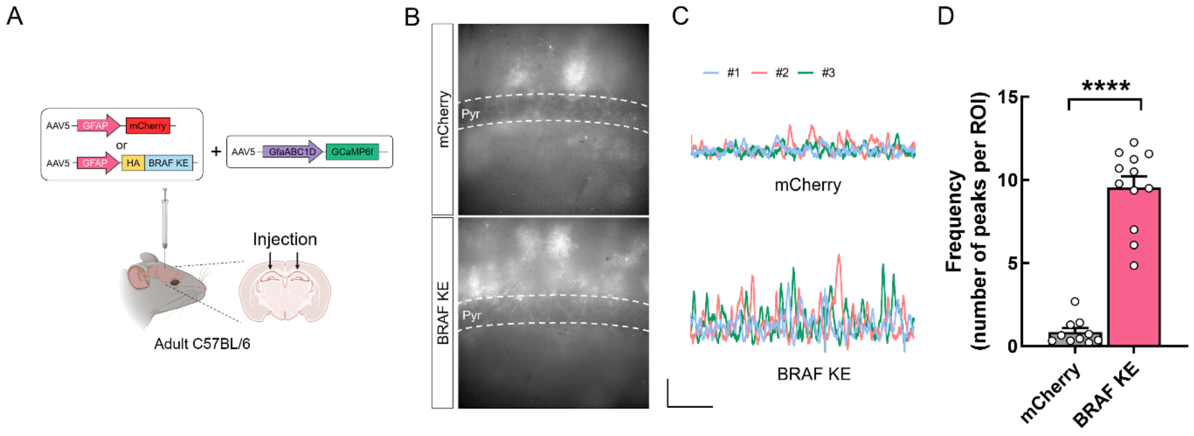

Supplemental figure 22. BRAF KE induces increased calcium fluctuations in hippocampal astrocytes. (A) Schematic illustrating the experimental approach. GFAP-mCherry or GFAP-HA-BRAF KE were injected along with a gfaABC1D-GCaMP6f virus into the hippocampal CA1 region of C57BL/6 mice. **(B)** Representative images of GCaMP6f signal in hippocampal astrocytes from mice injected with AAV-GFAP-mCherry or AAV-GFAP-BRAF KE. Pyr, pyramidal cell layer. **(C)** Representative traces showing the enhanced fluorescence of GCaMP6f in GFAP-BRAF KE-expressing astrocytes. Scale bars, 1 arbitrary unit and 1 minute for the x and y axes, respectively. **(D)** Mice injected with AAV-GFAP-BRAF KE showed a higher frequency of peaks per ROI than mice injected with AAV-GFAP-mCherry (GFAP-mCherry, n = 10 images from 5 mice; GFAP-BRAF KE, n = 12 images from 5 mice; *****p* < 0.0001 by unpaired *t* test). Data are expressed as means ± SEM.

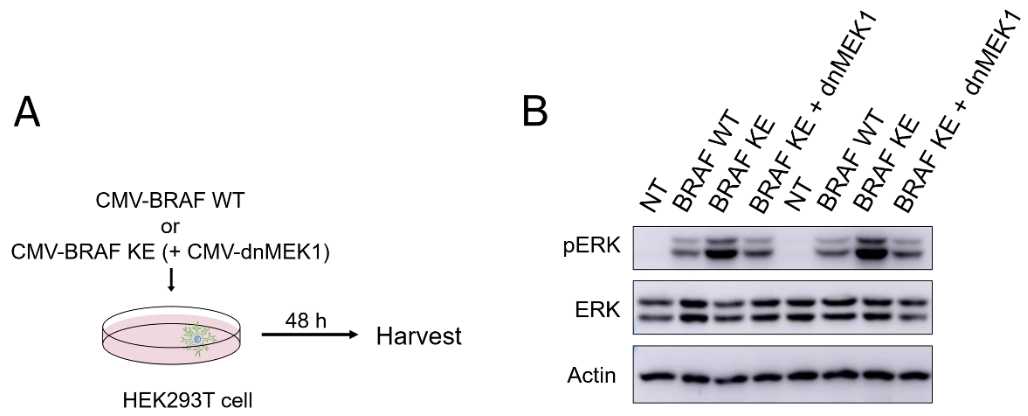

Supplemental figure 23. The effect of MEK1 K97M on the BRAF KE-induced RAS-ERK signaling overactivation. (A) The schematic diagram of experiment. HEK293T cells were transfected with CMV-BRAF WT, CMV-BRAF KE, or CMV-BRAF KE with CMV-MEK1 K97M. **(B)** Representative Western blot for p-ERK1/2, ERK1/2 and actin in cell lysates from the transfected HEK293T cells described in A. p-ERK1/2 expression was increased in BRAF KE-transfected cell lysates. Cotransfection of MEK1 K97M with BRAF KE restored BRAF KE-induced p-ERK1/2 increase. NT, not transfected.

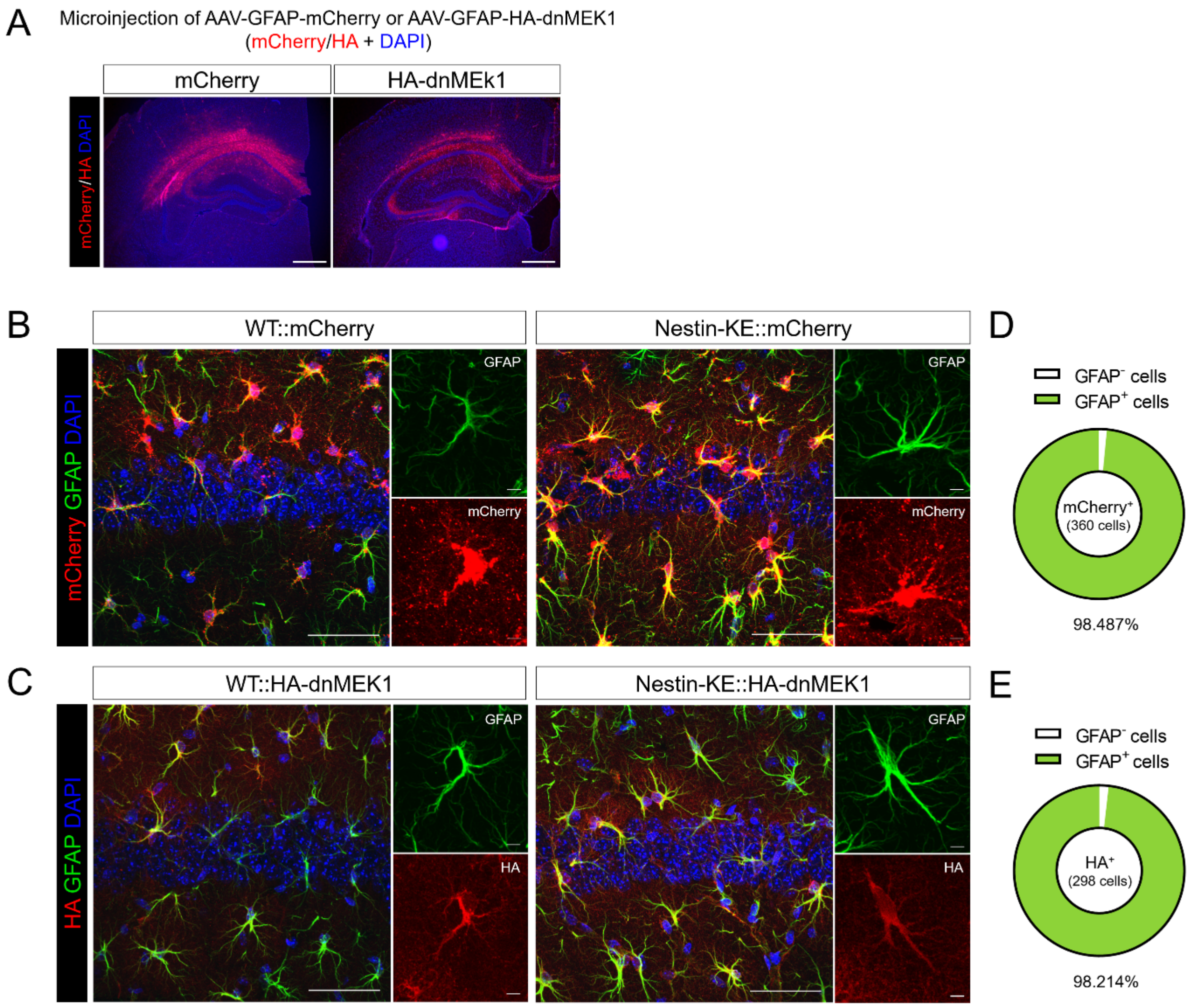

Supplemental figure 24. Characterization of GFAP-driven dnMEK1 expression in the hippocampus. (A) Representative low magnification confocal images of HA (or mCherry fluorescence, red) immunolabeling in the adult hippocampus. Scale bars, 500 μm. **(B and C)** Representative high magnification confocal images of GFAP (green) and HA (or mCherry fluorescence, red) immunolabeling showing colocalization of GFAP and HA (or mCherry). Scale bars, 50 μm, 5 μm. **(D and E)** Percentage of cells expressing mCherry or HA that also expressed GFAP (along with the cell numbers).

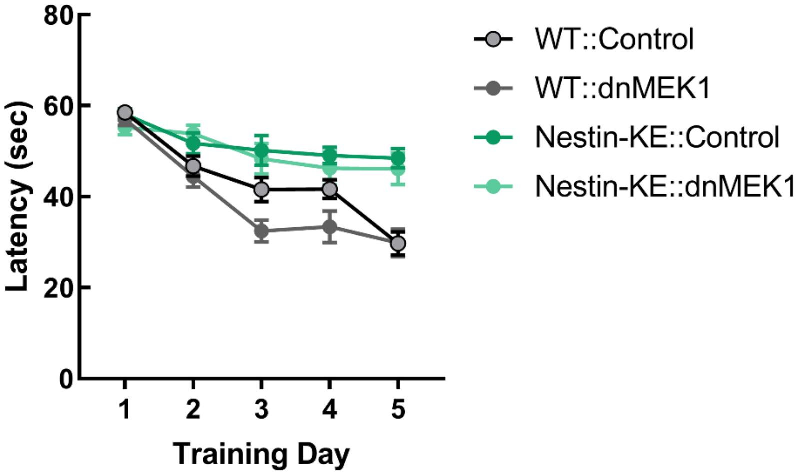

Supplemental figure 25. Morris water maze learning curves for dnMEK1-injected Nestin;BRAF$^{KE/+}$ mice. Learning curves showing the time required (latency) for WT (WT::Control, n = 19; WT::dnMEK1, n = 17) and Nestin-KE (Nestin-KE::Control, n = 13; Nestin-KE::dnMEK1, n = 13) mice to find the hidden platform during the Morris water maze on the indicated training days. Data are expressed as means ± SEM.

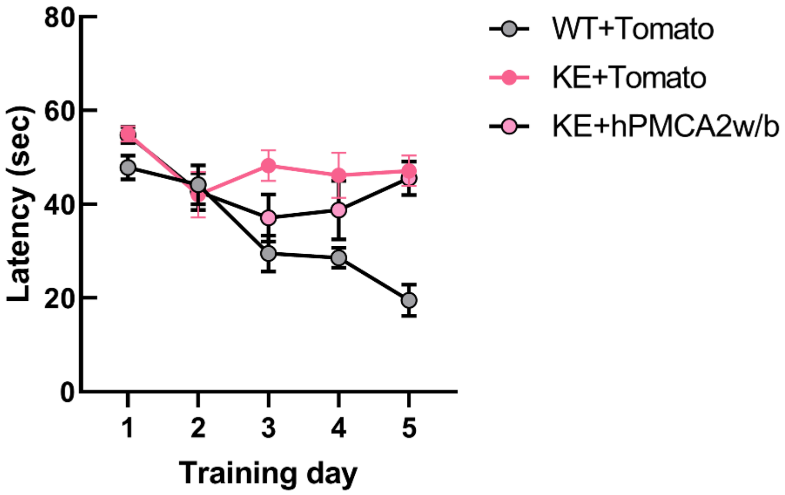

Supplemental figure 26. Learning curve during Morris water maze in hPMCA2w/b-injected BRAF KE mice. Learning curve showing the time (latency) for WT+Tomato (n = 7), KE+Tomato (n = 11), and KE+hPMCA2w/b (n = 8) mice to find the hidden platform during the Morris water maze on the indicated training days. Data are expressed as means ± SEM.

Supplemental table 1. Analysis of differentially expressed genes (DEGs) in Nestin;BRAF$^{KE/+}$ mice. (A) DEGs in Nestin;BRAF$^{KE/+}$ mice. **(B)** Gene Ontology pathways enriched for DEGs. **(C)** GSEA enriched pathways with cell type signature gene sets.

Supplemental table 2. Analysis of differentially expressed genes (DEGs) in BRAF K499E-transfected astrocytes. (A) DEGs in BRAF KE-transfected astrocyte. **(B)** Gene Ontology pathways enriched for DEGs. **(C)** GSEA enriched pathways with cell type signature gene sets.

Supplemental table 3. Analysis of differentially expressed genes (DEGs) in BRAF K499E spheroids. (A) DEGs in BRAF KE spheroids. **(B)** Gene Ontology pathways enriched for DEGs. **(C)** GSEA enriched pathways with cell type signature gene sets.

Supplemental table 4. Summary of statistical analysis, related to Figure 1 to 7.

Supplemental video 1. Astrocyte-specific expression of dominant negative MEK1 (dnMEK1) rescues increased astrocytic Ca^{2+} fluctuation in BRAF KE-injected mice. Representative videos showing that the cells infected with gfABC1D-GCaMP6f virus exhibit spontaneous Ca^{2+} fluctuation in hippocampal CA1 for 3 minutes.

Supplemental video 2. Astrocyte-specific expression of dominant negative MEK1 (dnMEK1) rescues increased astrocytic Ca^{2+} fluctuation in Nestin;BRAF$^{KE/+}$ mice. Representative videos showing that the cells infected with gfABC1D-GCaMP6f virus exhibit spontaneous Ca^{2+} fluctuation in hippocampal CA1 for 3 minutes.

Supplemental video 3. Astrocyte-specific expression of hPMCA2w/b rescues increased astrocytic Ca^{2+} fluctuation in BRAF KE-injected mice. Representative videos showing that the cells infected with gfABC1D-GCaMP6f virus exhibit spontaneous Ca^{2+} fluctuation in hippocampal CA1 for 3 minutes.
