# Supplementary material for: Aberrant ERK signaling in astrocytes impairs learning and memory in RASopathy-associated BRAF mutant mouse models
Source: J Clin Invest. 2025 Feb 18;135(8):e176631. doi: 10.1172/JCI176631 (PMC11996877; doi:10.1172/JCI176631)

Aberrant ERK signaling in astrocytes impairs learning and memory in *BRAF* mutant mouse models of RASopathy

Full blot images for Figure 4L

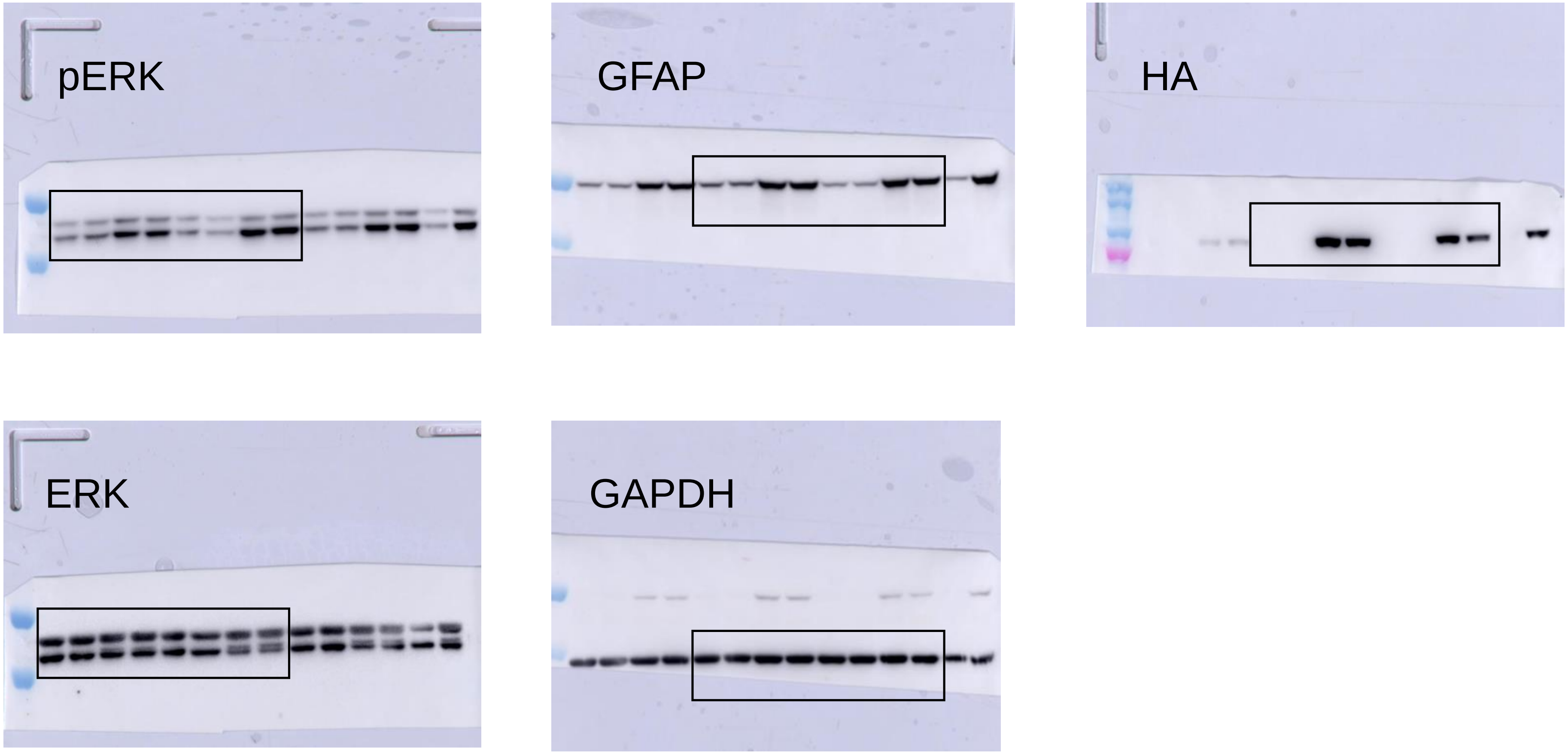

Aberrant ERK signaling in astrocytes impairs learning and memory in *BRAF* mutant mouse models of RASopathy

Full blot images for Figure 5B

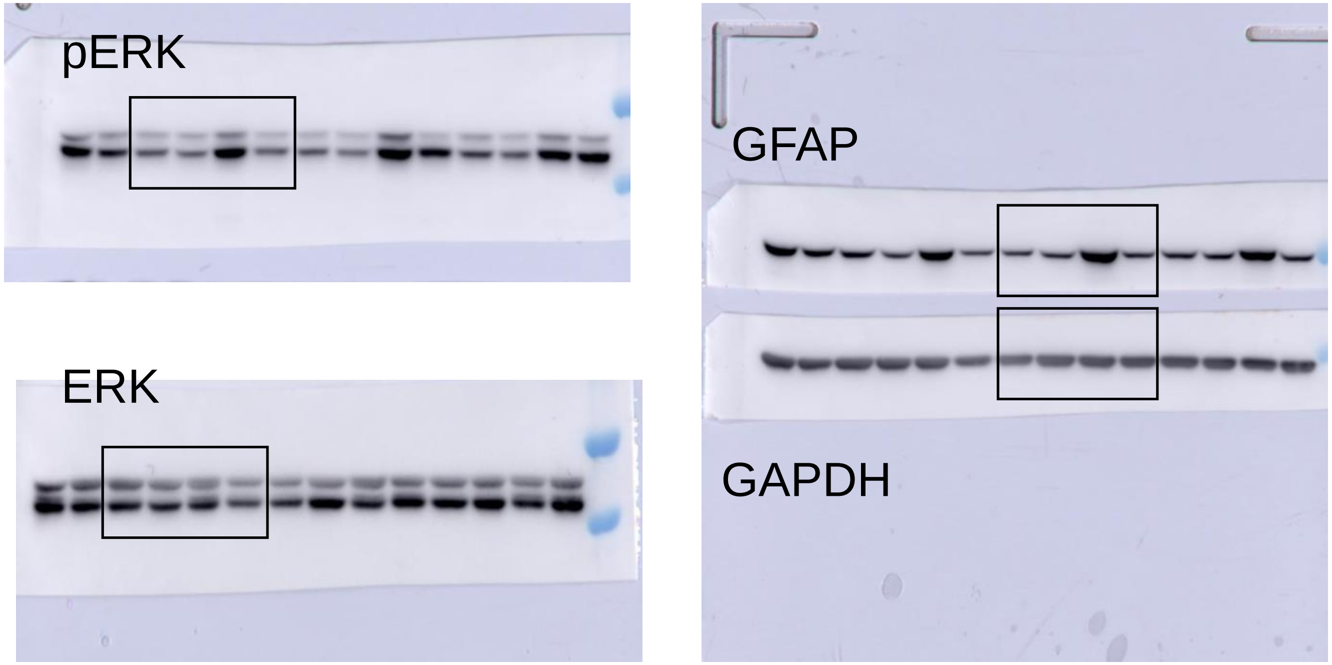

Aberrant ERK signaling in astrocytes impairs learning and memory in *BRAF* mutant mouse models of RASopathy

Full blot images for Figure 6B

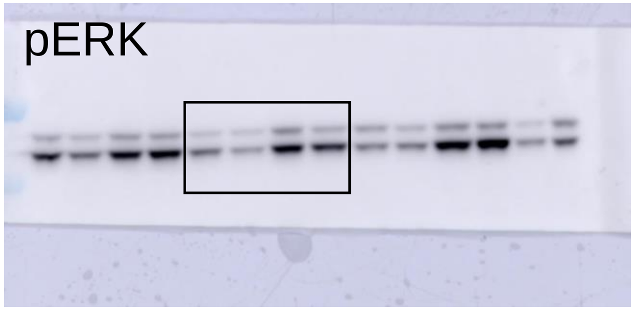

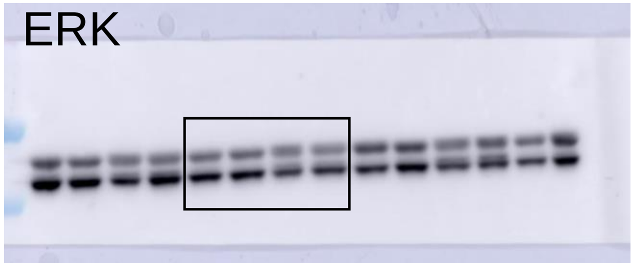

Aberrant ERK signaling in astrocytes impairs learning and memory in *BRAF* mutant mouse models of RASopathy

Full blot images for Figure 7B

pERK

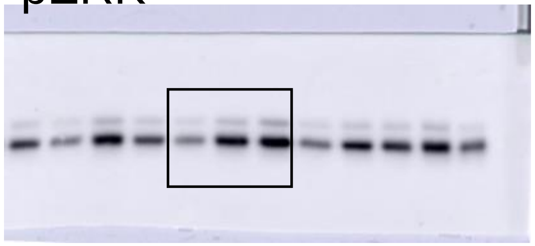

GFAP

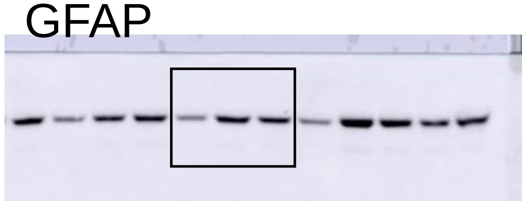

ERK

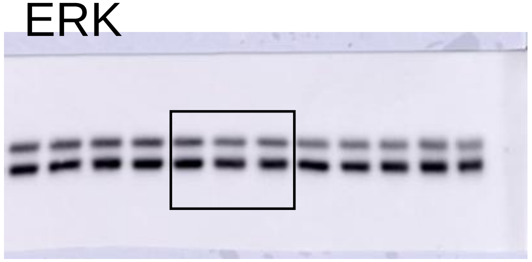

GAPDH

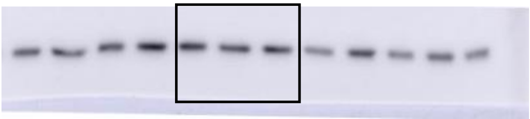

Aberrant ERK signaling in astrocytes impairs learning and memory in *BRAF* mutant mouse models of RASopathy

Full blot images for Supplemental figure 3, A-F

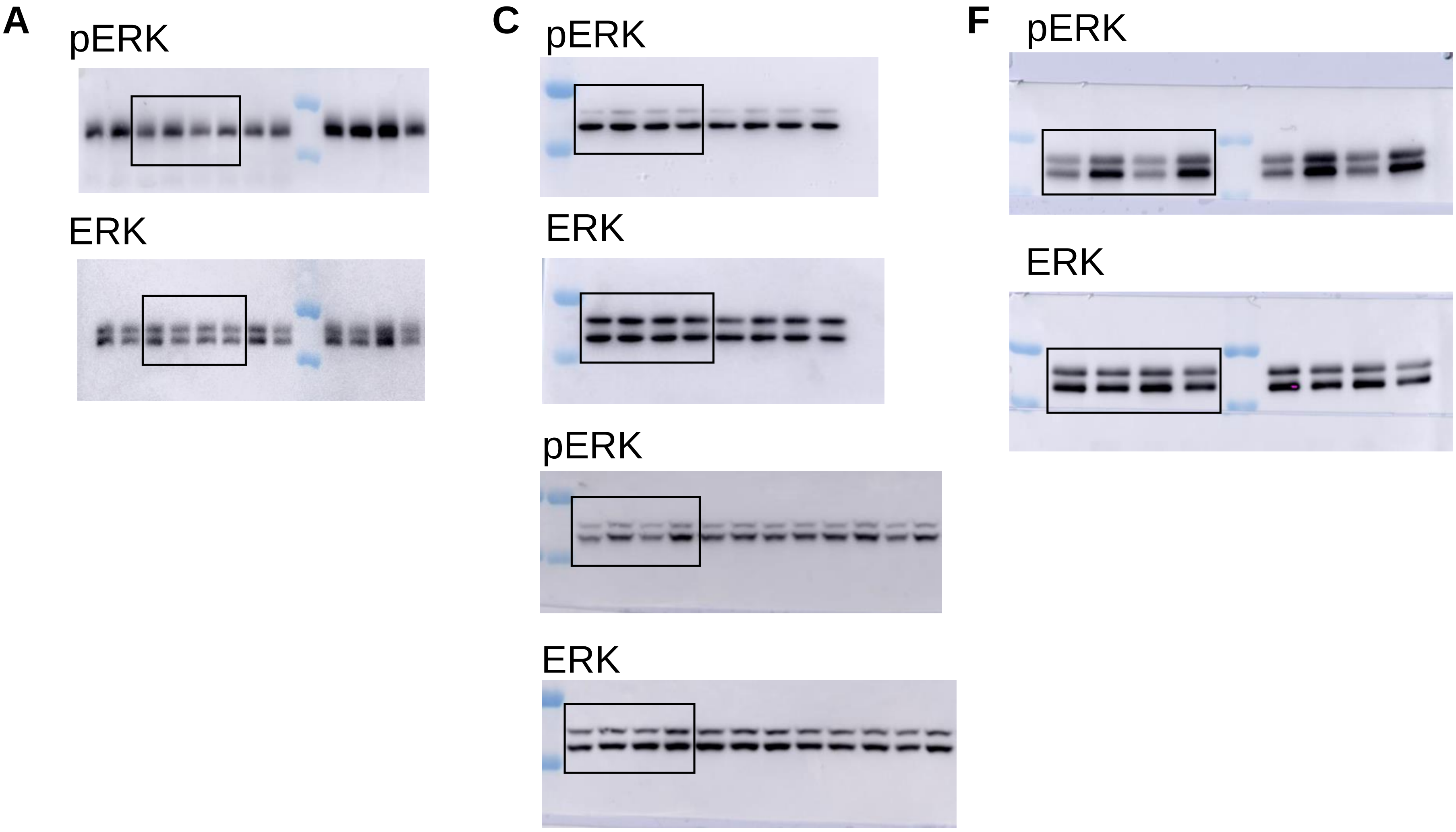

Aberrant ERK signaling in astrocytes impairs learning and memory in *BRAF* mutant mouse models of RASopathy

Full blot images for Supplemental figure 5, G and I

G

BRAF

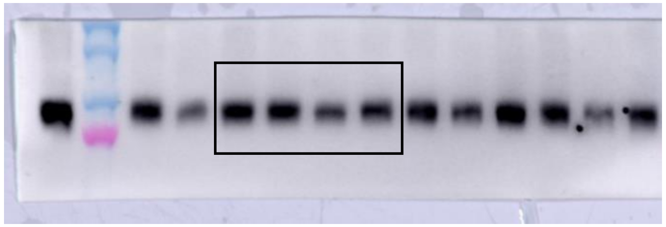

GAPDH

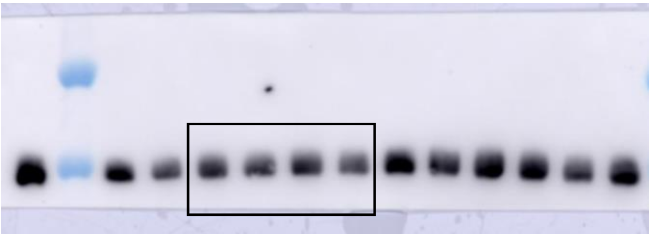

I

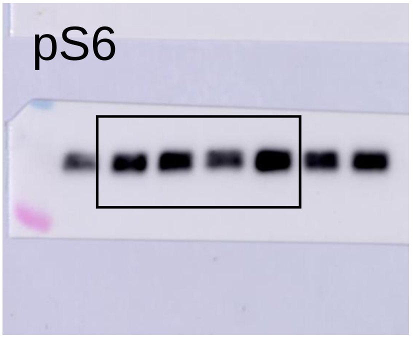

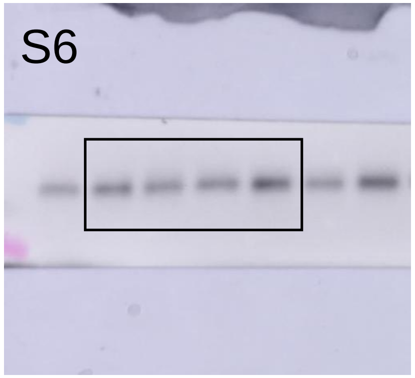

Aberrant ERK signaling in astrocytes impairs learning and memory in *BRAF* mutant mouse models of RASopathy

Full blot images for Supplemental figure 7A

GRIN1

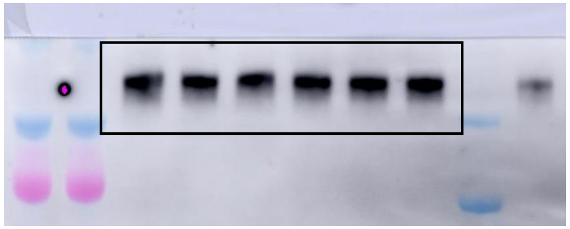

GRIA2

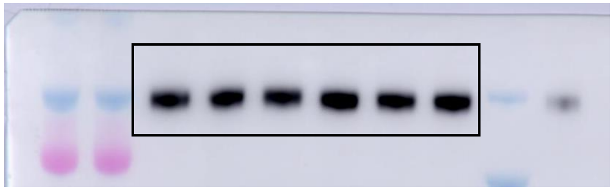

gephyrin

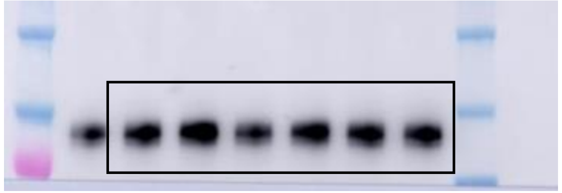

GRIN2A

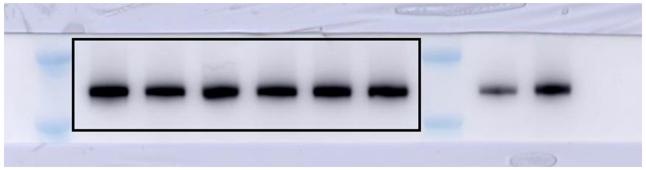

synapsin

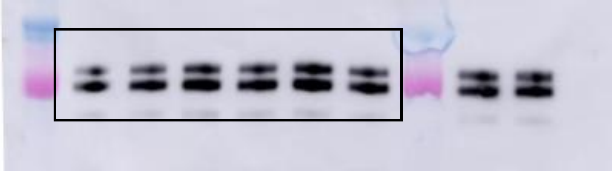

vGAT

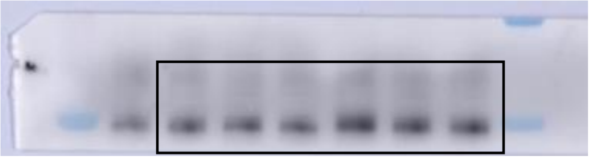

GRIN2B

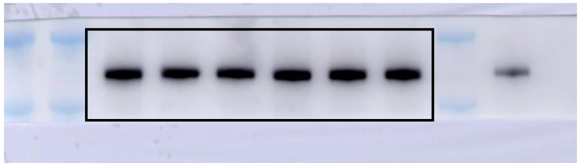

vGLUT1

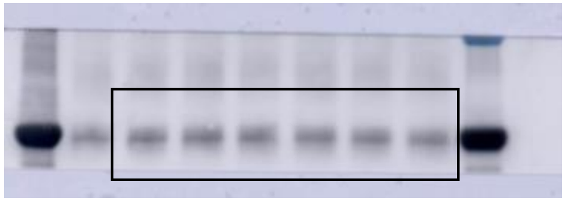

Actin

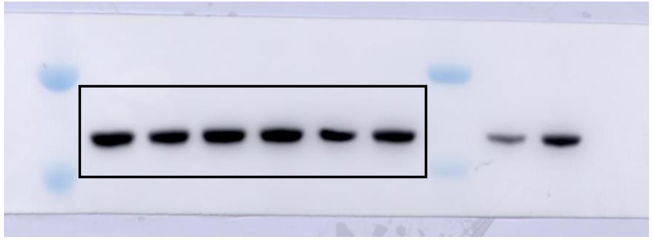

GRIA1

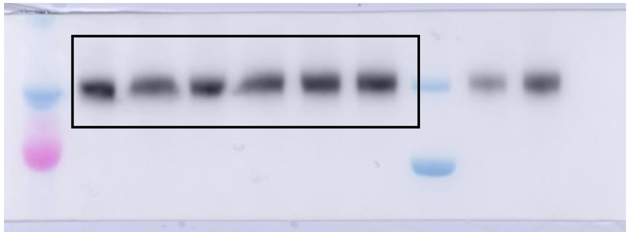

PSD95

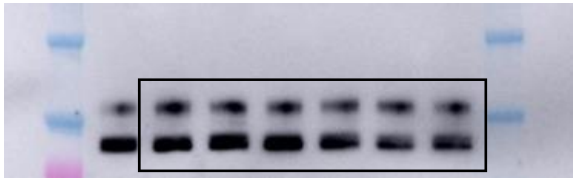

Aberrant ERK signaling in astrocytes impairs learning and memory in *BRAF* mutant mouse models of RASopathy

Full blot images for Supplemental figure 7B

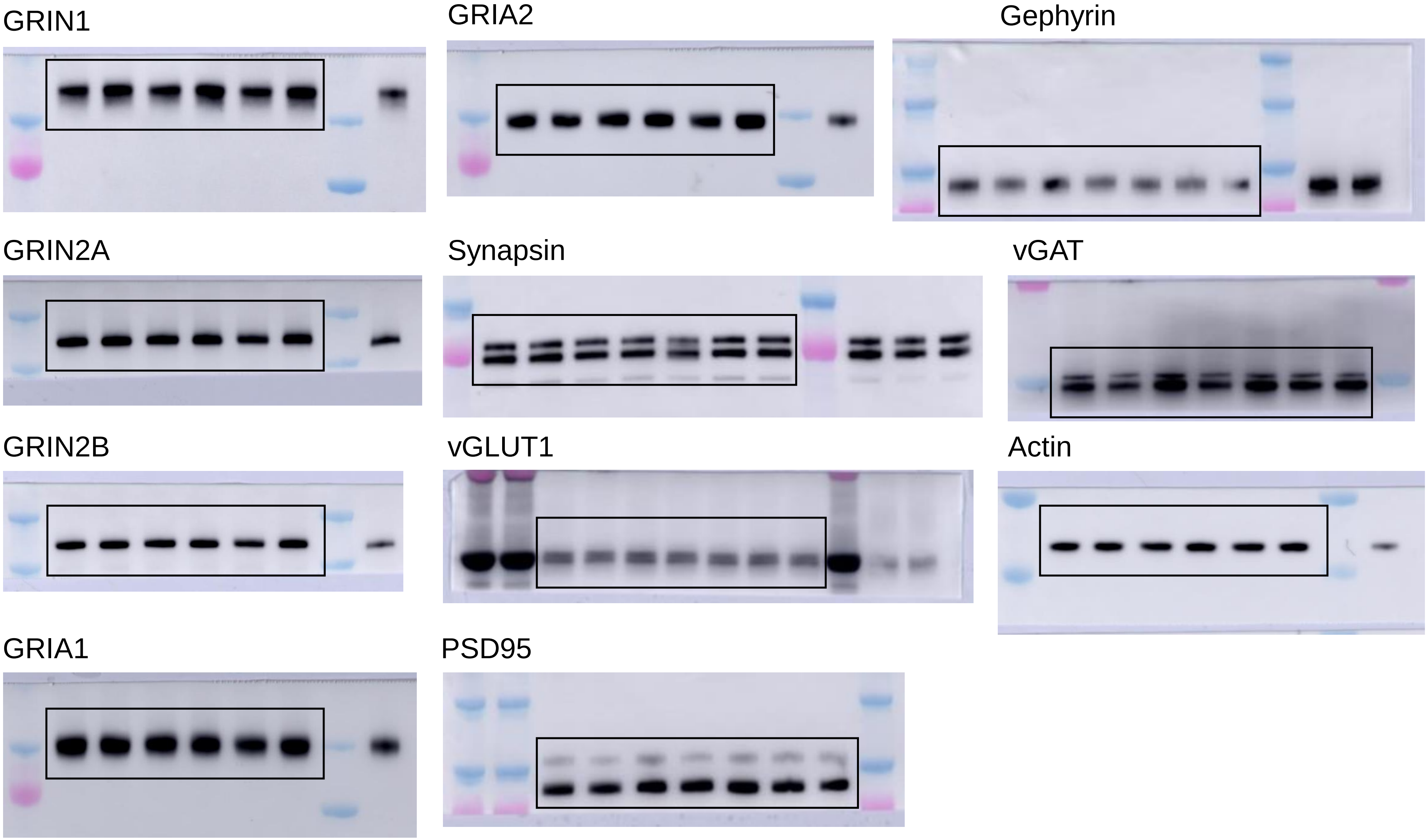

Aberrant ERK signaling in astrocytes impairs learning and memory in *BRAF* mutant mouse models of RASopathy

Full blot images for Supplemental figure 10F

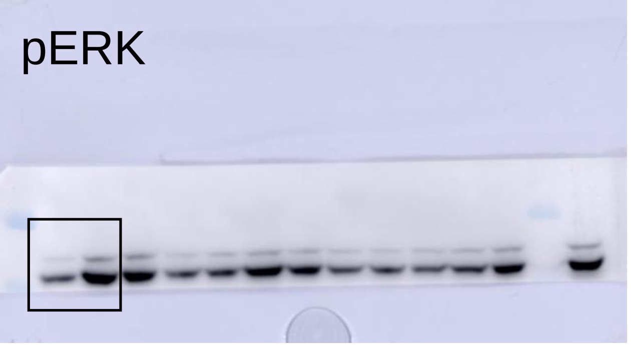

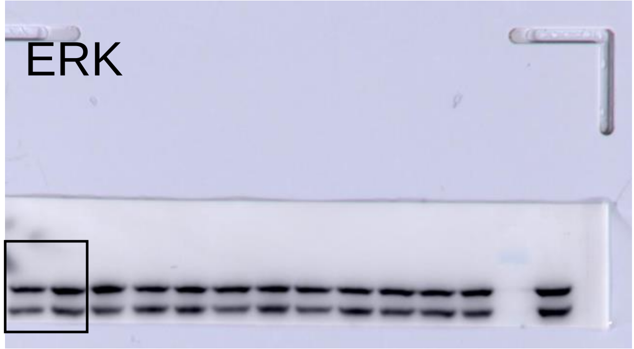

Aberrant ERK signaling in astrocytes impairs learning and memory in *BRAF* mutant mouse models of RASopathy

Full blot images for Supplemental figure 23B

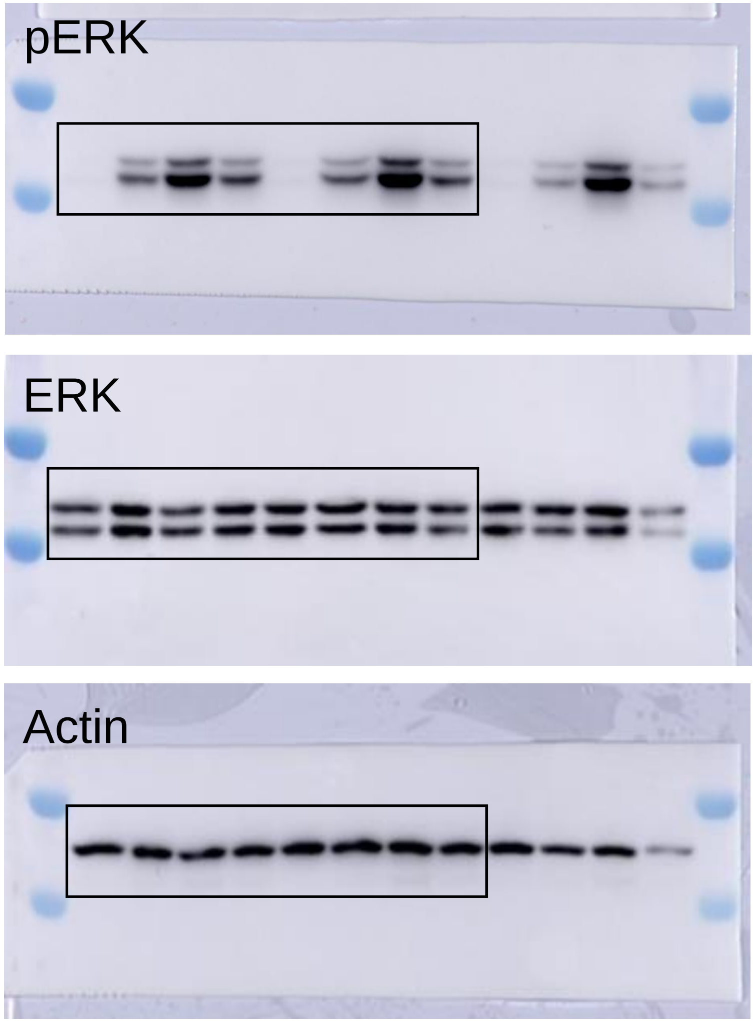

Supplement: Unedited blot and gel images [file jci-135-176631-s124.pdf]
