# Supplementary material for: Aberrant ERK signaling in astrocytes impairs learning and memory in RASopathy-associated BRAF mutant mouse models
Source: J Clin Invest. 2025 Feb 18;135(8):e176631. doi: 10.1172/JCI176631 (PMC11996877; doi:10.1172/JCI176631)
Supplement: Supplemental table 4 [file jci-135-176631-s128.pdf]

Supplemental table 4. Summary of statistical analysis, related to Figure 1 to 7.

Figure	Sample size (n)	Analysis	P value	Degrees of freedom & F value
1E	Control ($BRAF^{+/+}$, $BRAF^{KE\ floxed/+}$, $\alpha CaMKII^{+}$) = 11<br>αCaMKII;$BRAF^{KE/+}$ = 10	One-way ANOVA	****$p < 0.0001$ for control<br>****$p < 0.0001$ for αCaMKII;$BRAF^{KE/+}$	$F_{3, 40} = 10.81$<br>$F_{3, 36} = 24.89$
1F	Control ($BRAF^{+/+}$, $BRAF^{KE\ floxed/+}$, $\alpha CaMKII^{+}$) = 11<br>αCaMKII;$BRAF^{KE/+}$ = 10	Unpaired *t* test	n.s., $p = 0.1960$	
1I	Control ($BRAF^{+/+}$, $BRAF^{KE\ floxed/+}$, $vGAT^{+}$) = 15<br>vGAT;$BRAF^{KE/+}$ = 5	One-way ANOVA	****$p < 0.0001$ for control<br>**$p < 0.01$ for vGAT;$BRAF^{KE/+}$	$F_{3, 56} = 14.75$<br>$F_{3, 16} = 6.306$
1J	Control ($BRAF^{+/+}$, $BRAF^{KE\ floxed/+}$, $vGAT^{+}$) = 15<br>vGAT;$BRAF^{KE/+}$ = 5	Unpaired *t* test	n.s., $p = 0.9273$	
1L	Control ($BRAF^{+/+}$, $BRAF^{KE\ floxed/+}$, $\alpha CaMKII^{+}$) = 12<br>αCaMKII;$BRAF^{KE/+}$ = 13	Unpaired *t* test	n.s., $p = 0.9140$	
1M	Control ($BRAF^{+/+}$, $BRAF^{KE\ floxed/+}$, $vGAT^{+}$) = 14<br>vGAT;$BRAF^{KE/+}$ = 5	Unpaired *t* test	n.s., $p = 0.2300$	
1N	Control ($BRAF^{+/+}$) = 10<br>Nestin;$BRAF^{KE/+}$ = 8	Two-way ANOVA	Effect of genotype, ****$p < 0.0001$	$F_{1, 16} = 36.61$
1P	Control ($BRAF^{+/+}$) = 10<br>Nestin;$BRAF^{KE/+}$ = 8	One-way ANOVA	***$p < 0.001$ for control<br>n.s., $p = 0.8149$ for Nestin;$BRAF^{KE/+}$	$F_{3, 36} = 7.743$<br>$F_{3, 28} = 0.3142$
		Unpaired *t* test	*$p < 0.05$	
1Q	Control ($BRAF^{+/+}$) = 10<br>Nestin;$BRAF^{KE/+}$ = 8	Unpaired *t* test	**$p < 0.01$	
1R	Control ($BRAF^{+/+}$) = 19<br>Nestin;$BRAF^{KE/+}$ = 15	Unpaired *t* test	*$p < 0.05$	
1T	Control ($BRAF^{+/+}$) = 10 slices from 5 mice<br>Nestin;$BRAF^{KE/+}$ = 9 slices from 5 mice	Two-way ANOVA	Effect of genotype, n.s., $p = 0.5494$	$F_{1, 17} = 0.3732$
1U	Control ($BRAF^{+/+}$) = 12 slices from 7 mice<br>Nestin;$BRAF^{KE/+}$ = 13 slices from 7 mice	Two-way ANOVA	Effect of genotype, ****$p < 0.0001$	$F_{1, 23} = 22.54$

1V	Control ($BRAF^{+/+}$) = 12 slices from 7 mice<br>Nestin;$BRAF^{KE/+}$ = 13 slices from 7 mice	Unpaired *t* test	****$p < 0.0001$	
2B	$BRAF^{+/+}$ = 6 images from 3 mice<br>Nestin;$BRAF^{KE/+}$ = 6 images from 3 mice	Unpaired *t* test	*$p < 0.05$	
2C	$BRAF^{+/+}$ = 35 images from 8 mice<br>Nestin;$BRAF^{KE/+}$ = 32 images from 7 mice	Unpaired *t* test	n.s., $p = 0.1122$	
2E	$BRAF^{+/+}$ = 20 images from 5 mice<br>Nestin;$BRAF^{KE/+}$ = 17 images from 5 mice	Unpaired *t* test	**$p < 0.01$	
2F	$BRAF^{+/+}$ = 26 images from 7 mice<br>Nestin;$BRAF^{KE/+}$ = 23 images from 7 mice	Unpaired *t* test	****$p < 0.0001$	
2H	$BRAF^{+/+}$ = 12 cells from 3 mice<br>Nestin;$BRAF^{KE/+}$ = 12 cells from 3 mice	Two-way ANOVA	Effect of genotype, ****$p < 0.0001$	$F_{1, 924} = 209.7$
2I	$BRAF^{+/+}$ = 12 cells from 3 mice<br>Nestin;$BRAF^{KE/+}$ = 12 cells from 3 mice	Unpaired *t* test	***$p < 0.001$	
2K	$BRAF^{+/+}$ = 5<br>Nestin;$BRAF^{KE/+}$ = 4	Unpaired *t* test	**$p < 0.01$, ***$p < 0.001$, ****$p < 0.0001$	
3C	$BRAF^{+/+}$ = 3<br>$BRAF^{KE/+}$ = 3<br>$BRAF^{KE/KE}$ = 3	One-way ANOVA	$p < 0.01$<br>Newman-Keuls multiple comparisons:<br>*$p < 0.05$, **$p < 0.01$, ***$p < 0.001$	$F_{2, 6} = 26.56$
3E	$BRAF^{+/+}$ = 3<br>$BRAF^{KE/+}$ = 3<br>$BRAF^{KE/KE}$ = 3	One-way ANOVA	$p < 0.001$<br>Newman-Keuls multiple comparisons:<br>*$p < 0.05$, **$p < 0.01$, ***$p < 0.001$	$F_{2, 6} = 27.64$
3F	$BRAF^{+/+}$ = 3<br>$BRAF^{KE/+}$ = 3<br>$BRAF^{KE/KE}$ = 3	One-way ANOVA	$p < 0.01$<br>Newman-Keuls multiple comparisons:<br>*$p < 0.05$, **$p < 0.01$, ***$p < 0.001$	$F_{2, 6} = 18.14$
3H	$BRAF^{+/+}$ = 3<br>$BRAF^{KE/+}$ = 3	Unpaired *t* test	*$p < 0.05$, **$p < 0.01$, ***$p < 0.001$	
3L	Control = 9<br>RASopathy patient = 8	Unpaired *t* test	**$p < 0.01$	
4B	GFP = 11<br>BRAF KE = 9	Two-way ANOVA	Effect of genotype, *$p < 0.05$	$F_{1, 18} = 5.997$
4D	GFP = 11<br>BRAF KE = 9	One-way ANOVA	***$p < 0.001$ for EGFP<br>n.s., $p = 0.4875$ for BRAF KE	$F_{3, 40} = 0.5299$<br>$F_{3, 32} = 0.1375$
4E	GFP = 11<br>BRAF KE = 9	Unpaired *t* test	**$p < 0.01$	
4F	GFP = 11<br>BRAF KE = 9	Unpaired *t* test	**$p < 0.01$	

4H	GFP = 8 images from 3 mice<br>BRAF KE = 7 images from 3 mice	Unpaired *t* test	****$p < 0.0001$	
4J	GFP = 16 cells from 3 mice<br>BRAF KE = 14 cells from 3 mice	Two-way ANOVA	Effect of genotype, ****$p < 0.0001$	$F_{1, 1400} = 487.3$
4K	GFP = 16 cells from 3 mice<br>BRAF KE = 14 cells from 3 mice	Unpaired *t* test	****$p < 0.0001$	
4M	GFP = 7<br>BRAF KE = 7	Unpaired *t* test	****$p < 0.0001$	
4N	GFP = 7<br>BRAF KE = 7	Unpaired *t* test	****$p < 0.0001$	
5C	WT + saline = 7<br>WT + dnMEK1 = 7<br>KE + saline = 9<br>KE + dnMEK1 = 8	Two-way ANOVA	Effect of genotype, $p < 0.0001$<br>Effect of treatment, $p < 0.01$<br>Bonferroni multiple comparisons:<br>****$p < 0.0001$, ***$p < 0.001$	$F_{1, 27} = 64.37$<br>$F_{1, 27} = 12.66$
5D	WT + saline = 7<br>WT + dnMEK1 = 7<br>KE + saline = 9<br>KE + dnMEK1 = 8	Two-way ANOVA	Effect of genotype, $p < 0.0001$<br>Effect of treatment, $p < 0.0001$<br>Bonferroni multiple comparisons:<br>****$p < 0.0001$	$F_{1, 27} = 28.36$<br>$F_{1, 27} = 28.27$
5G	WT + saline = 16 images from 4 mice<br>WT + dnMEK1 = 16 images from 4 mice<br>KE + saline = 21 images from 5 mice<br>KE + dnMEK1 = 14 images from 4 mice	Two-way ANOVA	Effect of genotype, $p < 0.0001$<br>Effect of treatment, $p < 0.01$<br>Bonferroni multiple comparisons:<br>****$p < 0.0001$, ***$p < 0.001$	$F_{1, 63} = 32.67$<br>$F_{1, 63} = 10.81$
6C	WT::Saline = 12<br>WT::dnMEK1 = 10<br>Nestin-KE::Saline = 8<br>Nestin-KE::dnMEK1 = 9	Two-way ANOVA	Effect of genotype, $p < 0.0001$<br>Effect of treatment, $p < 0.05$<br>Bonferroni multiple comparisons:<br>****$p < 0.0001$, **$p < 0.01$	$F_{1, 35} = 164.0$,<br>$F_{1, 35} = 7.327$
6F	WT::Saline = 11 images from 4 mice<br>WT::dnMEK1 = 9 images from 3 mice<br>Nestin-KE::Saline = 14 images from 4 mice<br>Nestin-KE::dnMEK1 = 19 images from 5 mice	Two-way ANOVA	Effect of genotype, $p < 0.001$<br>Effect of treatment, $p < 0.001$<br>Bonferroni multiple comparisons:<br>**$p < 0.01$, ***$p < 0.001$	$F_{1, 49} = 14.76$<br>$F_{1, 49} = 15.21$
6H	WT::Control = 19<br>WT::dnMEK1 = 17<br>Nestin-KE::Control = 13<br>Nestin-KE::dnMEK1 = 13	One-way ANOVA	****$p < 0.0001$ for WT::Control<br>n.s., $p = 0.2842$ for WT::dnMEK1<br>n.s., $p = 0.6898$ for Nestin-KE::Control<br>***$p < 0.001$ for Nestin-KE::dnMEk1	$F_{3, 72} = 12.99$<br>$F_{3, 48} = 6.589$<br>$F_{3, 48} = 0.4916$<br>$F_{3, 64} = 1.294$

6I	WT::Control = 19<br>WT::dnMEK1 = 17<br>Nestin-KE::Control = 13<br>Nestin-KE::dnMEK1 = 13	Two-way ANOVA	Effect of genotype, $p < 0.0001$<br>Effect of treatment, $p = 0.2234$<br>Bonferroni multiple comparisons:<br>****$p < 0.0001$, **$p < 0.01$	$F_{1, 58} = 18.32$<br>$F_{1, 58} = 1.514$
6J	WT::Control = 19<br>WT::dnMEK1 = 17<br>Nestin-KE::Control = 13<br>Nestin-KE::dnMEK1 = 13	Two-way ANOVA	Effect of genotype, $p < 0.05$<br>Effect of treatment, $p = 0.0652$<br>Bonferroni multiple comparisons:<br>*$p < 0.05$	$F_{1, 58} = 4.382$<br>$F_{1, 58} = 3.532$
7C	WT + Tomato = 10<br>KE + Tomato = 13<br>KE + hPMCA2w/b = 15	One-way ANOVA	$p < 0.0001$<br>Bonferroni multiple comparisons:<br>****$p < 0.0001$, n.s., $p = 0.1347$	$F_{2, 35} = 20.35$
7D	WT + Tomato = 10<br>KE + Tomato = 13<br>KE + hPMCA2w/b = 15	One-way ANOVA	$p < 0.01$<br>Bonferroni multiple comparisons:<br>**$p < 0.01$, n.s., $p > 0.9999$	$F_{2, 35} = 7.085$
7G	WT + Tomato = 14 images from 4 mice<br>WT + hPMCA2w/b = 8 images from 2 mice<br>KE + Tomato = 13 images from 4 mice<br>KE + hPMCA2w/b = 18 images from 4 mice	Two-way ANOVA	Effect of genotype, $p < 0.01$<br>Effect of treatment, $p < 0.0001$<br>Bonferroni multiple comparisons:<br>****$p < 0.0001$	$F_{1, 49} = 10.50$<br>$F_{1, 49} = 34.75$
7I	WT + Tomato = 7<br>KE + Tomato = 11<br>KE + hPMCA2w/b = 8	One-way ANOVA	****$p < 0.0001$ for WT+Tomato<br>n.s., $p = 0.3443$ for KE+Tomato<br>**$p < 0.01$ for KE+hPMCA2w/b	$F_{3, 24} = 16.79$<br>$F_{3, 40} = 1.141$<br>$F_{3, 28} = 6.951$
		Unpaired *t* test	**$p < 0.01$, *$p < 0.05$	
7J	WT + Tomato = 7<br>KE + Tomato = 11<br>KE + hPMCA2w/b = 8	One-way ANOVA	$p < 0.05$<br>Newman-Keuls multiple comparisons:<br>* $p < 0.05$	$F_{2, 23} = 5.143$
7K	WT + Tomato = 7<br>KE + Tomato = 11<br>KE + hPMCA2w/b = 8	One-way ANOVA	$p < 0.01$<br>Newman-Keuls multiple comparisons:<br>* $p < 0.05$, ** $p < 0.01$	$F_{2, 23} = 7.625$
